# Epidemiological characteristics and financial losses due to avian aspergillosis in households in the Almaty region, Republic of Kazakhstan

**DOI:** 10.3389/fvets.2023.1141456

**Published:** 2023-04-17

**Authors:** Dinara Kalkayeva, Amangeldi Maulanov, Przemysław Sobiech, Mirosław Michalski, Gulnur Kuzembekova, Ainur Dzhangabulova, Nurzhan Nurkhojayev, Nurbek Aldayarov

**Affiliations:** ^1^Department of Biological Safety, Veterinary Faculty, Kazakh National Agrarian Research University, Almaty, Kazakhstan; ^2^Department of Parasitology and Invasive Diseases, Faculty of Veterinary Medicine, University of Warmia and Mazury in Olsztyn, Olsztyn, Poland; ^3^Department of Microbiology, Virology and Immunology, Veterinary Faculty, Kazakh National Agrarian Research University, Almaty, Kazakhstan; ^4^Department of Veterinary Medicine, Agrarian Faculty, Mukhtar Auezov South Kazakhstan University, Shymkent, Kazakhstan; ^5^Department of Biology, Faculty of Sciences, Kyrgyz-Turkish Manas University, Bishkek, Kyrgyzstan

**Keywords:** epidemiological characteristics, financial losses, households, poultry, aspergillosis, Kazakhstan

## Abstract

Aspergillosis is a severe fungal disease that affects all species and ages of poultry and leads to significant economic losses within the poultry industry. The economic significance of aspergillosis is associated with direct losses due to poultry mortality, a decline in the production of meat and eggs, feed conversion, and poor growth of recovering poultry. Although a decrease in the production of poultry meat and eggs in Kazakhstan due to this fungal disease has been widely reported, studies on the consequent financial losses on affected farms (households) have not been carried out. This study aimed to estimate the financial losses and epidemiological parameters of avian aspergillosis among households affected by the disease in the Almaty region. To achieve the objectives of the research, a survey was conducted involving affected households from February 2018 to July 2019. The affected poultry were diagnosed based on clinical, macroscopical, and microscopical procedures, and once the infection was confirmed, household owners were interviewed. Data were collected from 183 household owners. The median incidence risk and fatality rates were 39 and 26% in chickens, 42 and 22% in turkeys, and 37 and 33% in geese, respectively, with young poultry having a higher incidence risk and fatality rate than adults. Approximately 92.4% of the household owners treated the affected poultry using natural folk methods and 7.6% of household owners used antifungal drugs and antibiotics, spending a median of US $35.20 (min US $0; max US $400) per household throughout the course of the infection. Egg production was reduced by a median of 58.3% when households were affected. The price of poultry fell by a median of 48.6% immediately after recovery due to weight loss. The median of the overall financial losses of households was US $198.50 (min US $11; max US $1,269). The majority of household owners (65%) did not replace their poultry, 9.8% of household owners replaced all their poultry, and the remaining 25.1% replaced only a proportion of the poultry lost at the time of the study. Newly acquired poultry were purchased from neighbors (10.9%), fellow villagers (50%), and state poultry farms (39.1%). This study demonstrates that aspergillosis has an immediate impact on subsistence household owners' livelihoods in the Almaty region of Kazakhstan.

## Introduction

The Republic of Kazakhstan (RK) is a country in the center of Eurasia with a territory of 2,724,902 km^2^. The population is 19,082,467 (1). The World Bank classifies the RK as a country with an upper middle-income economy (World Bank, 1 July 2021). Although agriculture remains a relatively unimportant sector (represents just 10% of GDP) of the Kazakh economy, it is considered the main source of income for the rural population which makes up 40.08% of the total population in the Republic. Currently, 45% of the total gross agricultural output involves livestock (1).

Poultry farming is one of the key sectors of the livestock industry in the RK, which ensures the country's food security. According to official data (1, 2), 65 poultry enterprises in the republic specialize in the production of eggs and poultry meat. Although the RK fully meets the demand of the domestic market for eggs (3), the domestic production of poultry meat covers only 58% of the needs of the domestic market.

Although poultry is predominantly produced by large poultry enterprises and farms, more than 25% of production is provided by households located in rural and urban areas (1) as an additional source of income and food. The market price of household poultry and associated products is higher than that of industrial producers. Unfortunately, infectious diseases with various geneses cause great economic damage to the poultry industry of the RK. An example is the recent outbreaks of the avian flu that swept across the country in 2020 (4, 5), where ~2 million chickens, geese, and ducks died.

Recently, affected poultry with clinical signs compatible with aspergillosis are often admitted to the Department of Pathology of the Kazakh National Agrarian Research University (KNARU) for postmortem examination and microscopic analysis. Due to the lack of clear clinical signs of avian aspergillosis, antemortem diagnosis is a challenge and unreliable (6).

Mycoses caused by fungi of the genus *Aspergillus* are common in humans (7–9), a wide range of animals (pet, farm, and wild) (10–16), and plants (17). Although there are several hundred species in the genus, subdivided into 22 distinct sections (18, 19), only 14 well-known species are infectious agents (6). Of these, a small number of opportunistic pathogens of the *Aspergillus* genus cover a wide range of diseases, ranging from localized infections to fatal disseminated diseases, as well as allergic responses to inhaled conidia (6, 7, 10, 20, 21). Moreover, some species belonging to the Aspergillus genus produce numerous mycotoxins (aflatoxins, gliotoxin, and ochratoxin A), which are released into the environment. Mycotoxins provide chemical protection and increase the virulence of the fungus (22–24). Feedstuff contaminated with Aspergillus mycotoxins is a cause of mortality in poultry. In terms of Aspergillosis cases, the most commonly found are caused by *A. fumigatus* (6, 20) and only a few by other species such as *A. flavus, A. niger*, and *A. terreus* (25–27).

In particular, *A. fumigatus* is the most common airborne fungal infection of the respiratory system of all avian species (10, 28–30). This is facilitated by the peculiarities of the anatomy and physiology of the avian lung-air sac system (31, 32). The relatively small spores of *A. fumigatus* (33) bypass initial physical barriers and penetrate deeply into the respiratory system to the air sacs where they are deposited (10). Furthermore, the epithelial surface of air sacs is almost devoid of mucociliary transport mechanisms (34). Consequently, poultry placed in a contaminated environment with aerosolized conidia may show significant pathology after only short exposure (6).

Aspergillosis occupies the first place in the structure of the mycotic pathologies of birds. This is due to the ubiquitous distribution of the *Aspergillus* species, the possibility of *Aspergillus* surviving on various biological substrates, as well as the morphological features of the fungi and their impact on the bird's organism (22, 35). The economic damage in this pathology is great since the mortality of young birds can range up to 90% (36, 37). Subsequent to data reported by Owings and Dykstra et al., the economic impact related to turkey mortality losses could be worth US $11 million annually. However, there have been no systematic studies on the prevalence of aspergillosis in chickens that would allow a substantiated estimate of the economic impact (38).

Keeping poultry benefit households through both income generation and as a direct source of quality food products for home consumption. Furthermore, households annually provide more than 10% of the market demand for eggs in the RK (1). Aspergillosis reduces meat and egg production, potentially having a negative impact on households' additional sources of income and food products. Nevertheless, the financial impact of avian aspergillosis in households in rural and urban areas of the RK has not been quantified, and disease control and prevention strategies used by the population are unknown.

The main purpose of this study is to assess the financial losses and infection parameters of avian aspergillosis in households in the Almaty region in terms of the affected poultry. Such a quantitative assessment in the endemic areas is critical for the development of disease control programs and for improving preparedness in other regions of the RK.

## Materials and methods

### Study area and households

**Figure 1:**
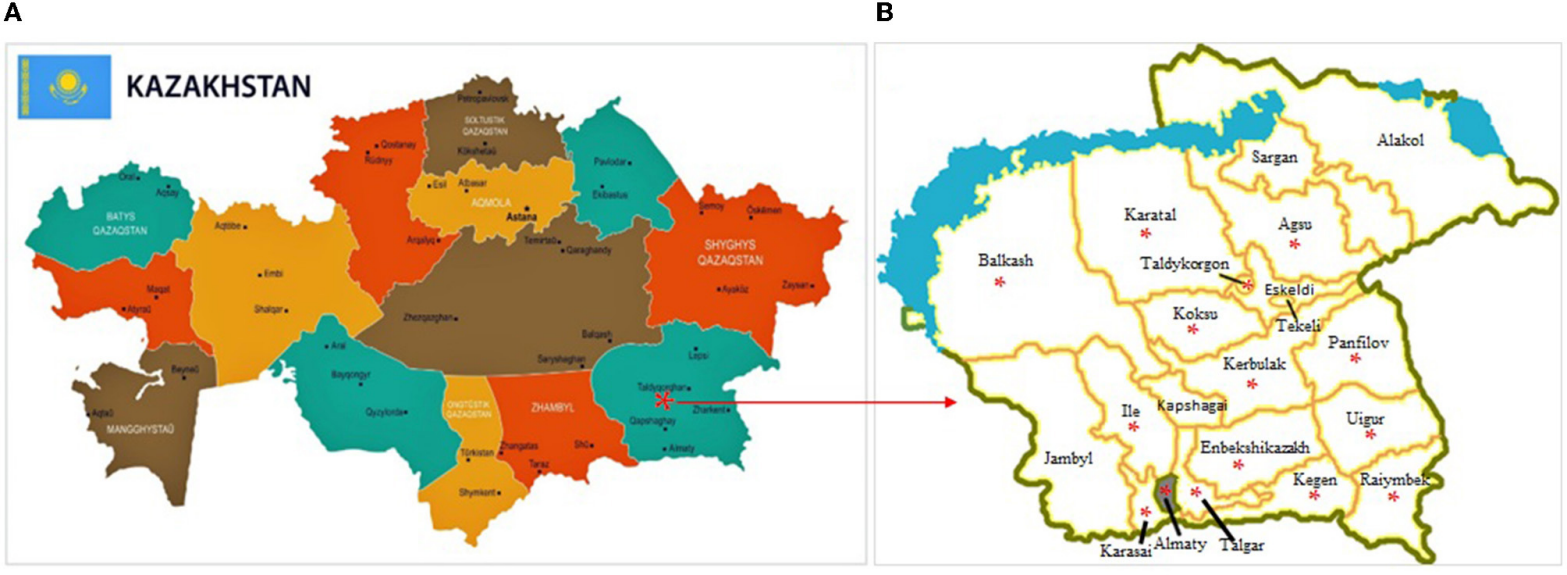
Administrative map of the Republic of Kazakhstan **(A)** (https://www.orangesmile.com/destinations/kazakhstan/country-maps-provinces.htm) and Almaty region **(B)** [(39), Almaty Oblast Aksu.png]. Areas where avian aspergillosis were reported to have been noted (*).

The study was conducted among households situated in 13 districts and two cities of the Almaty region from February 2018 to July 2019. The Almaty region is located in the southeast of the RK (Figure 1A). It is divided into 17 districts (Figure 1B) and contains three cities of regional subordination. Agriculture in the Almaty region is the largest sector in terms of employment (27.1% of total employment). There are more than 54,000 agricultural producers and 345,000 households make up the population. In terms of gross output, crop production is responsible for 50.4% and livestock, 49.2%. The region ranks first in the republic in terms of the number of cattle, horses, and poultry and leads in the production of meat (19%), milk (13%), wool (22%), and eggs (23%) (40).

The bulk of the rural and urban population, with the exception of officially registered agricultural producers, has an economy consisting mainly of livestock as their main or additional source of income. They have sheep, goats, cattle, horses, camels, and poultry in varying numbers. In general, with regard to poultry, they raise chickens, turkeys, ducks, geese, and others. According to official data (1), a significant number of livestock are maintained in rural households (52.4% cattle, 50.6% sheep, 56% pigs, 67.7% goats, and 25.2% poultry). In these households, there is a mix of all types of livestock, and there are practically no households keeping only one species of domestic animal. The urban population, given the limited conditions, keeps only poultry. In 2022, in all categories of farms in the RK, the poultry population amounted to 45.2 million heads. In total, 71.8% of them were concentrated in large poultry farms, 33.1% in households, and 1.4% in small farms.

### Study design and data collection

This study was based on a survey of 183 households, in which poultry were affected by avian aspergillosis. The affected poultry were identified following the household owners' report of an outbreak (or of sporadic cases) to a veterinarian. In addition, students from the KNARU have identified additional cases in their villages. All affected poultry were examined after the infection was reported and diagnosed by a qualified veterinarian. Avian aspergillosis was not registered among poultry raised in large enterprises and farms.

The affected poultry (chickens, turkeys, geese, and ducks), which demonstrated respiratory signs, dyspnea, rales, and weight loss, all died. Their age ranged from 6 days to 28 months. At postmortem examination, samples of organs affected with distinguishable aspergillosis lesions (e.g., granulomas, hemorrhages, and greenish-yellow cottony textures) were fixed in 4% formaldehyde and sent to KNARU in Almaty for the confirmation of the microscopical diagnosis. Paraffin wax-embedded specimens were sectioned for pathohistological examination at 5 μm and stained with hematoxylin and eosin for a general view. Caseous nodules and massive granulomas with necrotic cores surrounded by cells of lymphoid tissue (macrophages and lymphocytes), large foreign-body giant cells, and outer fibrous capsules were detected on the affected poultry organs. The results in terms of the above-described gross and microscopic lesions of avian aspergillosis were published in articles in local scientific journals. In total, 87% of the affected poultry were confirmed as displaying avian aspergillosis, and most of these birds (93%) were admitted from different areas of the Almaty region. Without exception, these were from households.

A total of 183 semi-structured interviews with affected household owners were conducted once the outbreak had concluded. All these owners have kept different species of poultry in their households for many years. The semi-structured interviews were conducted in a free and open manner. Before the interview, the goal of the study was explained to the respondents and verbal consent to participate was obtained. The duration of each interview was ~1.5 h. Details of the interviews were noted in notebooks and were recorded using Sony Dictaphones (ICD-BX140, China) or by using the dictaphone recorder function on the researchers' mobiles.

The basis of each interview was questions relating to aspects such as poultry breeding in the household, species of poultry, the total number of poultry, the purpose of keeping poultry, income from poultry products, poultry diseases, vaccination, treatment methods, and poultry fungal diseases (especially avian aspergillosis). In addition, information was obtained from the owners of the affected poultry with regard to morbidity and mortality, changes in production parameters, prices for healthy poultry (Supplementary Table 1), actions taken with regard to the affected poultry, and costs incurred. Interviews were conducted in Kazakh and Russian. A total of 183 household owners kindly agreed to participate in our interview. Data collection was carried out by the researchers, local veterinarians, and KNARU students.

### Data analysis

Data were entered into an Excel spreadsheet and inconsistencies across the data were cross-checked. Descriptive statistics were generated and stratified by household owner, species (chickens, turkeys, ducks, and geese), and age categories (young poultry <180 days and adults ≥180 days). Parameters estimated include incidence risk, fatality rate, treatment cost, the difference in price between healthy and affected (recovered) poultry, and reduction in egg production. Incidence risk and fatality rate were estimated as follows:


(1) $$\begin{array}{l} \text{Incident risk}\\ =\frac{\text{Number of new cases of aspergillosis in a specified period of time}}{\text{Number of aspergillosis free poultry at the start of the time period}} \end{array}$$

and


(2) $$\text{Fatality rate = }\frac{\text{Number of died poultry}}{\text{Number of poultry with clinical signs}}\;$$

The percentage reduction in poultry price and egg yield was calculated by comparing the estimated values in affected (recovered) and clinically healthy poultry. Price changes were assumed to be related to aspergillosis only.

### Estimation of total financial losses

**Figure 2:**
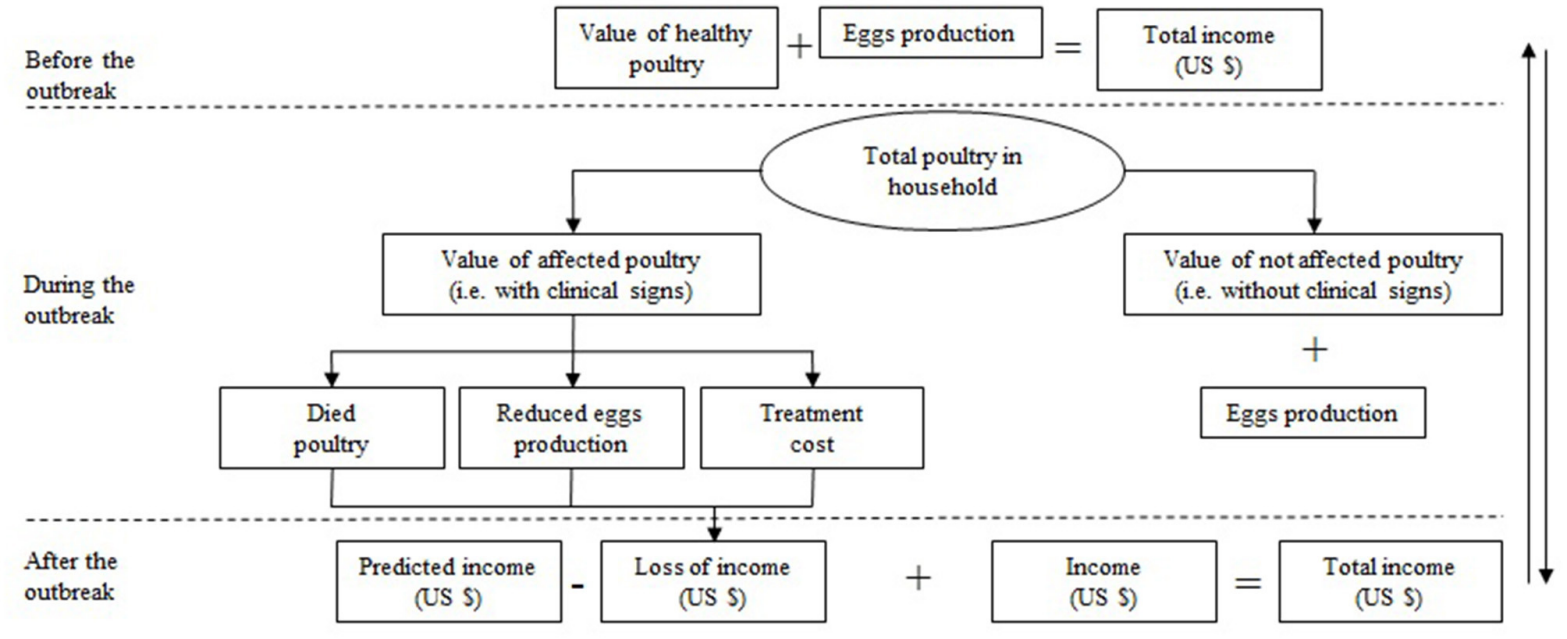
General scheme is used to estimate the financial losses caused by avian aspergillosis in households in the Almaty region of the RK.

Financial losses resulting from avian aspergillosis were calculated individually for each household. The main factors were the value of the poultry before the aspergillosis outbreak, the value of the poultry after the aspergillosis outbreak, treatment cost, income loss due to reduced egg production, and total financial losses. The estimation of financial losses was carried out according to the scheme presented in Figure 2.

First, the value of total poultry (+ eggs) before infection (VPBI) was estimated. The poultry number in the household before the infection equates to the sum of those poultry that presented with clinical signs, plus those that did not present any clinical signs. Owners of households reported the market price of each healthy poultry (eggs) at different ages. The total value of the poultry in a household was then estimated by multiplying the value of one bird by the number of birds stratified by species.


$$\text{VPBI = Pc}x\text{Tc + Pt}x\text{Tt + Pg}x\text{Tg+ Pd}x{\text{Td}}_{,}$$

where **P** – price, **T** – total number, **c** – chicken, **t** – turkey, **g** - goose, and **d** – duck.

To estimate the value of the affected poultry after the infection, we assumed that poultry were both affected (with clinical signs) and unaffected (without clinical signs). Affected poultry had two outcomes: they died or remained in the household. In the first case, the value of the poultry became zero due to mortality, and in the second case, the poultry recovered. However, the value of the poultry was lower than it would be if it had not been affected by the disease. To estimate the value of the poultry without clinical disease, we used the value that household owners reported they would be paid if they sold them as healthy.


VNAPAI = PcxNc + PtxNt + PgxNg + PdxNd,

where **VNAPAI** is the value of the not affected poultry kept after the infection, **N** is the number of poultry that did not present clinical signs during the infection;


$$VAPKAI=P^{\prime }cxN^{\prime }c+P^{\prime }txN^{\prime }t+P^{\prime }gxN^{\prime }g+P^{\prime }dxN^{\prime }d,$$

where **VAPKAI** is the value of the affected poultry kept after the infection, **P'** is the price affected poultry kept after the infection, **N'** is the number of the affected poultry kept after the infection;

Egg production losses due to clinical aspergillosis were estimated only for those household owners who reported selling eggs. The market price of eggs was also reported by the household owners.


ILREP=(EYBI-EYDI)xIDxEP,

where **ILREP** is the income loss due to reduced eggs production, **EYBI** is the daily egg yield before the infection, **EYDI** is the daily egg yield during the infection; **ID** is the infection duration; **EP** is the average egg price per piece in the study area.

The total value of the poultry after the infection was then estimated as the sum of the value of unaffected poultry in the household plus the value of affected poultry kept in the household until recovery.


VPAI=VNAPAI+VAPKAI,

where **VPAI** - is the total value of the poultry after the infection.

Treatment cost during the infection was equal to the money that household owners spent treating affected poultry with antifungal drugs and antibiotics or using folk remedies. Other expenses, such as time spent treating and looking after affected poultry, were not taken into consideration. Thus, the total financial losses per household owner were estimated as the difference between the value of the poultry before and after the infection, plus the treatment cost and income loss due to reduced egg production.


TFL=(VPBI--VPAI)+M+ILREP,

where **TFL** is the total financial losses, and **M** is the money spent on the treatment of poultry.

### Statistical analysis

Pearson's chi-squared test (or Fisher's Exact test where appropriate) was used to determine the strength of association between the binary outcomes of the two groups. For continuous variables, parametric (*t*-test or ANOVA) or non-parametric equivalent, if appropriate (Mann–Whitney U-test or Kruskal–Wallis tests), were used to compare the outcomes of different groups. A value of *p* < 0.005 was considered as being statistically significant. The analysis was performed using SPSS (23.0).

## Results

### Characteristics of households included in the study

A total of 201 affected household owners were identified and invited to take part in the study. Data were collected from 183 household owners who agreed to participate in the study. Data were obtained from 168 (91.8%) household owners from rural areas and 15 (8.2%) household owners from urban areas. A majority of the household owners were from the Enbekshikazakh district (*n* = 28; 15.3%), followed by Panfilov (*n* = 21; 11.5%), Raiymbek (*n* = 19; 10.4%), Agsu (*n* = 17; 9.3%), Karasai (*n* = 14; 7.6%), Talgar (*n* = 12; 6.5%), Kerbulak (*n* = 11; 6%), Karatal (*n* = 9; 4.9%), Uigur (*n* = 9; 4.9%), Kegen (*n* = 8; 4.4%), Ile (*n* = 7; 3.8%), Koksu (*n* = 7; 3.8%), Balkash (*n* = 6; 3.3%) districts, plus Almaty (*n* = 9; 4.9%) and Taldykorgon (*n* = 6; 3.3%) in the case of cities (Table 1). All household owners had other main jobs and kept domestic animals as an additional source of income. They have sheep, goats, cattle, horses, donkeys, chickens, turkeys, geese, and ducks in varying numbers. Each of the studied households simultaneously kept different species of livestock and poultry.

**Table 1:** The number of poultry in the households included in the study stratified by poultry species and districts (cities) in the Almaty region, RK.

Districts^*^and cities^**^in the Almaty region (number of households)	**Total number of poultry**	**Chicken**	**Turkey**	**Goose**	**Duck**
		**Total number/median value (Q1–Q3)**	**Total number/median value (Q1–Q3)**	**Total number/median value (Q1–Q3)**	**Total number/median value (Q1–Q3)**
All (183)	17,376	10,860**/**105 (24**–**86.5)	4,930**/**52 (13.75**–**58.75)	911**/**27.5 (5**–**18)	675**/**12 (7**–**20)
Enbekshikazakh^*^ (28)	2,928	1,525**/**71.5 (27.25**–**77.5)	1,097**/**37 (18**–**75.5)	123**/**9.5 (6.5**–**17)	184**/**8 (7**–**21)
Panfilov^*^ (21)	1,942	1,299**/**47 (47**–**85)	464**/**18 (18**–**33.25)	134**/**11 (11**–**17)	45**/**7 (7**–**12)
Raiymbek^*^ (19)	1,940	1,222**/**55 (37**–**84)	498**/**28 (21**–**56.25)	132**/**12,5 (4.5**–**18.25)	88**/**10 (5.25**–**14)
Agsu^*^ (17)	1,648	1,094**/**48 (23**–**109)	473**/**22 (10**–**40)	49**/**5.5 (2,75**–**12,5)	32**/**16 (0**–**18,5)
Karasai^*^ (14)	1,577	1,012**/**73.5 (36,75**–**102)	414**/**23.5 (14**–**46.25)	57**/**11 (4.5**–**18)	94**/**15 (13**–**16)
Talgar^*^ (12)	1,045	640**/**45 (27.5**–**72.25)	324**/**29 (16.5**–**38.75)	25**/**9 (3**–**11)	56**/**14 (4**–**26)
Kerbulak^*^ (11)	1,154	869**/**75 (42**–**115.5)	176**/**20.5 (7.75**–**31)	88**/**11 (6**–**17.5)	21**/**6 (3**–**9)
Karatal^*^ (9)	1,038	591**/**68 (29**–**79)	313**/**26 (12**–**71.5)	94**/**22 (7.5**–**23)	40**/**20 (0**–**25.5)
Uigur^*^ (9)	919	542**/**61 (30**–**83)	305**/**37 (8**–**74.5)	44**/**12 (2**–**21)	28**/**14 (0**–**18)
Kegen^*^ (8)	1,053	596**/**72.5 (49**–**82,5)	391**/**58 (25**–**72.5)	45**/**22.5 (0**–**27.75)	21**/**10.5 (0**–**10.75)
Ile^*^ (7)	459	241**/**18 (18**–**48.5)	149**/**34,5 (14.25**–**57)	58**/**9 (4.5**–**13)	11**/**11 (0**–**11)
Koksu^*^ (7)	757	607**/**99 (55**–**118.5)	110**/**10 (6.25**–**18)	28**/**14 (0**–**18.5)	12**/**12 (0**–**12)
Balkash^*^ (6)	716	423**/**67 (37.75**–**99)	216**/**98 (12**–**102)	34**/**9 (9**–**12.5)	43**/**21.5 (0**–**21.75)
Almaty^**^ (9)	113	113**/**14 (10.5**–**14)	**–**	**–**	**–**
Taldykorgon^**^ (6)	87	87**/**14.5 (12,75**–**16)	**–**	**–**	**–**

### Interviewed owners' reports

According to the reports, the majority of interviewed household owners (*n* = 168; 91.8%) kept two or more poultry species. A total of 15 (8.2%) household owners, due to limited opportunities, kept only a small number of chickens (from 7 to 17 heads). The total number of poultry in all households (*n* = 183; 100%) was 17,376, and the number of poultry in each household ranged from 7 to 162 head. In total, 13,504 heads of poultry were classified as adults (≥180 days) and 3,872 (<180 days) as young (Supplementary Tables 2–6). The poultry in the households were of different breeds.

The dominant type of poultry in the households were chickens (10,860, 62.5%) (Supplementary Table 3) and all owners (*n* = 183) kept them, followed by turkeys (*n* = 126; 4,930, 28.3%) (Supplementary Table 4), geese (*n* = 71; 911, 5.2%) (Supplementary Table 5), and ducks (*n* = 42; 675, 4%) (Supplementary Table 6). The most common reason for keeping poultry was its consumption (meat and eggs) at home (*n* = 66; 36.1%), commercialization of their meat, or its consumption at home (*n* = 48; 26.2%), sale of their eggs or their consumption at home (*n* = 26; 14.2%), selling them alive to earn income according to needs (*n* = 24; 13.1%), selling eggs (*n* = 16; 8.7%), and their commercialization regularly (*n* = 3; 1.6%). According to the report data, the average income from poultry (+eggs) annually was 14% of total income (min 1%; max 38%) (Supplementary Table 2).

In total, 61% (*n* = 112) of the interviewed household owners were aware of only two infections (Newcastle disease and avian flu diseases), while the rest (39%; *n* = 71) were unaware of any of the avian infectious diseases. Ectoparasites were known to all of the interviewed owners, and ascariasis was known to 44% (*n* = 81). Moreover, only one household owner (0.5%) was aware of avian aspergillosis because the owner had previously dealt with this pathology. Three (1.6%) of the interviewed household owners vaccinated chickens against Newcastle disease, while the rest (*n* = 180; 98.4%) did not. A total of 14 (7.6%) of the interviewed owners used antibiotics and antifungal drugs against different types of avian ailments. The dominant mass of household owners (*n* = 169; 92.4%) resorted to natural folk methods of treatment and prevention of illness in poultry. Most of them (*n* = 95; 56.2%) used crushed onion (*Allium cepa* L.; bulbs and leaves) and garlic (*Allium sativum* L.; bulbs and peduncles), mixed with feed. In total, 14.8% (*n* = 25) of the interviewed owners used only onion, 12.4% (*n* = 21) only garlic, 8.3% (*n* = 14) red peppers (*Capsicum annuum* L.), 5.9% (*n* = 10) root of harmala (*Peganum harmala* L.) (as an infusion or decoction), and 2.4% (*n* = 4) a small dose of childrens' urine (only in the case of turkeys) (Supplementary Table 2).

### Disease impact

The median infection duration was 11 days (min. 5; max. 26 days). In total, 48.1% (*n* = 88) of the household owners reported that only chickens were affected, and 8.2% reported that (*n* = 15) only turkeys were affected. While 30% (*n* = 55) had chicken and turkey affected, and 7.1% (*n* = 13) had chickens and geese affected. A total of 12 owners (6.5%) indicated that three poultry species (chicken, turkey, and goose) concurrently showed clinical signs and died. There were no reports of ducks affected by aspergillosis. The median infection duration was longer in households where more than one species of poultry was affected [22 days (min. 16; max. 26)] compared to those in which only one species was affected [9 days (min. 5; max. 12)]. The time between the end of the infection and the inspector's visit averaged 12 days (min 6; max 23).

Considering only infections reported between February 2018 and February 2019 (when materials and data on infections were collected throughout the year), infections were reported year-round with an increase between March and May and a second peak in the period from September to November (Supplementary Figure 1).

The median incidence risk and fatality rate were 39% (min 14; max 100) and 26% (min 5; max 100), respectively, in chicken, 42% (min 5; max 82) and 22% (min 6; max 85), respectively, in turkey, and 37% (min 21; max 80) and 33% (min 10; max 75), respectively, in geese. In young poultry (<180 days), the incidence risk [45 (min 1; max 100)] and fatality rate [58 (min 30; max 100)] were higher than in adult poultry (≥180 days) (32 (min 5; max 89) and 20 (min 4; max 78), respectively) (Table 2). The median yield in egg production per day before the infection was 74 (min 11; max 127) while after infection it was 42 (min 2; max 102), and the median drop in egg production during infection was 30 (min 6; max 57) in chicken, and 23.5 (min 12; max 106), 11 (min 7; max 100), and 10 (min 5; max 39) in turkey, respectively (Table 3, Supplementary Table 7).

**Table 2:** Median aspergillosis incidence risk and fatality rate in affected households in the Almaty region, RK.

**Number of households (n)**	**Age category**	**Chicken**					**Turkey**					**Goose**				
		***n***	**Incidence rick median (1st**−**3rd qtl)**	***p***	**Fatality rate median (1st**−**3rd qtl)**	**p**	***n***	**Incidence rick median (1st**−**3rd qtl)**	***p***	**Fatality rate median (1st**−**3rd qtl)**	***p***	***n***	**Incidence rick median (1st**−**3rd qtl)**	***p***	**Fatality rate median (1st**−**3rd qtl)**	***p***
183	All	168	39 (30–52)		26 (17–39)		82	42 (31.5–54.7)		22 (13–50)		25	37 (28.5–53)		33 (20–50)	
61	Young	61	51 (33.5–69)		59 (44–69)		24	60 (43–75.2)		67 (53.5–75.2)		13	52 (40–64)		50 (33–71)	
159	Adult	145	38 (30–50)	< 0.001	20 (14–28)	< 0.000	65	38 (28–53)	< 0.01	18 (12–25)	< 0.000	12	35 (23.7–56.7)	< 0.068	22.5 (16.2-27)	< 0.001

**Table 3:** Median egg yield before and after infection and daily drop in egg production during the aspergillosis reported by household owners in the Almaty region, RK.

**Number of households (n)**	**Poultry species**	**Median egg yield before infection (1st−3rd qtl)**	**Median egg yield after infection (1st−3rd qtl)**	**Median daily lost in egg production during infection (1st−3rd qtl)**
41	Chicken	74 (56–92)	42 (27–55)	30 (20.5–37)
10	Turkey	23.5 (15.7–52.7)	11 (7–30)	10 (6–21.5)

### Management and coping strategies

The majority of household owners (*n* = 155; 84.7%) treated affected poultry with folk methods of treatment, spending a median of $\bar{T}$1,760 (US$3.52) (min $\bar{T}$32; max $\bar{T}$8,000) per household per day (for purchases of garlic, onions, and hot chili peppers) and treated individual poultry for a median of 10 days (min 5; max 25 days). A total of 14 (7.6%) of the household owners did not spend any money as they used decoction or infusion of harmala (*n* = 10; 5.4%) and urine therapy (*n* = 4; 2.2%) for the treatment of the affected poultry. The remaining 14 (7.6%) household owners treated affected poultry with appropriate antifungal drugs and antibiotics prescribed by a veterinarian, spending a median of $\bar{T}$10,980 (US $21.96) (min $\bar{T}$10,980; max $\bar{T}$11,310). No information was collected regarding the time when treatment started in relation to the infection onset. The poultry displaying clinical signs were not sold or slaughtered for the sale of their meat.

The majority of household owners (*n* = 119; 65%) had not replaced poultry at the time of the interview. There were two main reasons for not replacing lost poultry. The first was the reluctance of owners to buy poultry from livestock markets because there was no guarantee that newly purchased poultry were healthy. The second reason was the lack of additional resources. A total of 18 household owners replaced all their poultry (9.8%), while the rest (*n* = 46; 25.1%) replaced only part of the lost poultry. Out of 64 household owners who provided an answer on the place where the last poultry had been purchased, 39 (60.9%) reported that it had been purchased from neighbors (*n* = 7; 10.9%) and fellow villagers (*n* = 32; 50%) who have a lot of poultry and good experience in keeping them, followed by state poultry farms (*n* = 25; 39.1%). Some of the household owners purchased poultry from more than one source. The median prices reported by household owners for the purchased poultry were $\bar{\text{T}}$3,800 (US$7.6) for chicken, $\bar{\text{T}}$10,600 (US$21.2) for turkey, and $\bar{\text{T}}$8,300 (US$16.6) for geese.

### Total financial losses during the infection

Financial losses during the infection are presented in Tables 4, 5. The median overall losses were $\bar{\text{T}}$98,400 (US$198.5), ranging from $\bar{\text{T}}$5,500 (US$11) to $\bar{\text{T}}$634,480 (US$1,269) (Table 4). The median losses were higher [$\bar{\text{T}}$231,470 (min $\bar{\text{T}}$93,960; max $\bar{\text{T}}$562,560)] when three poultry species (chicken, turkey, and goose) were affected, followed by chicken and turkey affected [$\bar{\text{T}}$ 193,040 (min $\bar{\text{T}}$23,460; max $\bar{\text{T}}$634,480)], only turkey affected [$\bar{\text{T}}$ 180,960 (min $\bar{\text{T}}$542,720; max $\bar{\text{T}}$26,850)], chicken and geese affected [$\bar{\text{T}}$ 70,240 (min $\bar{\text{T}}$16,899; max $\bar{\text{T}}$371,680)], and only chicken affected [$\bar{\text{T}}$ 42,930 (min $\bar{\text{T}}$5,500; max $\bar{\text{T}}$589,880)] (Table 5).

**Table 4:** Financial losses due to avian aspergillosis in households in the Almaty region, RK.

	**Median (min-max)**	
	**Kz** $\bar{\text{T}}$	**US $**
Value of the poultry before the infection	442,500 (27,000–166,8000)	885 (54–3,336)
Value of the poultry after the infection	373,500 (15,000–145,8000)	747 (30–2,916)
- Loss due to mortality	24,000 (3000–276,000)	48 (6–52)
- Value from affected poultry kept	38,000 (0–262,000)	76 (0–524)
- Value from unaffected poultry	316,500 (12,000–1,272,000)	641.2 (24–2,544)
Treatment cost	15,840 (0–200,000)	35.2 (0–44,582)
Money loss from the drop in egg production	17,310 (3,600–323,520)	34.6 (7.2–647)
Total loss	98,400 (5,500–634,480)	198.5 (11–1,269)
% of the value of the poultry loss	19,15 (1.6–79.9)	

**Table 5:** Total losses due to avian aspergillosis per bird by species affected.

	**Only chicken affected**	**Only turkey affected**	**Chicken and turkey affected**	**Chicken and goose affected**	**Chicken, turkey, and goose affected**
Number of households (n)	88	15	55	13	12
Total loss [Median (min-max)] $\bar{\text{T}}$	42,930 (5,500–589,880)	180,960 (542,720–26,850)	193,040 (23,460–634,480)	70,240 (16,899–371,680)	231,470 (93,960–562,560)
Total loss [Median (min-max)] $	102.4 (11–1,179.8)	361.9 (53.7–1,085.4)	386.1 (46.9– 1,269)	140.5 (33.8–743.4)	462.95 (187.9–1,125.1)

The median percentage loss in terms of the value of the poultry in households was 19.1% (min 1.6%; max 79.9%). The median percentage loss was higher for chicken and geese affected (median 27.3; min 6.5%, max 50%), followed by chicken and turkey affected (median 21.2; min 4%, max 48.3%), only turkey affected (median 20; min 6.1%; max 31.4%), chicken, turkey and geese affected (median 19; min 8.1%; max 32.5%), and only chicken affected (median 16.5; min 1.6%; max 79.9%) (Supplementary Table 8).

## Discussion

In this article, data are provided on financial losses and epidemiological parameters of avian aspergillosis among affected 183 households located in various villages in 13 districts and two cities of the Almaty region in Kazakhstan. The results of this research indicate that avian aspergillosis has an immediate impact on household owners' income. As mentioned above, 33.1% of all poultry in the republic is concentrated in households (1). Furthermore, poultry products produced in households are an organic source of poultry meat and eggs, as well as an additional source of income for the rural population.

Financial losses due to avian aspergillosis are associated with high mortality in the case of infected poultry, the disposal (non-sale) of carcasses of adult poultry, a decrease in egg production, and the purchase of medicines or raw materials for folk remedies.

The results of this study showed that the direct losses associated with the mortality (*p* < 0.05; *p* = 0.004) of poultry due to aspergillosis have an obvious immediate negative financial impact on the household owners and on the production of an organic source of poultry meat and eggs (*p* < 0.05; *p* = 0.00). The majority of household owners treated affected poultry with folk remedies, spending a median of US $31.5 (minimum $0.5; maximum $400.50). Some of the household owners treated the affected poultry with appropriate antifungals and antibiotics prescribed by a veterinarian, spending a median of US $65.90 (minimum $11.50; maximum $66.50). Thus, the treatment of affected poultry with modern medicines is confirmed statistically (*p* < 0.05; *p* = 0.01) as being two times more expensive than treatment with folk remedies. Median losses were higher (*p* < 0.05; *p* = 0.00) when three poultry species (chicken, turkey, and geese) were affected. The median percentage loss of poultry value in households was 19.1% (minimum 1.6%; maximum 79.9%). During the outbreak, the affected poultry and its meat were not sold, leading to a statistically significant reduction in the income (*p* < 0.05; *p* = 0.03) of the households, many of which, as indicated in Supplementary Table 2, build their daily income from the production of meat and eggs. In total, 65% (*n* = 119) of household owners reported that they had not replaced poultry that died as a consequence of the outbreak, citing a lack of assurance that healthy poultry could be purchased from livestock markets and a lack of additional resources. Interviewed owners reported that selling affected poultry or slaughtering for any ailments, including aspergillosis, was not carried out. This was because no one will buy diseased poultry, even at a very low price, and the carcasses of dead poultry were thrown in the trash or outer toilet, buried or burned, or given to dogs. Indeed, poultry carcasses resulting from airsacculitis are condemned at inspection following slaughter (41, 42).

Due to a lack of awareness, the household owners dump or give the poultry carcasses to their dogs, which increases the risk of spreading aspergillosis and increases the risk of infection among the owners themselves. As previously mentioned (43, 44), environmental contamination with *Aspergillus* conidia in poultry farms represents a significant risk for farm workers. Although the official RK veterinary authorities have developed rules for the prevention and elimination of avian aspergillosis (45), household owners were not aware of these rules. Poor awareness on the part of the household owners was confirmed by the results of this survey, where more than 61% of the owners had a superficial knowledge of only two avian infections, only one owner knew about avian aspergillosis, and only 1.6% of the owners had vaccinated chickens against Newcastle disease (Supplementary Table 2). This indicates that the veterinary service and local government authorities do not pay sufficient attention with regard to appropriate awareness-raising activities regarding the conditions required for poultry keeping and the general knowledge about the most common poultry diseases. To overcome these shortcomings, it is necessary to develop and distribute short and easily understandable brochures for ordinary people, organize special television programs, show videos on local channels, and hold local seminars.

According to the survey results, several (7.6%) household owners used antifungal drugs and additional antibiotics for the treatment of avian aspergillosis. Poultry farms have not treated birds with this pathology, although several strategies have been proposed for keeping birds in captivity. Furthermore, there were no appropriate vaccines (6, 46). In the current study, the majority of the interviewed owners (92.4%) prefer to use natural folk methods of treatment (Supplementary Table 2). They used freshly crushed onion (bulb and leaves) and garlic (bulb and peduncles) and red peppers mixed with feed, as well as harmala root as infusion or decoction and a small dose of fresh baby urine. Similar information on the treatment of avian aspergillosis was not found among other ethnoveterinary studies. In addition, more research is needed to specifically determine the effectiveness or otherwise of these remedies.

It is worth noting that all the above-mentioned ethnoveterinary methods of treatment are used among different ethnic groups for the treatment of other animal disorders. Onion has a wide array of uses in ethnoveterinary practices (47–49), ranging from gastrointestinal ailments, the treatment of tympany, indigestion, and bloating to proven insecticidal antiparasitic, repellant, antioxidant, anti-inflammatory, and antimicrobial actions (50, 51). Garlic also has effective pharmacological and medicinal properties (52) and is often used in veterinary practice for the treatment of various animal diseases (48, 49). The harmala is the most commonly used medicinal plant in ethnoveterinary practice (53). Harmala seed and root extracts have been reported to show antimicrobial, antiparasitic, antiviral, and antifungal activities (54). In addition, the results of many studies have proven that red pepper has many pharmacological properties (55). Interestingly, the internal and external application of one's own urine is an ancient Eastern tradition that is gaining popularity in the West (56). Human (56, 57) and animal (58) urine have multiple healing properties and has been noted for their antibacterial, antifungal, and antiviral properties (59). Thus, all of the above-mentioned plants and human urine, despite the lack of direct indications in studies on the treatment of avian aspergillosis, have antifungal properties.

In the current study, the correlation of the ethnoveterinary treatments with natural remedies, and the impact of avian aspergillosis, showed the following results. In the treatment of aspergillosis with onion + garlic, the mean mortality among adult poultry was 5.44 ± 5.22, among young poultry it was 15.85 ± 11.84, and the mean infection duration was 14.62 ± 6.86; with onion, it was 4.76 ± 3.64, 16.16 ± 5.67, and 15.84 ± 5.8, respectively; with garlic, it was 5.94 ± 4.94, 12.11 ± 6.8, and 14.76 ± 6.03, respectively; with red pepper, it was 4.53 ± 4.62, 13.25 ± 4.19, and 14.35 ± 6.23, respectively; with harmala decoction or infusion, it was 5 ± 3.6, 12.5 ± 2.12, and 15 ± 6.68, respectively; with urine, it was 3.66 ± 4.61, 16 ± 0, and 9 ± 0.81, respectively. In the treatment with the above-mentioned natural remedies between adult poultry and young poultry and infection duration, statistically, there were no significant differences (*p* > 0.05; *p* = 0.882, *p* = 0.658, and *p* = 0.561, respectively). However, there is a statistically significant difference between adult and young poultry (*p* > 0.05; *p* = 0.00).

In terms of the epidemiological parameters in the current study, the incidence risk and fatality rate were slightly different in chickens, turkeys, and geese (39–36%, 42–22%, and 37–33%, respectively). However, there is no statistically significant difference between the three groups of poultry (*p* > 0.05; *p* = 0.918 and *p* = 0.283, respectively). Incidence risk and fatality rates were higher in young poultry (45–58%) than in adults (20–32%). This difference was confirmed statistically (*p* < 0.05; both *p* = 0.00). Mortality ranged between 4.5 and 90%, while spontaneous avian aspergillosis in birds aged from 3 days to 20 weeks was previously reported (36, 37). This is due to the immaturity of the immune system in young individuals. The median yield in egg production during infection reduced by 59.5% and after infection by 41.7% in chickens, by 57.5 and 53.2% in turkeys, respectively, which indicates the marked and protracted negative impact of avian aspergillosis. The economic significance of aspergillosis was reported only in turkeys (60, 61), and it is especially important to note that it primarily affects expensive breeder toms (62).

In this pathology, the diagnosis was made using a combined method, since the antemortem diagnosis of aspergillosis is considered difficult and unreliable (6, 63, 64). This refers to clinical signs, postmortem, and microscopic features with the mandatory detection of conidia and fungal culture. However, the lack of molecular methods for diagnosing avian aspergillosis and the shortage of local pathologists in Kazakhstan cause great problems when it comes to a correct diagnosis.

It is clear from the results presented here that avian aspergillosis has a negative financial impact on household owners' livelihoods. Furthermore, coping, treatment and prevention strategies, and control measures are not well organized by the official veterinary authorities, which is likely to have consequences for spreading Aspergillus in the study area. It is necessary to raise household owners' awareness of the necessary conditions of poultry keeping and feeding and feed storage and to provide general information with regard to poultry diseases, including avian aspergillosis.

To effectively control this disease, it is recommended that there is a need to develop a structural strategic plan that will include informational materials and training sessions for household owners and poultry farmers. For the timely and correct diagnosis of avian aspergillosis, it is necessary to provide local veterinary laboratories with modern culture, histology, serology, imaging, and molecular techniques and the hiring of competent specialists. It is also important to take into consideration the above-mentioned folk methods of treatment used by the household owners; further study of non-traditional methods of treatment could provide a significant breakthrough in the veterinary treatment of aspergillosis.

## Conclusion

This study demonstrates that aspergillosis has an immediate impact on subsistence household owners' livelihoods in the Almaty region of the Republic of Kazakhstan. We have quantified the effect of aspergillosis on production parameters that have not been quantified before in chickens, turkeys, and geese and have assessed the impact of the diseases on subsistence producers from different angles. We have also identified potential transmission routes and areas where appropriate control measures should be directed.

## Data availability statement

The original contributions presented in the study are included in the article/Supplementary material, further inquiries can be directed to the corresponding authors.

## Ethics statement

The animal study was reviewed and approved by Ethics Committee of the Kazakh National Agrarian Research University.

## Author contributions

DK and NA conceptualized the study and developed the questionnaire with input from AM, PS, MM, GK, AD, and NN. AM and DK diagnosed affected poultry, according to the results of necropsy and microscopic examination. DK coordinated the data collection (with input from AM) and performed the data entry. NA verified data entry and conducted data analysis with input from DK. DK secured funding. AM, DK, and NA drafted the manuscript. All authors reviewed the manuscript.
