# Supplementary material for: Epidemiological characteristics and financial losses due to avian aspergillosis in households in the Almaty region, Republic of Kazakhstan
Source: Front Vet Sci. 2023 Apr 17;10:1141456. doi: 10.3389/fvets.2023.1141456 (PMC10150056; doi:10.3389/fvets.2023.1141456)
Supplement: Supplementary file 1 [file Data_Sheet_1.docx]

Supplementary Material

***TABLE S1* Prices for healthy poultry by species and age categories** **in the Almaty region reported by household owners**

| **Age category** | **Price per head of poultry** (₸ / $) | | | |
| --- | --- | --- | --- | --- |
|  | **Chicken** | **Turkey** | **Goose** | **Duck** |
| 7 days | 400 / 0.8 | 900 / 1.8 | 2000 / 4 | 600 / 1.2 |
| 14 days | 500 / 1 | 1000 / 2 | 2200 / 4.4 | 700 / 1.4 |
| 21 days | 600 / 1.2 | 1500 / 3 | 2500 / 5 | 800 / 1.6 |
| 30 days | 700 / 1.4 | 1800 / 3.6 | 3000 / 6 | 900 / 1.8 |
| 45 days | 800 / 1.6 | 2000 / 4 | 3300 / 6.6 | 1000 / 2 |
| 60 days | 1000 / 2 | 2500 / 5 | 3500 / 7 | 1100 / 2.2 |
| 90 days | 1500 / 3 | 3000 / 6 | 4000 / 8 | 1200 / 2.4 |
| 120 days | 2000 / 4 | 3500 / 7 | 4500 / 9 | 1300 / 2.6 |
| 150 days | 2500 / 5 | 4000 / 8 | 5000 / 10 | 1400 / 2.8 |
| **≤** 180 days | 3000-4000 / 6-8 | 5000-12000 / 10-24 | 6000-10000 / 12-20 | 1500-3000 / 3-6 |

₸ - Kazakh tenge; $ - US dollar

**TABLE S2 Household owner survey reports**

| **Interviewed owner code name** | **Date of detection of aspergillosis (duration of infection) /Address** | **Total number of poultry** (^c^chicken, ^t^turkey, ^g^goose, ^d^duck) | **Age category** (**^i^**young < 180 days; **^ii^**adult ≥180 days) | **Reason for keeping poultry** | **Average income** (%) | **Affected poultry**  (with clinical signs **/**  died) | **Treatment method** | **Which of the poultry diseases was the household owner aware of?** | **Poultry vaccination** |
| --- | --- | --- | --- | --- | --- | --- | --- | --- | --- |
| Al_Cit_01 | February 2018 (5 days) / Almaty city | ^c^14 | ^ii^14 | To consume them at home | 2 | ^c,ii^4 / ^c,ii^1 | Folk methods were used (crushed onion (bulbs and leaves) and garlic (bulbs and peduncles) mixed with feed) | Ectoparasites | No |
| Al_Cit_02 | February 2018 (9 days) / Almaty city | ^c^15 | ^ii^15 | To consume them at home | 1 | ^c,ii^4 / ^c,ii^2 | Folk methods were used (crushed onion (bulbs and leaves) mixed with feed) | Ectoparasites | No |
| Tn_Cit_03 | March 2018 (7 days) /  Taldykorgon city | ^c^13 | ^ii^13 | To consume them at home | 2 | ^c,ii^5 / ^c,ii^1 | Folk methods were used (crushed onion (bulbs and leaves) and garlic (bulbs and peduncles) mixed with feed) | Newcastle disease and avian flu; Ectoparasites | No |
| En_She_04 | March 2018 (24 days) / Enbekshikazakh district, Shelek village | 84 (^c^56, ^t^28) | ^ii^84 | To commercialize their meat or consume it at home | 6 | ^c,ii^9 / ^c,ii^3;  ^t,ii^9 / ^t,ii^2 | Folk methods were used (crushed red peppers mixed with feed) | Newcastle disease and avian flu; Ectoparasites | No |
| En_Tes_05 | March 2018 (21 days) / Enbekshikazakh district, Teskensu village | 152 (^c^59, ^t^43, ^g^23, ^d^27) | ^ii^152 | To sell them live to earn income according to needs | 18 | ^c,ii^10 / ^c,ii^2;  ^t,ii^12 / ^t,ii^1 | Folk methods were used (crushed garlic (bulbs and peduncles) mixed with feed) | Newcastle disease and avian flu; Ectoparasites | No |
| En_Bir_06 | March 2018 (11 days) / Enbekshikazakh district, Birlik village | 47 (^c^35, ^t^12) | ^ii^47 | To consume them at home | 3 | ^c,ii^10 / ^c,ii^1 | Folk methods were used (crushed onion (bulbs and leaves) and garlic (bulbs and peduncles) mixed with feed) | Ectoparasites | No |
| Pa_Sar_07 | March 2018 (26 days) / Panfilov district, Sarybel village | 68 (^c^36, ^t^18, ^g^14) | ^ii^68 | To consume them at home | 4 | ^c,ii^12 / ^c,ii^1  ^t,ii^10 / ^t,ii^2 | Folk methods were used (crushed onion (bulbs and leaves) mixed with feed) | Newcastle disease and avian flu; Ectoparasites and ascariasis | No |
| Pa_Sar_08 | March 2018 (22 days) / Panfilov district, Sarybel village | 93 (^c^42, ^t^34, ^g^17) | ^ii^93 | To consume them at home | 13 | ^c,ii^12 / ^c,ii^2;  ^t,ii^8 / ^t,ii^3;  ^g,ii^4 / ^g,ii^1 | Folk methods were used (crushed garlic (bulbs and peduncles) mixed with feed) | Newcastle disease and avian flu; Ectoparasites | No |
| Tr_Erk_09 | March 2018 (23 days) / Talgar district, Erkin village | 58 (^c^21, ^t^23, ^d^14) | ^ii^58 | To commercialize their meat or consume it at home | 4 | ^c,ii^14 / ^c,ii^1;  ^t,ii^10 / ^t,ii^1 | Folk methods were used (crushed onion (bulbs and leaves) and garlic (bulbs and peduncles) mixed with feed) | Newcastle disease and avian flu; Ectoparasites and ascariasis | No |
| Ra_Sar_010 | March 2018 (9 days) /  Raiymbek district, Saryzhaz village | 142 (^c^66, ^t^45, ^g^19, ^d^12) | ^ii^142 | To sell them live to earn income according to needs | 7 | ^c,ii^17 / ^c,ii^3 | Folk methods were used (crushed garlic (bulbs and peduncles) mixed with feed) | Newcastle disease and avian flu; Ectoparasites and ascariasis | No |
| En_Kar_011 | March 2018 (11 days) / Enbekshikazakh district, Karaturyk village | 29 (^c^18, ^t^7, ^g^4) | ^ii^29 | To consume them at home | 1 | ^c,ii^6 / ^c,ii^1 | Folk methods were used (crushed onion (bulbs and leaves) mixed with feed) | Newcastle disease and avian flu; Ectoparasites and ascariasis | No |
| Ra_Kai_012 | March 2018 (22 days) / Raiymbek district, Kainar village | 162 (^c^55, ^t^107) | ^ii^162 | To sell them on a regular basis | 38 | ^c,ii^19 / ^c,ii^5;  ^t,ii^19 / ^t,ii^2 | Appropriate antifungal drugs and antibiotics were used | Newcastle disease and avian flu; Ectoparasites | Yes (against Newcastle disease) |
| Ks_Ayt_013 | March 2018 (24 days) / Karasai district, Aytey village | 89 (^c^57, ^t^14, ^g^18) | ^ii^89 | To commercialize their meat or consume it at home | 9 | ^c,ii^19 / ^c,ii^3 ;  ^t,ii^4 / ^t,ii^1 | Folk methods were used (crushed onion (bulbs and leaves) and garlic (bulbs and peduncles) mixed with feed) | Newcastle disease and avian flu; Ectoparasites and ascariasis | No |
| Kk_Kyz_014 | March 2018 (26 days) / Kerbulak district, Kyzylzhar village | 114 (^c^66, ^t^31, ^g^17) | ^ii^114 | To sell them live to earn income according to needs | 23 | ^c,ii^26 / ^c,ii^7;  ^t,ii^18 / ^t,ii^2 | Folk methods were used (crushed onion (bulbs and leaves) and garlic (bulbs and peduncles) mixed with feed) | Newcastle disease and avian flu; Ectoparasites and ascariasis | No |
| Ag_Suy_015 | March 2018 (9 days) /  Agsu district, Suyksay village | 32 (^c^24, ^t^8) | ^ii^32 | To consume them at home | 2 | ^c,ii^5 / ^c,ii^1 | Folk methods were used (crushed red peppers mixed with feed) | Newcastle disease and avian flu; Ectoparasites | No |
| En_Kyz_016 | March 2018 (10 days) / Enbekshikazakh district, Kyzylsharyk village | 94 (^c^23, ^t^71) | ^ii^94 | To commercialize their meat or consume it at home | 18 | ^t,ii^25 / ^t,ii^7 | Appropriate antifungal drugs and antibiotics were used | Newcastle disease and avian flu; Ectoparasites and ascariasis | No |
| Ag_Oyt_017 | March 2018 (11 days) / Agsu district, Oytogan village | 104 (^c^88, ^t^16) | ^ii^104 | To commercialize their eggs or consume it at home | 21 | ^c,ii^27 / ^c,ii^4 | Folk methods were used (root of harmala; infusion) | Newcastle disease and avian flu; Ectoparasites | No |
| En_She_018 | March 2018 (20 days) / Enbekshikazakh district, Shelek village | 48 (^c^35, ^g^13) | ^ii^9 (^c^7, ^g^2);  ^i^39 (^c^28, ^g^11) | To consume them at home | 1 | ^c,i^26 / ^c,i^11;  ^g,i^7 / ^g,i^5 | Folk methods were used (crushed garlic (bulbs and peduncles) mixed with feed) | Newcastle disease and avian flu; Ectoparasites and ascariasis | No |
| Kt_Ayt_019 | March 2018 (7 days) /  Karatal district, Aytubiy village | 147 (^c^136, ^t^11) | ^ii^61 (^c^49, ^t^11);  ^i^87 (^c^87) | To commercialize their meat or consume it at home | 22 | ^c,ii^9 / ^c,ii^3;  ^c,i^25 / ^c,i^17 | Folk methods were used (crushed onion (bulbs and leaves) and garlic (bulbs and peduncles) mixed with feed) | Newcastle disease and avian flu; Ectoparasites and ascariasis | No |
| Ks_Zha_020 | March 2018 (12 days) / Karasai district, Zhalpaksay village | 129 (^c^113, ^d^16) | ^ii^15 (^c^11, ^d^4);  ^i^114 (^c^102, ^d^12) | To commercialize their meat or consume it at home | 11 | ^c,i^51 / ^c,i^39 | Folk methods were used (crushed onion (bulbs and leaves) and garlic (bulbs and peduncles) mixed with feed) | Newcastle disease and avian flu; Ectoparasites and ascariasis | No |
| Ui_Dol_021 | April 2018 (11 days) /  Uigur district, Dolayty village | 153 (^c^91, ^t^62) | ^ii^153 | To sell them live to earn income according to needs | 16 | ^t,ii^14 / ^t,ii^1 | Folk methods were used (crushed onion (bulbs and leaves) and garlic (bulbs and peduncles) mixed with feed) | Newcastle disease and avian flu; Ectoparasites and ascariasis | No |
| Pa_Bas_022 | April 2018 (12 days) /  Panfilov district, Baskunchin village | 86 (^c^80, ^t^6) | ^ii^86 | To sell eggs | 23 | ^c,ii^21 / ^c,ii^4 | Folk methods were used (crushed red peppers mixed with feed) | Ectoparasites | No |
| En_Bay_023 | April 2018 (23 days) /  Enbekshikazakh district, Bayseit village | 54 (^c^36, ^t^18) | ^ii^11 (^c^9, ^t^2);  ^i^43 (^c^27, ^t^16) | To consume them at home | 1 | ^c,i^14 / ^c,i^8;  ^t,i^12 / ^t,i^6 | Folk methods were used (crushed onion (bulbs and leaves) mixed with feed) | Newcastle disease and avian flu; Ectoparasites | No |
| Ag_Egi_024 | April 2018 (9 days) /  Agsu district, Eginsu village | 139 (^c^52, ^t^87) | ^ii^103 (^c^16, ^t^87);  ^i^36 (^c^36) | To commercialize their meat or consume it at home | 14 | ^c,ii^6 / ^c,ii^1;  ^c,i^17 / ^c,i^10 | Folk methods were used (crushed garlic (bulbs and peduncles) mixed with feed) | Newcastle disease and avian flu; Ectoparasites and ascariasis | No |
| Ra_Kai_025 | April 2018 (8 days) /  Raiymbek district, Kainar village | 44 (^c^41, ^g^3) | ^ii^44 | To commercialize their eggs or consume it at home | 3 | ^c,ii^15 / ^c,ii^3 | Appropriate antifungal drugs and antibiotics were used | Newcastle disease and avian flu; Ectoparasites and ascariasis | No |
| Ui_Sum_026 | April 2018 (9 days) /  Uigur district, Sumbe village | 53 (^c^47, ^t^4, ^g^2) | ^ii^12 (^c^6, ^t^4, ^g^2);  ^i^41 (^c^41) | To consume them at home | 2 | ^c,i^11 / ^c,i^11 | Folk methods were used (root of harmala; decoction) | Newcastle disease and avian flu; Ectoparasites and ascariasis | No |
| Tr_Kis_027 | April 2018 (11 days) /  Talgar district, Kishi Bayserke village | 71 (^c^65, ^t^6) | ^ii^23 (^c^17, ^t^6);  ^i^48 (^c^48) | To consume them at home | 4 | ^c,ii^11 / ^c,ii^3;  ^c,i^25 / ^c,i^15 | Folk methods were used (crushed onion (bulbs and leaves) and garlic (bulbs and peduncles) mixed with feed) | Newcastle disease and avian flu; Ectoparasites and ascariasis | No |
| Ag_Egi_028 | April 2018 (12 days) /  Agsu district, Eginsu village | 25 (^c^22, ^g^3) | ^ii^25 | To consume them at home | 1 | ^c,ii^6 / ^c,ii^2 | Folk methods were used (crushed garlic (bulbs and peduncles) mixed with feed) | Newcastle disease and avian flu;  Ectoparasites and ascariasis | No |
| Il_Bor_029 | April 2018 (9 days) /  Ile district, Boraldai village | 33 (^c^18, ^t^15) | ^ii^33 | To consume them at home | 2 | ^t,ii^8 / ^t,ii^1 | Folk methods were used (small dose of children's urine, perorally) | Newcastle disease and avian flu; Ectoparasites and ascariasis | No |
| Kn_Zhy_030 | April 2018 (25 days) /  Kegen district, Zhylysay village | 134 (^c^66, ^t^58, ^d^10) | ^ii^134 | To commercialize their meat or consume it at home | 14 | ^c,ii^26 / ^c,ii^6;  ^t,ii^15 / ^t,ii^1 | Folk methods were used (crushed onion (bulbs and leaves) and garlic (bulbs and peduncles) mixed with feed) | Newcastle disease and avian flu; Ectoparasites and ascariasis | No |
| Pa_Kon_031 | April 2018 (8 days) /  Panfilov district, Konyrolen village | 88 (^c^85, ^g^3) | ^ii^88 | To commercialize their eggs or consume it at home | 15 | ^c,ii^20 / ^c,ii^1 | Folk methods were used (crushed onion (bulbs and leaves) and garlic (bulbs and peduncles) mixed with feed) | Newcastle disease and avian flu; Ectoparasites | No |
| Pa_Kon_032 | April 2018 (9 days) /  Panfilov district, Konyrolen village | 61 (^c^44, ^t^8, ^g^2, ^d^7) | ^ii^22 (^c^11, ^t^8, ^g^2, ^d^1);  ^i^39 (^c^33, ^d^6) | To consume them at home | 2 | ^c,i^15 / ^c,i^12 | Folk methods were used (crushed onion (bulbs and leaves) and garlic (bulbs and peduncles) mixed with feed) | Newcastle disease and avian flu; Ectoparasites and ascariasis | No |
| En_Sar_033 | April 2018 (10 days) /  Enbekshikazakh district, Sarybulak village | 94 (^c^58, ^t^36) | ^ii^94 | To commercialize their meat or consume it at home | 8 | ^t,i^14 / ^t,i^11 | Folk methods were used (crushed onion (bulbs and leaves) and garlic (bulbs and peduncles) mixed with feed) | Newcastle disease and avian flu; Ectoparasites | No |
| Kk_Kog_034 | April 2018 (9 days) /  Kerbulak district, Kogalin village | 155 (^c^101, ^t^31, ^g^11, ^d^12) | ^ii^92 (^c^46, ^t^31, ^g^11, ^d^4);  ^i^63 (^c^55, ^d^8) | To commercialize their meat or consume it at home | 13 | ^c,i^22 / ^c,i^16 | Folk methods were used (crushed garlic (bulbs and peduncles) mixed with feed) | Newcastle disease and avian flu; Ectoparasites and ascariasis | No |
| Tr_Bes_035 | April 2018 (19 days) /  Talgar district, Besagash village | 153 (^c^112, ^t^41) | ^ii^153 | To sell them live to earn income according to needs | 20 | ^c,ii^17 / ^c,ii^4;  ^t,ii^10 / ^t,ii^2 | Folk methods were used (crushed onion (bulbs and leaves) mixed with feed) | Newcastle disease and avian flu; Ectoparasites and ascariasis | No |
| Ku_Zha_036 | April 2018 (11 days) /  Koksu district, Zharlyozek village | 69 (^c^62, ^t^7) | ^ii^69 | To sell them live to earn income according to needs | 9 | ^c,ii^26 / ^c,ii^5 | Folk methods were used (crushed red peppers mixed with feed) | Newcastle disease, avian flu and **aspergillosis**; Ectoparasites | No |
| Al_Cit_037 | April 2018 (11 days) /  Almaty city | ^c^12 | ^ii^12 | To consume them at home | 1 | ^c,ii^3 / ^c,ii^2 | Folk methods were used (crushed onion (bulbs and leaves) and garlic (bulbs and peduncles) mixed with feed) | Newcastle disease and avian flu; Ectoparasites and ascariasis | No |
| Kn_Sat_038 | April 2018 (5 days) /  Kegen district, Satin village | 46 (^c^35, ^d^11) | ^ii^46 | To sell eggs | 8 | ^c,ii^15 / ^c,ii^1 | Folk methods were used (crushed onion (bulbs and leaves) and garlic (bulbs and peduncles) mixed with feed) | Newcastle disease and avian flu; Ectoparasites | No |
| Kt_Bal_039 | April 2018 (8 days) /  Karatal district, Balpyk village | 133 (^c^27, ^t^106) | ^ii^133 | To commercialize their meat or consume it at home | 14 | ^t,ii^30 / ^t,ii^5 | Appropriate antifungal drugs and antibiotics were used | Newcastle disease and avian flu; Ectoparasites and ascariasis | No |
| Kn_Sat_040 | May 2018 (22 days) /  Kegen district, Satin village | 148 (^c^52, ^t^63, ^g^33) | ^ii^148 | To sell them live to earn income according to needs | 32 | ^c,ii^21 / ^c,ii^4;  ^t,ii^14 / ^t,ii^3 | Folk methods were used (crushed garlic (bulbs and peduncles) mixed with feed) | Newcastle disease and avian flu; Ectoparasites | No |
| Kk_Zho_041 | May 2018 (11 days) /  Kerbulak district, Zholaman village | 29 (^c^23, ^t^6) | ^ii^29 | To consume them at home | 1 | ^c,ii^12 / ^c,ii^2 | Folk methods were used (crushed onion (bulbs and leaves) and garlic (bulbs and peduncles) mixed with feed) | Ectoparasites | No |
| Ba_Aszl_042 | May 2018 (9 days) /  Balkash district, Akzhar village | 128 (^c^22, ^t^106) | ^ii^128 | To commercialize their meat or consume it at home | 26 | ^t,ii^18 / ^t,ii^2 | Folk methods were used (root of harmala; decoction) | Newcastle disease and avian flu; Ectoparasites | No |
| Ra_Sat_043 | May 2018 (8 days) /  Raiymbek district, Satin village | 149 (^c^143, ^t^6) | ^ii^97 (^c^91, ^t^6);  ^i^52 (^c^52) | To sell them live to earn income according to needs | 18 | ^c,ii^27 / ^c,ii^5;  ^c,i^13 / ^c,i^9 | Folk methods were used (crushed onion (bulbs and leaves) and garlic (bulbs and peduncles) mixed with feed) | Newcastle disease and avian flu; Ectoparasites and ascariasis | No |
| Al_Cit_044 | May 2018 (10 days) /  Almaty city | ^c^7 | ^ii^7 | To consume them at home | 1 | ^c,ii^4 / ^c,ii^1 | Folk methods were used (crushed onion (bulbs and leaves) and garlic (bulbs and peduncles) mixed with feed) | Newcastle disease and avian flu; Ectoparasites | No |
| En_Bay_045 | May 2018 (12 days) /  Enbekshikazakh district, Bayseit village | 45 (^c^39, ^t^6) | ^ii^45 | To sell eggs | 5 | ^c,ii^21 / ^c,ii^2 | Folk methods were used (root of harmala; decoction) | Newcastle disease and avian flu; Ectoparasites | No |
| Il_Ash_046 | May 2018 (19 days) /  Ile district, Aschibulak village | 93 (^c^18, ^t^66, ^g^9) | ^ii^93 | To commercialize their meat or consume it at home | 16 | ^c,ii^6 / ^c,ii^1;  ^t,ii^25 / ^t,ii^3 | Folk methods were used (crushed onion (bulbs and leaves) and garlic (bulbs and peduncles) mixed with feed) | Newcastle disease and avian flu; Ectoparasites and ascariasis | No |
| Ba_Zhi_047 | May 2018 (11 days) /  Balkash district, Zhidelin village | 65 (^c^44, ^d^21) | ^ii^18 (^c^13, ^d^5);  ^i^47 (^c^31, ^d^16) | To consume them at home | 3 | ^c,ii^8 / ^c,ii^3;  ^c,i^9 / ^c,i^5 | Folk methods were used (crushed garlic (bulbs and peduncles) mixed with feed) | Newcastle disease and avian flu; Ectoparasites and ascariasis | No |
| Pa_Ulk _048 | May 2018 (7 days) /  Panfilov district, Ulkenagash village | 115 (^c^104, ^t^11) | ^ii^115 | To sell eggs | 20 | ^c,ii^35 / ^c,ii^8 | Folk methods were used (crushed onion (bulbs and leaves) and garlic (bulbs and peduncles) mixed with feed) | Newcastle disease and avian flu; Ectoparasites | No |
| Kt_Tas_049 | May 2018 (19 days) /  Karatal district, Tastobin village | 148 (^c^43, ^t^83, ^g^22) | ^ii^148 | To commercialize their meat or consume it at home | 23 | ^c,ii^12 / ^c,ii^3;  ^t,ii^20 / ^t,ii^6 | Folk methods were used (crushed onion (bulbs and leaves) mixed with feed) | Newcastle disease and avian flu; Ectoparasites and ascariasis | No |
| Ra_Bol_050 | May 2018 (9 days) /  Raiymbek district, Boleksaz village | 137 (^c^124, ^d^13) | ^ii^65 (^c^63, ^d^2);  ^i^72 (^c^61, ^d^11) | To commercialize their eggs or consume it at home | 9 | ^c,ii^18 / ^c,ii^1;  ^c,i^20 / ^c,i^13 | Folk methods were used (crushed onion (bulbs and leaves) and garlic (bulbs and peduncles) mixed with feed) | Newcastle disease and avian flu; Ectoparasites | No |
| Ag_Egi_051 | May 2018 (5 days) /  Agsu district, Eginsu village | 69 (^c^48, ^d^21) | ^ii^18 (^c^23, ^d^5);  ^i^41 (^c^25, ^d^16) | To consume them at home | 2 | ^c,ii^5 / ^c,ii^1;  ^c,i^13 / ^c,i^8 | Folk methods were used (crushed onion (bulbs and leaves) and garlic (bulbs and peduncles) mixed with feed) | Newcastle disease and avian flu; Ectoparasites | No |
| Kk_Kyz_052 | May 2018 (11 days) /  Kerbulak district, Kyzylzhar village | 133 (^c^124, ^t^6, ^d^3) | ^ii^75 (^c^66, ^t^6, ^d^3);  ^i^58 (^c^58) | To sell eggs | 21 | ^c,ii^16 / ^c,ii^2;  ^c,i^20 / ^c,i^11 | Folk methods were used (crushed onion (bulbs and leaves) and garlic (bulbs and peduncles) mixed with feed) | Newcastle disease and avian flu; Ectoparasites and ascariasis | No |
| Pa_Bir_053 | June 2018 (24 days) /  Panfilov district, Birlik village | 52 (^c^37, ^t^15) | ^ii^17 (^c^15, ^t^2);  ^i^35 (^c^22, ^t^13) | To consume them at home | 1 | ^c,ii^3 / ^c,ii^1;  ^c,i^14 / ^c,i^9;  ^t,i^9 / ^t,i^6 | Folk methods were used (crushed garlic (bulbs and peduncles) mixed with feed) | Newcastle disease and avian flu; Ectoparasites | No |
| Ui_Tas_054 | June 2018 (24 days) /  Uigur district, Taskarasu village | 127 (^c^36, ^t^91) | ^ii^66 (^c^12, ^t^54);  ^i^61 (^c^24, ^t^37) | To commercialize their meat or consume it at home | 18 | ^c,i^9 / ^c,i^6;  ^t,ii^13 / ^t,ii^1;  ^t,i^17 / ^t,i^9 | Folk methods were used (crushed onion (bulbs and leaves) and garlic (bulbs and peduncles) mixed with feed) | Newcastle disease and avian flu; Ectoparasites and ascariasis | No |
| Ui_Tas_055 | June 2018 (25 days) /  Uigur district, Taskarasu village | 160 (^c^71, ^t^37, ^g^30, ^d^22) | ^ii^74 (^c^40, ^t^18, ^g^9, ^d^5);  ^i^86 (^c^31, ^t^19, ^g^21, ^d^17) | To sell them live to earn income according to needs | 23 | ^c,i^20 / ^c,i^12;  ^t,i^13 / ^t,i^9;  ^g,i^13 / ^g,i^5 | Appropriate antifungal drugs and antibiotics were used | Newcastle disease and avian flu; Ectoparasites and ascariasis | No |
| Tr_Zha_056 | June 2018 (19 days) /  Talgar district, Zhalkamys village | 36 (^c^27, ^g^9) | ^ii^10 (^c^9, ^g^1);  ^i^26 (^c^18, ^g^8) | To consume them at home | 1 | ^c,i^18 / ^c,i^7;  ^g,i^4 / ^g,i^2 | Folk methods were used (crushed onion (bulbs and leaves) and garlic (bulbs and peduncles) mixed with feed) | Ectoparasites | No |
| Ku_Zha_057 | June 2018 (25 days) /  Koksu district, Zharlyozek village | 158 (^c^99, ^t^59) | ^ii^64 (^c^41, ^t^23);  ^i^94 (^c^58, ^t^36) | To sell them live to earn income according to needs | 25 | ^c,ii^17 / ^c,ii^3;  ^c,i^14 / ^c,i^9;  ^t,ii^4 / ^t,ii^1;  ^t,i^20 / ^t,i^11 | Appropriate antifungal drugs and antibiotics were used | Newcastle disease and avian flu; Ectoparasites | No |
| Ag_Mat_058 | July 2018 (18 days) /  Agsu district, Matay village | 126 (^c^37, ^t^89) | ^ii^39 (^c^6, ^t^33);  ^i^87 (^c^31, ^t^56) | To commercialize their meat or consume it at home | 14 | ^c,i^16 / ^c,i^8;  ^t,i^19 / ^t,i^13 | Folk methods were used (crushed onion (bulbs and leaves) and garlic (bulbs and peduncles) mixed with feed) | Newcastle disease and avian flu; Ectoparasites and ascariasis | No |
| Ag_Egi_059 | July 2018 (7 days) /  Agsu district, Eginsu village | 93 (^c^74, ^g^8, ^d^11) | ^ii^49 (^c^46, ^g^1, ^d^2);  ^i^44 (^c^28, ^g^7, ^d^9) | To commercialize their eggs or consume it at home | 8 | ^c,ii^19 / ^c,ii^1;  ^c,i^8 / ^c,i^5 | Folk methods were used (crushed onion (bulbs and leaves) and garlic (bulbs and peduncles) mixed with feed) | Newcastle disease and avian flu; Ectoparasites and ascariasis | No |
| Kk_Alt_060 | July 2018 (10 days) /  Kerbulak district, Altynemel village | 48 (^c^42, ^g^6) | ^ii^13 (^c^12, ^g^1);  ^i^35 (^c^30, ^g^5) | To consume them at home | 1 | ^c,i^7 / ^c,i^4 | Folk methods were used (crushed onion (bulbs and leaves) and garlic (bulbs and peduncles) mixed with feed) | Newcastle disease and avian flu; Ectoparasites | No |
| Ra_Kai_061 | July 2018 (22 days) /  Raiymbek district, Kainar village | 52 (^c^37, ^g^15) | ^ii^14 (^c^12, ^g^2);  ^i^38 (^c^25, ^g^13) | To consume them at home | 1 | ^c,ii^4 / ^c,ii^1;  ^c,i^6 / ^c,i^5;  ^g,i^4 / ^g,i^2 | Folk methods were used (crushed red peppers mixed with feed) | Newcastle disease and avian flu; Ectoparasites and ascariasis | No |
| Kn_Bol_062 | July 2018 (23 days) /  Kegen district, Boleksaz village | 161 (^c^79, ^t^82) | ^ii^22 (^c^14, ^t^8);  ^i^139 (^c^65, ^t^74) | To sell them live to earn income according to needs | 10 | ^c,i^45 / ^c,i^28;  ^t,i^42 / ^t,i^19 | Folk methods were used (crushed onion (bulbs and leaves) and garlic (bulbs and peduncles) mixed with feed) | Newcastle disease and avian flu; Ectoparasites | No |
| Kt_Elt_063 | July 2018 (26 days) /  Karatal district, Eltai village | 151 (^c^68, ^t^60, ^g^23) | ^ii^62 (^c^24, ^t^29 ^g^9);  ^i^89 (^c^44, ^t^31, ^g^14) | To commercialize their meat or consume it at home | 8 | ^c,i^18 / ^c,i^13;  ^t,i^31 / ^t,i^24;  ^g,i^12 / ^g,i^9 | Folk methods were used (crushed onion (bulbs and leaves) and garlic (bulbs and peduncles) mixed with feed) | Newcastle disease and avian flu; Ectoparasites | No |
| Ba_Kuy_064 | July 2018 (10 days) /  Balkash district, Kuygan village | 118 (^c^102, ^g^16) | ^ii^64 (^c^61, ^g^3);  ^i^54 (^c^41, ^g^13) | To sell eggs | 18 | ^c,ii^19 / ^c,ii^4;  ^c,i^17 / ^c,i^14 | Folk methods were used (root of harmala; decoction) | Ectoparasites | No |
| Tn_Cit_065 | August 2018 (9 days) /  Taldykorgon city | ^c^13 | ^ii^3 (^c^3);  ^i^10 (^c^10) | To consume them at home | 1 | ^c,i^7 / ^c,i^4 | Folk methods were used (crushed onion (bulbs and leaves) and garlic (bulbs and peduncles) mixed with feed) | Newcastle disease and avian flu; Ectoparasites and ascariasis | No |
| Ks_Sha_066 | August 2018 (11 days) / Karasai district, Shamalgan village | 136 (^c^38, ^t^92, ^g^6) | ^ii^35 (^c^13, ^t^21, ^g^1);  ^i^101 (^c^25, ^t^71, ^g^5) | To commercialize their meat or consume it at home | 28 | ^t,i^28 / ^t,i^15 | Folk methods were used (crushed red peppers mixed with feed) | Newcastle disease and avian flu; Ectoparasites and ascariasis | No |
| Ks_Sha_067 | September 2018 (7 days) / Karasai district, Shamalgan village | 153 (^c^125, ^t^28) | ^ii^54 (^c^51, ^t^3);  ^i^99 (^c^74, ^t^25) | To commercialize their eggs or consume it at home | 32 | ^c,ii^22 / ^c,ii^3;  ^c,i^21 / ^c,i^8 | Folk methods were used (crushed onion (bulbs and leaves) and garlic (bulbs and peduncles) mixed with feed) | Newcastle disease and avian flu; Ectoparasites | No |
| Ks_Zha_068 | September 2018 (19 days) / Karasai district, Zhalpaksay village | 160 (^c^126, ^t^19, ^d^15) | ^ii^42 (^c^38, ^t^2, ^d^2);  ^i^118 (^c^88, ^t^17, ^d^13) | To sell them live to earn income according to needs | 29 | ^c,i^24 / ^c,i^20;  ^t,i^13 / ^t,i^11 | Folk methods were used (crushed onion (bulbs and leaves) and garlic (bulbs and peduncles) mixed with feed) | Newcastle disease and avian flu; Ectoparasites | No |
| En_Sar_069 | September 2018 (8 days) / Enbekshikazakh district, Sarybulak village | 46 (^c^31, ^d^15) | ^ii^6 (^c^4, ^d^2);  ^i^40 (^c^27, ^d^13) | To consume them at home | 1 | ^c,i^16 / ^c,i^10 | Folk methods were used (crushed onion (bulbs and leaves) and garlic (bulbs and peduncles) mixed with feed) | Newcastle disease and avian flu; Ectoparasites and ascariasis | No |
| Kk_Sha_070 | September 2018 (10 days) / Kerbulak district, Shanghanai village | 24 (^c^18, ^d^6) | ^ii^4 (^c^3, ^d^1);  ^i^20 (^c^15, ^d^5) | To consume them at home | 1 | ^c,i^10 / ^c,i^8 | Folk methods were used (crushed onion (bulbs and leaves) and garlic (bulbs and peduncles) mixed with feed) | Newcastle disease and avian flu; Ectoparasites | No |
| Kn_Zhy_071 | September 2018 (8 days) / Kegen district, Zhylysay village | 143 (^c^90, ^t^41, ^g^12) | ^ii^24 (^c^17, ^t^6, ^g^1);  ^i^119 (^c^73, ^t^35, ^g^11) | To commercialize their meat or consume it at home | 13 | ^t,i^21 / ^t,i^16 | Folk methods were used (small dose of children's urine, perorally) | Newcastle disease and avian flu; Ectoparasites and ascariasis | No |
| Il_Bay_072 | September 2018 (23 days) / Ile district, Bayserken village | 88 (^c^74, ^t^14) | ^ii^46 (^c^45, ^t^1);  ^i^42 (^c^29, ^t^13) | To commercialize their meat or consume it at home | 8 | ^c,ii^19 / ^c,ii^5;  ^c,i^13 / ^c,i^7;  ^t,i^8 / ^t,i^6 | Folk methods were used (crushed onion (bulbs and leaves) and garlic (bulbs and peduncles) mixed with feed) | Newcastle disease and avian flu; Ectoparasites | No |
| En_Kai_073 | September 2018 (12 days) / Enbekshikazakh district, Kainar village | 162 (^c^29, ^t^133) | ^ii^34 (^c^6, ^t^28);  ^i^128 (^c^23, ^t^105) | To commercialize their meat or consume it at home | 26 | ^t,ii^22 / ^t,ii^3;  ^t,i^36 / ^t,i^18 | Folk methods were used (crushed onion (bulbs and leaves) mixed with feed) | Newcastle disease and avian flu; Ectoparasites and ascariasis | No |
| Kk_Sar_074 | September 2018 (9 days) / Kerbulak district, Sarybastau village | 140 (^c^127, ^t^13) | ^ii^63 (^c^62, ^t^1);  ^i^77 (^c^65, ^t^12) | To sell eggs | 18 | ^c,ii^30 / ^c,ii^3;  ^c,i^12 / ^c,i^10 | Folk methods were used (crushed onion (bulbs and leaves) and garlic (bulbs and peduncles) mixed with feed) | Newcastle disease and avian flu; Ectoparasites | No |
| Al_Cit_075 | September 2018 (9 days) / Almaty city | ^c^9 | ^ii^9 | To consume them at home | 1 | ^c,ii^3 / ^c,ii^1 | Folk methods were used (crushed onion (bulbs and leaves) and garlic (bulbs and peduncles) mixed with feed) | Newcastle disease and avian flu; Ectoparasites and ascariasis | No |
| Ku_Kab_076 | September 2018 (11 days) / Koksu district, Kabylisa village | 150 (^c^137, ^t^8, ^g^5) | ^ii^74 (^c^72, ^t^1, ^g^1);  ^i^76 (^c^65, ^t^7, ^g^4) | To commercialize their eggs or consume it at home | 21 | ^c,ii^40 / ^c,ii^9;  ^c,i^14 / ^c,i^6 | Folk methods were used (crushed garlic (bulbs and peduncles) mixed with feed) | Newcastle disease and avian flu; Ectoparasites | No |
| Ui_Kyr_077 | September 2018 (10 days) / Uigur district, Kyrgyzsay village | 129 (^c^113, ^t^16) | ^ii^90 (^c^88, ^t^2);  ^i^39 (^c^25, ^t^14) | To sell eggs | 18 | ^c,ii^40 / ^c,ii^4 | Folk methods were used (crushed garlic (bulbs and peduncles) mixed with feed) | Ectoparasites | No |
| Ra_Bol_078 | September 2018 (9 days) / Raiymbek district, Boleksaz village | 25 (^c^17, ^g^3, ^d^5) | ^ii^9 (^c^7, ^g^1, ^d^1);  ^i^16 (^c^10, ^g^2, ^d^4) | To consume them at home | 1 | ^c,ii^1 / ^c,ii^1;  ^c,i^7 / ^c,i^3 | Folk methods were used (crushed onion (bulbs and leaves) and garlic (bulbs and peduncles) mixed with feed) | Newcastle disease and avian flu; Ectoparasites | No |
| Ag_Suy_079 | September 2018 (19 days) / Agsu district, Suyksay village | 53 (^c^39, ^g^14) | ^ii^9 (^c^6, ^g^3);  ^i^44 (^c^33, ^g^11) | To consume them at home | 3 | ^c,i^20 / ^c,i^7;  ^g,i^5 / ^g,i^1 | Folk methods were used (crushed onion (bulbs and leaves) mixed with feed) | Newcastle disease and avian flu; Ectoparasites and ascariasis | No |
| En_Tes_080 | September 2018 (21 days) / Enbekshikazakh district, Teskensu village | 142 (^c^59, ^t^83) | ^ii^100 (^c^34, ^t^66);  ^i^42 (^c^25, ^t^17) | To commercialize their meat or consume it at home | 16 | ^c,ii^21 / ^c,ii^3;  ^t,ii^23 / ^t,ii^2 | Folk methods were used (crushed red peppers mixed with feed) | Ectoparasites | No |
| Ba_Ber_081 | October 2018 (7 days) / Balkash district, Bereken village | 156 (^c^122, ^t^12, ^d^22) | ^ii^110 (^c^98, ^t^8, ^d^4);  ^i^46 (^c^24, ^t^4, ^d^18) | To commercialize their eggs or consume it at home | 16 | ^c,ii^40 / ^c,ii^7 | Folk methods were used (crushed onion (bulbs and leaves) and garlic (bulbs and peduncles) mixed with feed) | Newcastle disease and avian flu; Ectoparasites and ascariasis | No |
| Tn_Cit_082 | October 2018 (9 days) / Taldykorgon city | ^c^16 | ^ii^16 | To consume them at home | 1 | ^c,ii^8 / ^c,ii^2 | Folk methods were used (crushed onion (bulbs and leaves) and garlic (bulbs and peduncles) mixed with feed) | Newcastle disease and avian flu; Ectoparasites and ascariasis | No |
| Ag_Mat_083 | October 2018 (23 days) / Agsu district, Matay village | 149 (^c^109, ^t^40) | ^ii^128 (^c^88, ^t^40);  ^i^21 (^c^21) | To sell them live to earn income according to needs | 22 | ^c,ii^27 / ^c,ii^4;  ^c,i^14 / ^c,i^7;  ^t,ii^10 / ^t,ii^6 | Appropriate antifungal drugs and antibiotics were used | Ectoparasites | No |
| Tn_Cit_84 | October 2018 (11 days) / Taldykorgon city | ^c^12 | ^ii^3;  ^i^9 | To consume them at home | 1 | ^c,i^9 / ^c,i^6 | Folk methods were used (crushed garlic (bulbs and peduncles) mixed with feed) | Ectoparasites | No |
| En_Kar_085 | October 2018 (9 days) / Enbekshikazakh district, Karaturyk village | 144 (^c^113, ^t^31) | ^ii^91 (^c^77, ^t^14);  ^i^53 (^c^36, ^t^17) | To commercialize their eggs or consume it at home | 26 | ^c,ii^31 / ^c,ii^2 | Folk methods were used (crushed onion (bulbs and leaves) and garlic (bulbs and peduncles) mixed with feed) | Ectoparasites | No |
| Ks_Ayt_86 | October 2018 (9 days) / Karasai district, Aytey village | 24 (^c^11, ^t^13) | ^ii^24 | To consume them at home | 1 | ^t,ii^7 / ^t,ii^1 | Folk methods were used (small dose of children's urine, perorally) | Newcastle disease and avian flu; Ectoparasites and ascariasis | No |
| Ra_Sar_087 | October 2018 (12 days) / Raiymbek district, Saryzhaz village | 22 (^c^14, ^d^8) | ^ii^16 (^c^14, ^d^2);  ^i^6 (^d^6) | To consume them at home | 1 | ^c,ii^10 / ^c,ii^1 | Folk methods were used (crushed onion (bulbs and leaves) mixed with feed) | Newcastle disease and avian flu; Ectoparasites and ascariasis | No |
| Ks_Irg_088 | October 2018 (11 days) / Karasai district, Irgeli village | 16 (^c^13, ^g^3) | ^ii^16 | To consume them at home | 1 | ^c,ii^5 / ^c,ii^3 | Folk methods were used (crushed onion (bulbs and leaves) and garlic (bulbs and peduncles) mixed with feed) | Newcastle disease and avian flu; Ectoparasites and ascariasis | No |
| Ks_Ber_089 | October 2018 (9 days) / Karasai district, Bereke village | 161 (^c^33, ^t^128) | ^ii^161 | To commercialize their meat or consume it at home | 33 | ^t,ii^33 / ^t,ii^2 | Folk methods were used (crushed onion (bulbs and leaves) and garlic (bulbs and peduncles) mixed with feed) | Newcastle disease and avian flu; Ectoparasites and ascariasis | No |
| Ra_Bol_090 | October 2018 (10 days) / Raiymbek district, Boleksaz village | 143 (^c^127, ^g^16) | ^ii^143 | To commercialize their meat or consume it at home | 20 | ^c,ii^18 / ^c,ii^5 | Folk methods were used (crushed onion (bulbs and leaves) and garlic (bulbs and peduncles) mixed with feed) | Newcastle disease and avian flu; Ectoparasites and ascariasis | No |
| Ra_Sha_091 | October 2018 (19 days) /Raiymbek district, Shalkodin village | 156 (^c^83, ^t^26, ^g^25, ^d^22) | ^ii^73 (^c^58, ^t^5, ^g^6, ^d^4);  ^i^83 (^c^25, ^t^21, ^g^19, ^d^18) | To sell them live to earn income according to needs | 22 | ^c,ii^26 / ^c,ii^1;  ^c,i^9 / ^c,i^8;  ^t,i^17 / ^t,i^1;  ^g,i^7 / ^g,i^1 | Appropriate antifungal drugs and antibiotics were used | Ectoparasites | No |
| Ra_Sha_092 | October 2018 (11 days) / Raiymbek district, Shalkodin village | 27 (^c^19, ^t^8) | ^ii^27 | To consume them at home | 1 | ^c,ii^10 / ^c,ii^2 | Folk methods were used (crushed red peppers mixed with feed) | Ectoparasites | No |
| Ba_Top_093 | October 2018 (7 days) / Balkash district, Topar village | 99 (^c^90, ^g^9) | ^ii^67 (^c^58, ^g^9);  ^i^32 (^c^32) | To commercialize their eggs or consume it at home | 14 | ^c,ii^11 / ^c,ii^1;  ^c,i^14 / ^c,i^6 | Folk methods were used (crushed onion (bulbs and leaves) and garlic (bulbs and peduncles) mixed with feed) | Ectoparasites | No |
| Pa_Bas_094 | October 2018 (9 days) / Panfilov district, Baskunchin village | 18 (^c^14, ^d^4) | ^ii^18 | To consume them at home | 1 | ^c,ii^8 / ^c,ii^2 | Folk methods were used (root of harmala; infusion) | Newcastle disease and avian flu; Ectoparasites and ascariasis | No |
| Kn_Tas_095 | October 2018 (19 days) / Kegen district, Tasashin village | 157 (^c^48, ^t^109) | ^ii^74 (^c^31, ^t^43);  ^i^83 (^c^17, ^t^66) | To commercialize their meat or consume it at home | 22 | ^c,ii^17 / ^c,ii^1;  ^c,i^8 / ^c,i^5;  ^t,ii^14 / ^t,ii^3;  ^t,i^17 / ^t,i^13 | Folk methods were used (crushed onion (bulbs and leaves) mixed with feed) | Newcastle disease and avian flu; Ectoparasites and ascariasis | No |
| Tr_Bes_096 | October 2018 (9 days)/  Talgar district, Besagash village | 15 (^c^12, ^g^3) | ^ii^15 | To consume them at home | 1 | ^c,ii^6 / ^c,ii^1 | Folk methods were used (crushed onion (bulbs and leaves) and garlic (bulbs and peduncles) mixed with feed) | Newcastle disease and avian flu; Ectoparasites and ascariasis | No |
| Ks_Sha_097 | October 2018 (26 days) / Karasai district, Shamalgan village | 141 (^c^93, ^t^17, ^g^19, ^d^12) | ^ii^104 (^c^74, ^t^11, ^g^11, ^d^8);  ^i^37 (^c^19, ^t^6, ^g^8, ^d^4) | To commercialize their eggs or consume it at home | 21 | ^c,ii^10 / ^c,ii^3;  ^c,i^16 / ^c,i^14;  ^t,ii^6 / ^t,ii^5 | Folk methods were used (crushed onion (bulbs and leaves) and garlic (bulbs and peduncles) mixed with feed) | Newcastle disease and avian flu; Ectoparasites and ascariasis | No |
| Il_Bay_098 | October 2018 (22 days) / Ile district, Bayserken village | 152 (^c^71, ^t^54, ^g^27) | ^ii^152 | To sell them live to earn income according to needs | 17 | ^c,ii^21 / ^c,ii^6;  ^t,ii^18 / ^t,ii^2;  ^g,ii^10 / ^g,ii^1 | Folk methods were used (root of harmala; infusion) | Newcastle disease and avian flu; Ectoparasites and ascariasis | No |
| En_Bir_099 | November 2018 (24 days) / Enbekshikazakh district, Birlik village | 36 (^c^18, ^g^10, ^d^8) | ^ii^24 (^c^18, ^g^3, ^d^3);  ^i^12 (^g^7, ^d^5) | To consume them at home | 2 | ^c,ii^9 / ^c,ii^1;  ^g,i^7 / ^g,i^5 | Folk methods were used (crushed onion (bulbs and leaves) and garlic (bulbs and peduncles) mixed with feed) | Ectoparasites | No |
| Pa_Sar_0100 | November 2018 (9 days) / Panfilov district, Sarybel village | 133 (^c^106, ^d^27) | ^ii^133 | To commercialize their meat or consume it at home | 15 | ^c,ii^25 / ^c,ii^5 | Folk methods were used (crushed garlic (bulbs and peduncles) mixed with feed) | Ectoparasites | No |
| Ra_Sat_0101 | November 2018 (11 days) / Raiymbek district, Satin village | 157 (^c^126, ^t^21, ^g^5, ^d^5) | ^ii^157 | To commercialize their eggs or consume it at home | 18 | ^c,ii^46 / ^c,ii^11 | Folk methods were used (crushed red peppers mixed with feed) | Newcastle disease and avian flu; Ectoparasites and ascariasis | No |
| Kt_Esk_0102 | November 2018 (24 days) / Karatal district, Eskeldin village | 52 (^c^31, ^t^12, ^d^9) | ^ii^52 | To consume them at home | 5 | ^c,ii^18 / ^c,ii^2;  ^t,ii^7 / ^t,ii^1 | Folk methods were used (crushed onion (bulbs and leaves) and garlic (bulbs and peduncles) mixed with feed) | Ectoparasites | No |
| Tr_Ama_0103 | November 2018 (24 days) / Talgar district, Amangeldy village | 139 (^c^47, ^t^92) | ^ii^139 | To commercialize their meat or consume it at home | 31 | ^c,ii^13 / ^c,ii^3;  ^t,ii^32 / ^t,ii^7 | Folk methods were used (crushed onion (bulbs and leaves) mixed with feed) | Ectoparasites | No |
| Ku_Enb_0104 | November 2018 (19 days) / Koksu district, Enbekshin village | 78 (^c^55, ^g^23) | ^ii^69 (^c^46, ^g^23);  ^i^9 (^c^9) | To consume them at home | 6 | ^c,ii^15 / ^c,ii^2;  ^g,ii^6 / ^g,ii^1 | Folk methods were used (crushed onion (bulbs and leaves) mixed with feed) | Ectoparasites | No |
| Tr_Erk_0105 | November 2018 (7 days) / Talgar district, Erkin village | 66 (^c^43, ^t^19, ^d^4) | ^ii^66 | To consume them at home | 4 | ^c,ii^16 / ^c,ii^5 | Folk methods were used (crushed onion (bulbs and leaves) and garlic (bulbs and peduncles) mixed with feed) | Ectoparasites | No |
| Pa_Ulk_0106 | November 2018 (26 days) / Panfilov district, Ulkenagash village | 140 (^c^122, ^t^18) | ^ii^140 | To commercialize their eggs or consume it at home | 26 | ^c,ii^37 / ^c,ii^9;  ^t,ii^7 / ^t,ii^1 | Folk methods were used (root of harmala; decoction) | Newcastle disease and avian flu; Ectoparasites and ascariasis | No |
| Al_Cit_0107 | November 2018 (9 days) / Almaty city | ^c^15 | ^ii^15 | To consume them at home | 1 | ^c,ii^11 / ^c,ii^1 | Folk methods were used (crushed onion (bulbs and leaves) and garlic (bulbs and peduncles) mixed with feed) | Newcastle disease and avian flu; Ectoparasites and ascariasis | No |
| Kn_Bol_0108 | November 2018 (10 days) / Kegen district, Boleksaz village | 159 (^c^146, ^t^13) | ^ii^159 | To sell them live to earn income according to needs | 26 | ^c,ii^56 / ^c,ii^9 | Appropriate antifungal drugs and antibiotics were used | Ectoparasites | No |
| Ku_Ain_0109 | November 2018 (26 days) / Koksu district, Ainabulak village | 138 (^c^106, ^t^20, ^d^12) | ^ii^138 | To sell eggs | 16 | ^c,ii^57 / ^c,ii^11;  ^t,ii^5 / ^t,ii^2 | Folk methods were used (crushed onion (bulbs and leaves) and garlic (bulbs and peduncles) mixed with feed) | Ectoparasites | No |
| Al_Cit_0110 | November 2018 (8 days) / Almaty city | ^c^13 | ^ii^13 | To consume them at home | 1 | ^c,ii^6 / ^c,ii^1 | Folk methods were used (crushed red peppers mixed with feed) | Ectoparasites | No |
| En_Zha_0111 | November 2018 (25 days) / Enbekshikazakh district, Zhanashar village | 160 (^c^27, ^t^117, ^g^9, ^d^7) | ^ii^160 | To commercialize their meat or consume it at home | 29 | ^c,ii^12 / ^c,ii^3;  ^t,ii^51 / ^t,ii^15;  ^g,ii^3 / ^g,ii^1 | Folk methods were used (crushed onion (bulbs and leaves) and garlic (bulbs and peduncles) mixed with feed) | Ectoparasites | No |
| Ku_Zha_0112 | November 2018 (9 days) / Koksu district, Zharlyozek village | 143 (^c^131, ^t^12) | ^ii^143 | To commercialize their eggs or consume it at home | 26 | ^c,ii^25 / ^c,ii^14 | Folk methods were used (crushed garlic (bulbs and peduncles) mixed with feed) | Ectoparasites | No |
| Ra_Kai_0113 | November 2018 (11 days) / Raiymbek district, Kainar village | 62 (^c^41, ^t^21) | ^ii^62 | To consume them at home | 4 | ^c,ii^20 / ^c,ii^5 | Folk methods were used (crushed onion (bulbs and leaves) and garlic (bulbs and peduncles) mixed with feed) | Newcastle disease and avian flu; Ectoparasites and ascariasis | No |
| Kk_Zho_0114 | December 2018 (22 days) / Kerbulak district, Zholaman village | 93 (^c^62, ^t^24, ^g^7) | ^ii^93 | To sell them live to earn income according to needs | 12 | ^c,ii^34 / ^c,ii^9;  ^g,ii^5 / ^g,ii^1 | Folk methods were used (root of harmala; infusion) | Ectoparasites | No |
| Pa_Bir_0115 | December 2018 (22 days) / Panfilov district, Birlik village | 47 (^c^31, ^t^12, ^g^4) | ^ii^47 | To consume them at home | 3 | ^c,ii^14 / ^c,ii^3;  ^t,ii^9 / ^t,ii^2 | Folk methods were used (crushed onion (bulbs and leaves) and garlic (bulbs and peduncles) mixed with feed) | Newcastle disease and avian flu; Ectoparasites and ascariasis | No |
| Tr_Nur_0116 | December 2018 (17 days) / Talgar district, Nura village | 66 (^c^36, ^t^30) | ^ii^66 | To consume them at home | 5 | ^c,ii^20 / ^c,ii^6;  ^t,ii^21 / ^t,ii^3 | Folk methods were used (crushed onion (bulbs and leaves) mixed with feed) | Ectoparasites | No |
| Ag_Egi_0117 | December 2018 (9 days) / Agsu district, Eginsu village | 29 (^c^20, ^t^9) | ^ii^29 | To consume them at home | 1 | ^c,ii^13 / ^c,ii^1 | Folk methods were used (crushed onion (bulbs and leaves) mixed with feed) | Ectoparasites | No |
| Pa_Kon_0118 | December 2018 (22 days) / Panfilov district, Konyrolen village | 160 (^c^27, ^t^117, ^g^9, ^d^7) | ^ii^160 | To commercialize their eggs or consume it at home | 13 | ^c,ii^22 / ^c,ii^4;  ^t,ii^6 / ^t,ii^1;  ^g,ii^2 / ^g,ii^1 | Folk methods were used (crushed onion (bulbs and leaves) and garlic (bulbs and peduncles) mixed with feed) | Ectoparasites | No |
| Ag_Suy_0119 | December 2018 (8 days) / Agsu district, Suyksay village | 34 (^c^21, ^t^11, ^g^2) | ^ii^34 | To consume them at home | 1 | ^c,ii^9 / ^c,ii^2 | Folk methods were used (crushed garlic (bulbs and peduncles) mixed with feed) | Newcastle disease and avian flu; Ectoparasites and ascariasis | No |
| En_Bol_0120 | January 2019 (25 days) /  Enbekshikazakh district, Bolek village | 125 (^c^112, ^t^11, ^g^2) | ^ii^125 | To sell them live to earn income according to needs | 14 | ^c,ii^21 / ^c,ii^2;  ^t,ii^9 / ^t,ii^3 | Folk methods were used (crushed onion (bulbs and leaves) and garlic (bulbs and peduncles) mixed with feed) | Ectoparasites | No |
| En_Bay_0121 | January 2019 (11 days) /  Enbekshikazakh district, Bayseit village | 160 (^c^144, ^t^16) | ^ii^160 | To sell them on a regular basis | 38 | ^c,ii^43 / ^c,ii^11 | Appropriate antifungal drugs and antibiotics were used | Newcastle disease and avian flu; Ectoparasites and ascariasis | Yes (against Newcastle disease) |
| Al_Cit_0122 | January 2019 (9 days) / Almaty city | ^c^14 | ^ii^14 | To consume them at home | 1 | ^c,ii^7 / ^c,ii^1 | Folk methods were used (crushed onion (bulbs and leaves) mixed with feed) | Ectoparasites | No |
| Kk_Sha_0123 | February 2019 (25 days) / Kerbulak district, Shanghanai village | 129 (^c^109, ^t^17, ^g^3) | ^ii^129 | To commercialize their eggs or consume it at home | 24 | ^c,ii^29 / ^c,ii^8  ^t,ii^8 / ^t,ii^1 | Folk methods were used (crushed onion (bulbs and leaves) and garlic (bulbs and peduncles) mixed with feed) | Ectoparasites | No |
| Tn_Cit_0124 | February 2019 (8 days) / Taldykorgon city | ^c^17 | ^ii^17 | To consume them at home | 1 | ^c,ii^13 / ^c,ii^3 | Folk methods were used (crushed red peppers mixed with feed) | Ectoparasites | No |
| Ba_Kok_0125 | February 2019 (10 days) / Balkash district, Koktal village | 150 (^c^43, ^t^98, ^g^9) | ^ii^150 | To commercialize their meat or consume it at home | 32 | ^t,ii^40 / ^t,ii^9 | Folk methods were used (small dose of children's urine, perorally) | Ectoparasites | No |
| Ui_Uzy_0126 | February 2019 (24 days) / Uigur district, Uzyntam village | 36 (^c^24, ^g^12) | ^ii^36 | To consume them at home | 1 | ^c,ii^16 / ^c,ii^3;  ^g,ii^5 / ^g,ii^1 | Folk methods were used (crushed onion (bulbs and leaves) and garlic (bulbs and peduncles) mixed with feed) | Newcastle disease and avian flu; Ectoparasites and ascariasis | No |
| Kk_Alt_0127 | February 2019 (12 days) / Kerbulak district, Altynemel village | 140 (^c^122, ^g^18) | ^ii^140 | To commercialize their eggs or consume it at home | 27 | ^c,ii^43 / ^c,ii^14 | Folk methods were used (crushed onion (bulbs and leaves) and garlic (bulbs and peduncles) mixed with feed) | Ectoparasites | No |
| Il_Kar_0128 | March 2019 (11 days) / Ile district, Karaoy village | 50 (^c^26, ^g^13, ^d^11) | ^ii^50 | To consume them at home | 1 | ^c,ii^12 / ^c,ii^2 | Folk methods were used (crushed onion (bulbs and leaves) and garlic (bulbs and peduncles) mixed with feed) | Newcastle disease and avian flu; Ectoparasites and ascariasis | No |
| Ag_Egi_0129 | March 2019 (26 days) / Agsu district, Eginsu village | 161 (^c^139, ^t^22) | ^ii^161 | To commercialize their meat or consume it at home | 30 | ^c,ii^50 / ^c,ii^17;  ^t,ii^10 / ^t,ii^2 | Folk methods were used (crushed garlic (bulbs and peduncles) mixed with feed) | Ectoparasites | No |
| Ks_Zha_0130 | March 2019 (24 days) / Karasai district, Zhalpaksay village | 152 (^c^93, ^t^48, ^g^11) | ^ii^152 | To sell them live to earn income according to needs | 22 | ^c,ii^35 / ^c,ii^6;  ^t,ii^17 / ^t,ii^3 | Appropriate antifungal drugs and antibiotics were used | Ectoparasites | No |
| Tn_Cit_0131 | March 2019 (9 days) /  Taldykorgon city | ^c^16 | ^ii^16 | To consume them at home | 1 | ^c,ii^6 / ^c,ii^2 | Folk methods were used (crushed onion (bulbs and leaves) mixed with feed) | Ectoparasites | No |
| En_Koy_0132 | March 2019 (24 days) /  Enbekshikazakh district, Koyshibek village | 111 (^c^81, ^t^30) | ^ii^111 | To commercialize their eggs or consume it at home | 18 | ^c,ii^36 / ^c,ii^10;  ^t,ii^20 / ^t,ii^2 | Folk methods were used (crushed onion (bulbs and leaves) and garlic (bulbs and peduncles) mixed with feed) | Ectoparasites | No |
| Ag_Egi_0133 | March 2019 (12 days) / Agsu district, Eginsu village | 132 (^c^19, ^t^113) | ^ii^132 | To commercialize their meat or consume it at home | 27 | ^t,ii^52 / ^t,ii^23 | Folk methods were used (crushed onion (bulbs and leaves) and garlic (bulbs and peduncles) mixed with feed) | Newcastle disease and avian flu; Ectoparasites and ascariasis | No |
| Ks_Irg_0134 | March 2019 (9 days) /  Karasai district, Irgeli village | 79 (^c^65, ^d^14) | ^ii^79 | To sell eggs | 16 | ^c,ii^34 / ^c,ii^3 | Folk methods were used (crushed onion (bulbs and leaves) and garlic (bulbs and peduncles) mixed with feed) | Newcastle disease and avian flu; Ectoparasites and ascariasis | No |
| Ra_Bol_0135 | March 2019 (20 days) / Raiymbek district, Boleksaz village | 18 (^c^13, ^g^5) | ^ii^18 | To consume them at home | 1 | ^c,ii^12 / ^c,ii^1;  ^g,ii^4 / ^g,ii^1 | Folk methods were used (root of harmala; infusion) | Ectoparasites | No |
| Kt_Ayt_0136 | March 2019 (22 days) / Karatal district, Aytubiy village | 136 (^c^71, ^g^34, ^d^31) | ^ii^36 | To sell them live to earn income according to needs | 18 | ^c,ii^47 / ^c,ii^17;  ^g,ii^10 / ^g,ii^4 | Folk methods were used (crushed onion (bulbs and leaves) and garlic (bulbs and peduncles) mixed with feed) | Ectoparasites | No |
| Ui_Sho_0137 | March 2019 (9 days) /  Uigur district, Shoshanai village | 22 (^c^16, ^d^6) | ^ii^22 | To consume them at home | 1 | ^c,ii^6 / ^c,ii^1 | Folk methods were used (crushed onion (bulbs and leaves) and garlic (bulbs and peduncles) mixed with feed) | Ectoparasites | No |
| Pa_Bir_0138 | March 2019 (11 days) / Panfilov district, Birlik village | 82 (^c^62, ^t^20) | ^ii^82 | To commercialize their eggs or consume it at home | 16 | ^c,ii^30 / ^c,ii^8 | Folk methods were used (crushed onion (bulbs and leaves) mixed with feed) | Ectoparasites | No |
| Pa_Bas_0139 | March 2019 (22 days) / Panfilov district, Baskunchin village | 141 (^c^124, ^t^14, ^g^3) | ^ii^141 | To commercialize their eggs or consume it at home | 26 | ^c,ii^46 / ^c,ii^13;  ^t,ii^6 / ^t,ii^1 | Folk methods were used (crushed onion (bulbs and leaves) and garlic (bulbs and peduncles) mixed with feed) | Newcastle disease and avian flu; Ectoparasites and ascariasis | No |
| Il_Bor_0140 | March 2019 (10 days) / Ile district, Boraldai village | 21 (^c^16, ^g^5) | ^ii^21 | To consume them at home | 1 | ^c,ii^13 / ^c,ii^2 | Folk methods were used (crushed onion (bulbs and leaves) and garlic (bulbs and peduncles) mixed with feed) | Newcastle disease and avian flu; Ectoparasites and ascariasis | No |
| Pa_Kon_0141 | March 2019 (19 days)/  Panfilov district, Konyrolen village | 109 (^c^54, ^t^33, ^g^22) | ^ii^109 | To commercialize their meat or consume it at home | 26 | ^c,ii^20 / ^c,ii^6;  ^t,ii^22 / ^t,ii^4 | Folk methods were used (crushed garlic (bulbs and peduncles) mixed with feed) | Newcastle disease and avian flu; Ectoparasites and ascariasis | No |
| Kt_Tas_0142 | March 2019 (9 days) /  Karatal district, Tastobin village | 142 (^c^110, ^t^26, ^g^6) | ^ii^142 | To commercialize their eggs or consume it at home | 33 | ^c,ii^48 / ^c,ii^9 | Folk methods were used (crushed onion (bulbs and leaves) and garlic (bulb and peduncles) mixed with feed) | Newcastle disease and avian flu; Ectoparasites and ascariasis | No |
| Tr_Ama_0143 | March 2019 (23 days) / Talgar district, Amangeldy village | 38 (^c^29, ^t^9) | ^ii^38 | To consume them at home | 2 | ^c,ii^10 / ^c,ii^3;  ^t,ii^5 / ^t,ii^1 | Folk methods were used (crushed onion (bulbs and leaves) and garlic (bulbs and peduncles) mixed with feed) | Ectoparasites | No |
| En_Zha_0144 | March 2019 (7 days) /  Enbekshikazakh district, Zhanashar village | 132 (^c^22, ^t^110) | ^ii^132 | To commercialize their meat or consume it at home | 26 | ^t,ii^32 / ^t,ii^6 | Folk methods were used (crushed onion (bulbs and leaves) mixed with feed) | Ectoparasites | No |
| Kt_Esk_0145 | March 2019 (12 days) / Karatal district, Eskeldin village | 88 (^c^79, ^g^9) | ^ii^88 | To sell eggs | 28 | ^c,ii^48 / ^c,ii^5 | Folk methods were used (crushed onion (bulbs and leaves) and garlic (bulbs and peduncles) mixed with feed) | Ectoparasites | No |
| Kn_Bol_0146 | March 2019 (26 days) / Kegen district, Boleksaz village | 105 (^c^80, ^t^25) | ^ii^105 | To commercialize their eggs or consume it at home | 22 | ^c,ii^34 / ^c,ii^13;  ^t,ii^12 / ^t,ii^4 | Folk methods were used (crushed red peppers mixed with feed) | Ectoparasites | No |
| Pa_Ulk_0147 | March 2019 (8 days) /  Panfilov district, Ulkenagash village | 16 (^c^13, ^t^3) | ^ii^16 | To consume them at home | 1 | ^c,ii^6 / ^c,ii^1 | Folk methods were used (crushed onion (bulbs and leaves) and garlic (bulbs and peduncles) mixed with feed) | Ectoparasites | No |
| Ra_Bol_0148 | March 2019 (23 days) / Raiymbek district, Boleksaz village | 127 (^c^42, ^t^54, ^g^31) | ^ii^127 | To commercialize their meat or consume it at home | 18 | ^c,ii^28 / ^c,ii^2;  ^t,ii^18 / ^t,ii^4;  ^g,ii^21 / ^g,ii^3 | Folk methods were used (crushed onion (bulbs and leaves) and garlic (bulbs and peduncles) mixed with feed) | Newcastle disease and avian flu; Ectoparasites and ascariasis | No |
| En_Kyz_0149 | April 2019 (24 days) /  Enbekshikazakh district, Kyzylsharyk village | 86 (^c^23, ^t^63) | ^ii^86 | To commercialize their meat or consume it at home | 14 | ^c,ii^12 / ^c,ii^1;  ^t,ii^23 / ^t,ii^3 | Folk methods were used (crushed onion (bulbs and leaves) mixed with feed) | Newcastle disease and avian flu; Ectoparasites and ascariasis | No |
| Ag_Oyt_0150 | April 2019 (10 days) /  Agsu district, Oytogan village | 154 (^c^131, ^t^23) | ^ii^154 | To commercialize their meat or consume it at home | 22 | ^c,ii^42 / ^c,ii^9 | Folk methods were used (crushed onion (bulbs and leaves) and garlic (bulbs and peduncles) mixed with feed) | Ectoparasites | No |
| Ku_Alg_0151 | April 2019 (21 days) /  Koksu district, Algabas village | 21 (^c^17, ^t^4) | ^ii^21 | To consume them at home | 1 | ^c,ii^14 / ^c,ii^2;  ^t,ii^3 / ^t,ii^1 | Folk methods were used (crushed onion (bulbs and leaves) and garlic (bulbs and peduncles) mixed with feed) | Ectoparasites | No |
| En_Sar_0152 | April 2019 (23 days) /  Enbekshikazakh district, Kyzylsharyk village | 120 (^c^80, ^t^27, ^g^9, ^d^4) | ^ii^120 | To sell them live to earn income according to needs |  | ^c,ii^36 / ^c,ii^14;  ^t,ii^15 / ^t,ii^2 | Appropriate antifungal drugs and antibiotics were used | Ectoparasites | No |
| Ra_Keg_0153 | April 2019 (18 days) /  Raiymbek district, Kegen village | 116 (^c^78, ^t^22, ^g^10, ^d^6) | ^ii^116 | To commercialize their meat or consume it at home | 18 | ^c,ii^35 / ^c,ii^5;  ^t,ii^17 / ^t,ii^6 | Folk methods were used (crushed onion (bulbs and leaves) mixed with feed) | Ectoparasites and ascariasis | No |
| Ks_Ber_0154 | April 2019 (11 days) /  Karasai district, Bereke village | 96 (^c^82, ^t^14) | ^ii^96 | To commercialize their eggs or consume it at home | 16 | ^c,ii^25 / ^c,ii^7 | Folk methods were used (crushed onion (bulbs and leaves) and garlic (bulbs and peduncles) mixed with feed) | Ectoparasites and ascariasis | No |
| Ag_Egi_0155 | April 2019 (7 days) /  Agsu district, Eginsu village | 151 (^c^129, ^t^22) | ^ii^110 (^c^88, ^t^22);  ^i^41 (^c^41) | To sell eggs | 24 | ^c,ii^36 / ^c,ii^10 | Folk methods were used (crushed onion (bulbs and leaves) and garlic (bulbs and peduncles) mixed with feed) | Ectoparasites | No |
| Al_Cit_0156 | April 2019 (10 days) /  Almaty city | ^c^14 | ^ii^14 | To consume them at home | 1 | ^c,ii^8 / ^c,ii^2 | Folk methods were used (crushed onion (bulbs and leaves) mixed with feed) | Ectoparasites | No |
| En_Sar_0157 | April 2019 (22 days) /  Enbekshikazakh district, Sarybulak village | 157 (^c^77, ^t^80) | ^ii^157 | To sell them on a regular basis | 30 | ^c,ii^27 / ^c,ii^13;  ^t,ii^48 / ^t,ii^9 | Appropriate antifungal drugs and antibiotics were used | Newcastle disease and avian flu; Ectoparasites and ascariasis | Yes (against Newcastle disease) |
| Tr_Dos_0158 | April 2019 (9 days) /  Talgar district, Dostyk village | 123 (^c^95, ^t^28) | ^ii^123 | To sell eggs | 19 | ^c,ii^26 / ^c,ii^6 | Folk methods were used (crushed onion (bulbs and leaves) and garlic (bulbs and peduncles) mixed with feed) | Ectoparasites | No |
| Ks_Ayt_0159 | April 2019 (11 days) /  Karasai district, Aytey village | 146 (^c^105, ^t^41) | ^ii^146 | To commercialize their meat or consume it at home | 26 | ^c,ii^35 / ^c,ii^11 | Folk methods were used (crushed garlic (bulbs and peduncles) mixed with feed) | Ectoparasites | No |
| Ra_Kai_0160 | April 2019 (24 days) /  Raiymbek district, Kainar village | 122 (^c^59, ^t^63) | ^ii^122 | To commercialize their eggs or consume it at home | 22 | ^c,ii^33 / ^c,ii^4;  ^t,ii^23 / ^t,ii^3 | Folk methods were used (crushed onion (bulbs and leaves) and garlic (bulbs and peduncles) mixed with feed) | Newcastle disease and avian flu; Ectoparasites and ascariasis | No |
| Ui_Uzy_0161 | April 2019 (23 days) /  Uigur district, Uzyntam village | 148 (^c^61, ^t^87) | ^ii^123 (^c^36, ^t^87);  ^i^25 (^c^25) | To commercialize their meat or consume it at home | 16 | ^c,ii^25 / ^c,ii^7;  ^c,i^22 / ^c,i^10;  ^t,ii^40 / ^t,ii^4 | Folk methods were used (crushed onion (bulbs and leaves) and garlic (bulbs and peduncles) mixed with feed) | Ectoparasites | No |
| En_Bir_0162 | April 2019 (10 days) /  Enbekshikazakh district, Birlik village | 136 (^c^24, ^d^112) | ^ii^136 | To sell them live to earn income according to needs | 22 | ^c,ii^7 / ^c,ii^2 | Folk methods were used (crushed onion (bulbs and leaves) mixed with feed) | Newcastle disease and avian flu; Ectoparasites and ascariasis | No |
| Il_Zhe_0163 | April 2019 (11 days) /  Ile district, Zhetygen village | 22 (^c^18, ^g^4) | ^ii^22 | To consume them at home | 1 | ^c,ii^9 / ^c,ii^1 | Folk methods were used (crushed onion (bulbs and leaves) and garlic (bulbs and peduncles) mixed with feed) | Ectoparasites | No |
| Ui_Cha_0164 | April 2019 (12 days) /  Uigur district, Charyn village | 91 (^c^83, ^t^8) | ^ii^91 | To sell eggs | 18 | ^c,ii^29 / ^c,ii^3 | Folk methods were used (crushed onion (bulbs and leaves) and garlic (bulbs and peduncles) mixed with feed) | Ectoparasites | No |
| Tr_Ama_0165 | April 2019 (16 days) /  Talgar district, Amangeldy village | 139 (^c^94, ^t^32, ^g^13) | ^ii^106 (^c^61, ^t^32, ^g^13);  ^i^33 (^c^33) | To commercialize their meat or consume it at home | 26 | ^c,ii^17 / ^c,ii^2;  ^c,i^31 / ^c,i^24;  ^t,ii^17 / ^t,ii^1 | Folk methods were used (crushed onion (bulbs and leaves) and garlic (bulbs and peduncles) mixed with feed) | Ectoparasites | No |
| Ra_Aul_0166 | May 2019 (12 days) /  Raiymbek district, Boleksaz village | 147 (^c^52, ^t^95) | ^ii^147 | To commercialize their eggs or consume it at home | 22 | ^t,ii^39 / ^t,ii^6 | Folk methods were used (crushed onion (bulbs and leaves) and garlic (bulbs and peduncles) mixed with feed) | Ectoparasites | No |
| Tr_Bes_0167 | May 2019 (20 days) /  Talgar district, Besagash village | 141 (^c^59, ^t^44, ^d^38) | ^ii^141 | To sell eggs | 16 | ^c,ii^35 / ^c,ii^9;  ^t,ii^22 / ^t,ii^3 | Folk methods were used (crushed onion (bulbs and leaves) mixed with feed) | Newcastle disease and avian flu; Ectoparasites and ascariasis | No |
| En_She_0168 | May 2019 (17 days) /  Enbekshikazakh district, Shelek village | 109 (^c^28, ^t^81) | ^ii^109 | To commercialize their meat or consume it at home | 28 | ^c,ii^13 / ^c,ii^2;  ^t,ii^32 / ^t,ii^9 | Folk methods were used (crushed onion (bulbs and leaves) and garlic (bulbs and peduncles) mixed with feed) | Ectoparasites | No |
| Ag_Mat_0169 | May 2019 (19 days) /  Agsu district, Matay village | 161 (^c^114, ^t^28, ^g^19) | ^ii^106 (^c^58, ^t^28, ^g^19);  ^i^56 (^c^56) | To sell them live to earn income according to needs | 26 | ^c,ii^23 / ^c,ii^1;  ^c,i^23 / ^c,i^9;  ^t,ii^9 / ^t,ii^1;  ^g,ii^4 / ^g,ii^1 | Folk methods were used (crushed garlic (bulbs and peduncles) mixed with feed) | Ectoparasites | No |
| En_Bay_0170 | May 2019 (22 days) /  Enbekshikazakh district, Bayseit village | 115 (^c^79, ^g^36) | ^ii^115 | To sell eggs | 30 | ^c,ii^40 / ^c,ii^9;  ^g,ii^19 / ^g,ii^3 | Folk methods were used (crushed onion (bulbs and leaves) and garlic (bulbs and peduncles) mixed with feed) | Ectoparasites | No |
| Ag_Suy_0171 | May 2019 (11 days) /  Agsu district, Suyksay village | 36 (^c^28, ^t^5, ^g^3) | ^ii^16 (^c^8, ^t^5, ^g^3);  ^i^20 (^c^20) | To consume them at home | 2 | ^c,ii^4 / ^c,ii^2;  ^c,i^20 / ^c,i^16 | Folk methods were used (crushed red peppers mixed with feed) | Ectoparasites | No |
| Kt_Kar_0172 | May 2019 (18 days) /  Karatal district, Karashengel village | 41 (^c^26, ^t^15) | ^ii^9 (^c^6, ^t^3);  ^i^32 (^c^20, ^t^12) | To consume them at home | 1 | ^c,ii^2 / ^c,ii^1;  ^c,i^18 / ^c,i^10;  ^t,i^9 / ^t,i^5 | Folk methods were used (crushed onion (bulbs and leaves) and garlic (bulbs and peduncles) mixed with feed) | Newcastle disease and avian flu; Ectoparasites and ascariasis | No |
| Pa_Ulk_0173 | May 2019 (21 days) /  Panfilov district, Ulkenagash village | 151 (^c^120, ^t^31) | ^ii^98 (^c^82, ^t^16);  ^i^53 (^c^38, ^t^15) | To commercialize their meat or consume it at home | 18 | ^c,i^30 / ^c,i^14;  ^t,i^14 / ^t,i^11 | Folk methods were used (crushed onion (bulbs and leaves) mixed with feed) | Newcastle disease and avian flu; Ectoparasites and ascariasis | No |
| En_Tes_0174 | May 2019 (19 days) /  Enbekshikazakh district, Teskensu village | 133 (^c^96, ^t^37) | ^ii^133 | To commercialize their meat or consume it at home | 26 | ^c,ii^54 / ^c,ii^14;  ^t,ii^24 / ^t,ii^6 | Folk methods were used (crushed onion (bulbs and leaves) and garlic (bulbs and peduncles) mixed with feed) | Ectoparasites | No |
| En_Kok_0175 | May 2019 (16 days) /  Enbekshikazakh district, Kokpek village | 95 (^c^56, ^t^39) | ^ii^41 (^c^24, ^t^17);  ^i^54 (^c^32, ^t^22) | To commercialize their meat or consume it at home | 22 | ^c,ii^7 / ^c,ii^1;  ^c,i^18 / ^c,i^7;  ^t,ii^5 / ^t,ii^1;  ^t,i^13 / ^t,i^8 | Folk methods were used (crushed red peppers mixed with feed) | Ectoparasites | No |
| Kk_Zho_0176 | May 2019 (23 days) /  Kerbulak district, Zholaman village | 149 (^c^75, ^t^48, ^g^26) | ^ii^41 (^c^21, ^t^16, ^g^5);  ^i^54 (^c^54, ^t^32, ^g^21) | To commercialize their meat or consume it at home | 18 | ^c,i^22 / ^c,i^15;  ^t,i^16 / ^t,i^10;  ^g,i^8 / ^g,i^6 | Folk methods were used (crushed onion (bulbs and leaves) and garlic (bulbs and peduncles) mixed with feed) | Ectoparasites | No |
| Ks_Ayt_0177 | May 2019 (10 days) /  Karasai district, Aytey village | 95 (^c^58, ^d^37) | ^ii^25 (^c^16, ^d^9);  ^i^70 (^c^42, ^d^28) | To commercialize their eggs or consume it at home | 16 | ^c,ii^6 / ^c,ii^3;  ^c,i^39 / ^c,i^16 | Folk methods were used (crushed onion (bulbs and leaves) and garlic (bulbs and peduncles) mixed with feed) | Newcastle disease and avian flu; Ectoparasites and ascariasis | No |
| Pa_Sar_0178 | June 2019 (16 days) /  Panfilov district, Sarybel village | 36 (^c^25, ^g^11) | ^ii^10 (^c^7, ^g^3);  ^i^26 (^c^18, ^g^8) | To consume them at home | 1 | ^c,ii^2 / ^c,ii^1;  ^c,i^14 / ^c,i^5;  ^g,i^7 / ^g,i^2 | Folk methods were used (crushed onion (bulbs and leaves) and garlic (bulbs and peduncles) mixed with feed) | Newcastle disease and avian flu;  Ectoparasites and ascariasis | No |
| Pa_Kon_0179 | June 2019 (18 days) /  Panfilov district, Konyrolen village | 134 (^c^47, ^t^87) | ^ii^42 (^c^9, ^t^33);  ^i^92 (^c^38, ^t^54) | To commercialize their meat or consume it at home | 20 | ^c,ii^3 /^c,ii^2;  ^c,i^22 / ^c,i^9;  ^t,ii^10 / ^t,ii^3;  ^t,i^23 / ^t,i^17 | Folk methods were used (crushed garlic (bulbs and peduncles) mixed with feed) | Newcastle disease and avian flu; Ectoparasites and ascariasis | No |
| Pa_Ulk_0180 | June 2019 (24 days) /  Panfilov district, Ulkenagash village | 152 (^c^83, ^t^37, ^g^32) | ^ii^52 (^c^31, ^t^12, ^g^9);  ^i^100 (^c^52, ^t^25, ^g^23) | To commercialize their meat or consume it at home | 22 | ^c,ii^23 / ^c,ii^1;  ^c,i^31 / ^c,i^13;  ^t,i^19 / ^t,i^14;  ^g,i^12 / ^g,i^8 | Folk methods were used (crushed onion (bulbs and leaves) and garlic (bulbs and peduncles) mixed with feed) | Ectoparasites | No |
| Ra_Sar_0181 | July 2019 (23 days) /  Raiymbek district, Saryzhaz village | 132 (^c^85, ^t^30, ^d^17) | ^ii^52 (^c^24, ^t^9, ^d^3);  ^i^96 (^c^61, ^t^21, ^d^14) | To sell them live to earn income according to needs | 18 | ^c,ii^11 / ^c,ii^3;  ^c,i^31 / ^c,i^15;  ^t,ii^3 / ^t,ii^1;  ^t,i^20 / ^t,i^12 | Appropriate antifungal drugs and antibiotics were used | Ectoparasites | No |
| Pa_Bas_0182 | July 2019 (19 days) /  Panfilov district, Baskunchin village | 60 (^c^43, ^g^17) | ^ii^12 (^c^9, ^g^3);  ^i^48 (^c^34, ^g^14) | To consume them at home | 1 | ^c,i^26 / ^c,i^9;  ^g,i^9 / ^g,i^5 | Folk methods were used (crushed onion (bulbs and leaves) mixed with feed) | Ectoparasites | No |
| En_Sar_0183 | July 2019 (22 days) /  Enbekshikazakh district, Sarybulak village | 112 (^c^66, ^t^18, ^g^17, ^d^11) | ^ii^33 (^c^21, ^t^3, ^g^5, ^d^4);  ^i^79 (^c^45, ^t^15, ^g^12, ^d^7) | To commercialize their meat or consume it at home | 16 | ^c,i^20 / ^c,i^11;  ^t,i^9 / ^t,i^6;  ^g,i^5 / ^g,i^2 | Folk methods were used (crushed onion (bulbs and leaves) and garlic (bulbs and peduncles) mixed with feed) | Newcastle disease and avian flu; Ectoparasites and ascariasis | No |

***TABLE S3* The number of healthy and affected (with clinical signs and died) chickens in interviewed households by age categories**

| **#** | **Total number of**  **chickens** | **Healthy chicken age category** (days) | | | | | | | | | |  | **Affected chicken age category** (with clinical signs / died) | | | | | | | | | |
| --- | --- | --- | --- | --- | --- | --- | --- | --- | --- | --- | --- | --- | --- | --- | --- | --- | --- | --- | --- | --- | --- | --- |
|  |  | **7** | **14** | **21** | **30** | **45** | **60** | **90** | **120** | **150** | **≤180** |  | **7** | **14** | **21** | **30** | **45** | **60** | **90** | **120** | **150** | **≤180** |
| 1 | 14 (14) |  |  |  |  |  |  |  |  |  | 14 |  |  |  |  |  |  |  |  |  |  | 4 / 1 |
| 2 | 15 (15) |  |  |  |  |  |  |  |  |  | 15 |  |  |  |  |  |  |  |  |  |  | 4 / 2 |
| 3 | 13 (13) |  |  |  |  |  |  |  |  |  | 13 |  |  |  |  |  |  |  |  |  |  | 5 / 1 |
| 4 | 84 (56) |  |  |  |  |  |  |  |  |  | 56 |  |  |  |  |  |  |  |  |  |  | 9 / 3 |
| 5 | 152 (59) |  |  |  |  |  |  |  |  |  | 59 |  |  |  |  |  |  |  |  |  |  | 10 / 2 |
| 6 | 47 (35) |  |  |  |  |  |  |  |  |  | 35 |  |  |  |  |  |  |  |  |  |  | 10 / 1 |
| 7 | 68 (36) |  |  |  |  |  |  |  |  |  | 36 |  |  |  |  |  |  |  |  |  |  | 12 / 1 |
| 8 | 93 (42) |  |  |  |  |  |  |  |  |  | 42 |  |  |  |  |  |  |  |  |  |  | 12 / 2 |
| 9 | 58 (21) |  |  |  |  |  |  |  |  |  | 21 |  |  |  |  |  |  |  |  |  |  | 14 / 1 |
| 10 | 142 (66) |  |  |  |  |  |  |  |  |  | 66 |  |  |  |  |  |  |  |  |  |  | 17 / 3 |
| 11 | 29 (18) |  |  |  |  |  |  |  |  |  | 18 |  |  |  |  |  |  |  |  |  |  | 6 / 1 |
| 12 | 162 (55) |  |  |  |  |  |  |  |  |  | 55 |  |  |  |  |  |  |  |  |  |  | 19 / 5 |
| 13 | 89 (57) |  |  |  |  |  |  |  |  |  | 57 |  |  |  |  |  |  |  |  |  |  | 19 / 3 |
| 14 | 114 (66) |  |  |  |  |  |  |  |  |  | 66 |  |  |  |  |  |  |  |  |  |  | 26 / 17 |
| 15 | 32 (24) |  |  |  |  |  |  |  |  |  | 24 |  |  |  |  |  |  |  |  |  |  | 5 / 1 |
| 16 | 94 (23) |  |  |  |  |  |  |  |  |  | 23 |  |  |  |  |  |  |  |  |  |  |  |
| 17 | 104 (88) |  |  |  |  |  |  |  |  |  | 88 |  |  |  |  |  |  |  |  |  |  | 27 / 4 |
| 18 | 48 (35) | 11 | 17 |  |  |  |  |  |  |  | 7 |  | 14 / 6 | 12 / 5 |  |  |  |  |  |  |  |  |
| 19 | 147 (136) | 49 | 38 |  |  |  |  |  |  |  | 49 |  | 25 / 17 |  |  |  |  |  |  |  |  | 9 / 3 |
| 20 | 129 (113) | 63 | 24 | 15 |  |  |  |  |  |  | 11 |  | 21 / 18 | 14 / 9 | 16 / 12 |  |  |  |  |  |  |  |
| 21 | 153 (91) |  |  |  |  |  |  |  |  |  | 91 |  |  |  |  |  |  |  |  |  |  |  |
| 22 | 86 (80) |  |  |  |  |  |  |  |  |  | 80 |  |  |  |  |  |  |  |  |  |  | 21 / 4 |
| 23 | 54 (36) | 5 | 22 |  |  |  |  |  |  |  | 9 |  | 14 / 8 |  |  |  |  |  |  |  |  |  |
| 24 | 139 (52) | 14 | 9 | 13 |  |  |  |  |  |  | 16 |  | 5 / 3 | 8 / 6 | 4 / 1 |  |  |  |  |  |  | 6 / 1 |
| 25 | 44 (41) |  |  |  |  |  |  |  |  |  | 41 |  |  |  |  |  |  |  |  |  |  | 15 / 3 |
| 26 | 53 (47) | 16 | 17 | 8 |  |  |  |  |  |  | 6 |  | 3 / 3 | 5 / 5 | 3 / 3 |  |  |  |  |  |  |  |
| 27 | 71 (65) | 7 | 12 | 19 | 10 |  |  |  |  |  | 17 |  | 9 / 6 | 7 / 6 | 9 / 3 |  |  |  |  |  |  | 11 / 3 |
| 28 | 25 (22) |  |  |  |  |  |  |  |  |  | 22 |  |  |  |  |  |  |  |  |  |  | 6 / 2 |
| 29 | 33 (18) |  |  |  |  |  |  |  |  |  | 18 |  |  |  |  |  |  |  |  |  |  |  |
| 30 | 134 (66) |  |  |  |  |  |  |  |  |  | 66 |  |  |  |  |  |  |  |  |  |  | 26 / 6 |
| 31 | 88 (85) |  |  |  |  |  |  |  |  |  | 85 |  |  |  |  |  |  |  |  |  |  | 20 / 1 |
| 32 | 61 (44) |  |  | 20 | 13 |  |  |  |  |  | 11 |  |  | 8 / 8 | 7 / 4 |  |  |  |  |  |  |  |
| 33 | 94 (58) |  |  |  |  |  |  |  |  |  | 58 |  |  |  |  |  |  |  |  |  |  |  |
| 34 | 155 (101) | 13 | 26 |  | 16 |  |  |  |  |  | 46 |  |  | 12 / 9 |  | 10 / 7 |  |  |  |  |  |  |
| 35 | 153 (112) |  |  |  |  |  |  |  |  |  | 112 |  |  |  |  |  |  |  |  |  |  | 17 / 4 |
| 36 | 69 (62) |  |  |  |  |  |  |  |  |  | 62 |  |  |  |  |  |  |  |  |  |  | 26 / 5 |
| 37 | 12 (12) |  |  |  |  |  |  |  |  |  | 12 |  |  |  |  |  |  |  |  |  |  | 3 / 2 |
| 38 | 46 (35) |  |  |  |  |  |  |  |  |  | 35 |  |  |  |  |  |  |  |  |  |  | 15 / 1 |
| 39 | 133 (27) |  |  |  |  |  |  |  |  |  | 27 |  |  |  |  |  |  |  |  |  |  |  |
| 40 | 148 (52) |  |  |  |  |  |  |  |  |  | 52 |  |  |  |  |  |  |  |  |  |  | 21 / 4 |
| 41 | 29 (23) |  |  |  |  |  |  |  |  |  | 23 |  |  |  |  |  |  |  |  |  |  | 12 / 2 |
| 42 | 128 (22) |  |  |  |  |  |  |  |  |  | 22 |  |  |  |  |  |  |  |  |  |  |  |
| 43 | 149 (143) |  | 19 |  | 25 |  | 8 |  |  |  | 91 |  |  | 7 / 5 |  | 4 / 3 |  | 2 / 1 |  |  |  | 27 / 5 |
| 44 | 7 (7) |  |  |  |  |  |  |  |  |  | 7 |  |  |  |  |  |  |  |  |  |  | 4 / 1 |
| 45 | 45 (39) |  |  |  |  |  |  |  |  |  | 39 |  |  |  |  |  |  |  |  |  |  | 21 / 2 |
| 46 | 93 (18) |  |  |  |  |  |  |  |  |  | 18 |  |  |  |  |  |  |  |  |  |  | 6 / 1 |
| 47 | 65 (44) |  |  | 31 |  |  |  |  |  |  | 13 |  |  |  | 9 / 5 |  |  |  |  |  |  | 8 / 3 |
| 48 | 115 (104) |  |  |  |  |  |  |  |  |  | 104 |  |  |  |  |  |  |  |  |  |  | 35 / 8 |
| 49 | 148 (43) |  |  |  |  |  |  |  |  |  | 43 |  |  |  |  |  |  |  |  |  |  | 12 / 3 |
| 50 | 137 (124) |  |  | 12 | 17 | 13 | 19 |  |  |  | 63 |  |  |  | 9 / 8 | 4 / 2 | 5 / 2 | 2 / 1 |  |  |  | 18 / 1 |
| 51 | 69 (48) |  | 11 |  | 12 |  | 12 |  |  |  | 23 |  |  | 6 / 5 |  | 4 / 2 |  | 3 / 1 |  |  |  | 5 / 1 |
| 52 | 133 (124) |  |  |  |  | 58 |  |  |  |  | 66 |  |  |  |  |  | 20 / 11 |  |  |  |  | 16 / 2 |
| 53 | 52 (37) |  |  |  | 8 |  |  | 14 |  |  | 15 |  |  |  |  | 8 / 7 |  |  | 6 / 2 |  |  | 3 / 1 |
| 54 | 127 (36) |  |  |  |  |  | 24 |  |  |  | 12 |  |  |  |  |  |  | 9 / 6 |  |  |  |  |
| 55 | 160 (71) |  |  | 10 |  | 14 |  | 7 |  |  | 40 |  |  |  | 10 / 7 |  | 4 / 4 |  | 6 / 1 |  |  |  |
| 56 | 36 (27) |  |  |  |  |  |  | 18 |  |  | 9 |  |  |  |  |  |  |  | 18 / 7 |  |  |  |
| 57 | 158 (99) |  | 10 |  | 15 |  | 33 |  |  |  | 41 |  |  | 6 / 5 |  | 3 / 3 |  | 5 / 1 |  |  |  | 17 / 3 |
| 58 | 126 (37) | 8 |  |  |  | 10 |  | 13 |  |  | 6 |  | 8 / 3 |  |  |  | 5 / 3 |  | 3 / 2 |  |  |  |
| 59 | 93 (74) |  |  |  | 28 |  |  |  |  |  | 46 |  |  |  |  | 8 / 5 |  |  |  |  |  | 19 / 1 |
| 60 | 48 (42) |  |  |  |  |  | 11 |  | 19 |  | 12 |  |  |  |  |  |  |  | 7 / 4 |  |  |  |
| 61 | 52 (37) |  |  |  |  |  |  | 25 |  |  | 12 |  |  |  |  |  |  |  | 6 / 5 |  |  | 4 / 1 |
| 62 | 161 (79) |  |  |  | 16 |  | 19 |  | 30 |  | 14 |  |  |  |  | 16 / 9 |  | 20 / 8 |  | 9 / 2 |  |  |
| 63 | 151 (68) |  |  | 10 |  |  |  |  | 18 | 16 | 24 |  |  |  | 10 / 10 |  |  |  |  | 5 / 2 | 3 / 1 |  |
| 64 | 118 (102) |  |  |  |  | 19 |  |  |  | 22 | 61 |  |  |  |  |  | 12 / 11 |  |  |  | 5 / 3 | 19 / 4 |
| 65 | 13 (13) |  |  |  |  |  |  | 10 |  |  | 3 |  |  |  |  |  |  |  | 7 / 4 |  |  |  |
| 66 | 136 (38) |  |  |  |  |  |  |  | 25 |  | 13 |  |  |  |  |  |  |  |  |  |  |  |
| 67 | 153 (125) |  |  |  |  |  |  |  | 32 | 42 | 51 |  |  |  |  |  |  |  |  | 17 / 7 | 4 / 1 | 22 / 3 |
| 68 | 160 (126) |  |  |  |  | 19 |  | 26 | 21 | 22 | 38 |  |  |  |  |  |  |  | 15 / 14 |  | 9 / 6 |  |
| 69 | 46 (31) |  |  |  |  |  |  |  | 27 |  | 4 |  |  |  |  |  |  |  |  | 16 / 10 |  |  |
| 70 | 24 (18) |  |  |  |  |  | 15 |  |  |  | 3 |  |  |  |  |  |  | 10 / 8 |  |  |  |  |
| 71 | 143 (90) |  |  |  |  |  | 9 | 31 | 33 |  | 17 |  |  |  |  |  |  |  |  |  |  |  |
| 72 | 88 (74) |  |  |  |  |  |  |  | 13 | 16 | 45 |  |  |  |  |  |  |  |  | 13 / 7 |  | 19 / 5 |
| 73 | 162 (29) |  |  |  |  |  |  |  | 23 |  | 6 |  |  |  |  |  |  |  |  |  |  |  |
| 74 | 140 (127) |  |  |  |  |  |  | 19 | 22 | 24 | 62 |  |  |  |  |  |  |  | 12 / 10 |  |  | 30 / 3 |
| 75 | 9 (9) |  |  |  |  |  |  |  |  |  | 9 |  |  |  |  |  |  |  |  |  |  | 3 / 1 |
| 76 | 150 (137) |  |  |  |  |  |  | 20 | 34 | 11 | 72 |  |  |  |  |  |  |  | 14 / 6 |  |  | 40 / 9 |
| 77 | 129 (113) |  |  |  |  |  |  |  | 25 |  | 88 |  |  |  |  |  |  |  |  |  |  | 40 / 4 |
| 78 | 25 (17) |  |  |  |  |  |  |  |  | 10 | 7 |  |  |  |  |  |  |  |  |  | 7 / 3 | 1 / 1 |
| 79 | 53 (39) |  |  |  |  |  |  |  | 23 | 10 | 6 |  |  |  |  |  |  |  |  | 15 / 6 | 5 / 1 |  |
| 80 | 142 (59) |  |  |  |  |  |  |  |  | 25 | 34 |  |  |  |  |  |  |  |  |  |  | 21 / 3 |
| 81 | 156 (122) |  |  |  |  |  |  |  | 13 | 11 | 98 |  |  |  |  |  |  |  |  |  |  | 40 / 7 |
| 82 | 16 (16) |  |  |  |  |  |  |  |  |  | 16 |  |  |  |  |  |  |  |  |  |  | 8 / 2 |
| 83 | 149 (109) |  |  |  |  |  |  |  | 8 | 13 | 88 |  |  |  |  |  |  |  |  | 8 / 5 | 6 / 2 | 27 / 4 |
| 84 | 12 (12) |  |  |  |  |  |  |  | 9 |  | 3 |  |  |  |  |  |  |  |  | 9 / 6 |  |  |
| 85 | 144 (113) |  |  |  |  |  |  |  | 12 | 24 | 77 |  |  |  |  |  |  |  |  |  |  | 31 / 2 |
| 86 | 24 (11) |  |  |  |  |  |  |  |  |  | 11 |  |  |  |  |  |  |  |  |  |  |  |
| 87 | 22 (14) |  |  |  |  |  |  |  |  |  | 14 |  |  |  |  |  |  |  |  |  |  | 10 / 1 |
| 88 | 16 (13) |  |  |  |  |  |  |  |  |  | 13 |  |  |  |  |  |  |  |  |  |  | 5 / 3 |
| 89 | 161 (33) |  |  |  |  |  |  |  |  |  | 33 |  |  |  |  |  |  |  |  |  |  |  |
| 90 | 143 (127) |  |  |  |  |  |  |  |  |  | 127 |  |  |  |  |  |  |  |  |  |  | 18 / 5 |
| 91 | 156 (83) |  |  |  |  |  |  |  |  | 25 | 58 |  |  |  |  |  |  |  |  |  | 9 / 8 | 26 / 1 |
| 92 | 27 (19) |  |  |  |  |  |  |  |  |  | 19 |  |  |  |  |  |  |  |  |  |  | 10 / 2 |
| 93 | 99 (90) |  |  |  |  |  |  |  |  | 32 | 58 |  |  |  |  |  |  |  |  |  | 14 / 6 | 11 / 1 |
| 94 | 18 (14) |  |  |  |  |  |  |  |  |  | 14 |  |  |  |  |  |  |  |  |  |  | 8 / 2 |
| 95 | 157 (48) |  |  |  |  |  |  |  |  | 17 | 31 |  |  |  |  |  |  |  |  |  | 8 / 5 | 17 / 1 |
| 96 | 15 (12) |  |  |  |  |  |  |  |  |  | 12 |  |  |  |  |  |  |  |  |  |  | 6 / 1 |
| 97 | 141 (93) |  |  |  |  |  |  |  |  | 19 | 74 |  |  |  |  |  |  |  |  |  | 16 / 14 | 10 / 3 |
| 98 | 152 (71) |  |  |  |  |  |  |  |  |  | 71 |  |  |  |  |  |  |  |  |  |  | 21 / 6 |
| 99 | 36 (18) |  |  |  |  |  |  |  |  |  | 18 |  |  |  |  |  |  |  |  |  |  | 9 / 1 |
| 100 | 133 (106) |  |  |  |  |  |  |  |  |  | 106 |  |  |  |  |  |  |  |  |  |  | 25 / 5 |
| 101 | 157 (126) |  |  |  |  |  |  |  |  |  | 126 |  |  |  |  |  |  |  |  |  |  | 46 / 11 |
| 102 | 52 (31) |  |  |  |  |  |  |  |  |  | 31 |  |  |  |  |  |  |  |  |  |  | 18 / 2 |
| 103 | 139 (47) |  |  |  |  |  |  |  |  |  | 47 |  |  |  |  |  |  |  |  |  |  | 13 / 3 |
| 104 | 78 (55) |  |  |  |  |  |  |  |  | 9 | 46 |  |  |  |  |  |  |  |  |  |  | 15 / 2 |
| 105 | 66 (43) |  |  |  |  |  |  |  |  |  | 43 |  |  |  |  |  |  |  |  |  |  | 16 / 5 |
| 106 | 140 (122) |  |  |  |  |  |  |  |  |  | 122 |  |  |  |  |  |  |  |  |  |  | 37 / 9 |
| 107 | 15 (15) |  |  |  |  |  |  |  |  |  | 15 |  |  |  |  |  |  |  |  |  |  | 11 / 1 |
| 108 | 159 (146) |  |  |  |  |  |  |  |  |  | 146 |  |  |  |  |  |  |  |  |  |  | 56 / 9 |
| 109 | 138 (106) |  |  |  |  |  |  |  |  |  | 106 |  |  |  |  |  |  |  |  |  |  | 57 / 11 |
| 110 | 13 (13) |  |  |  |  |  |  |  |  |  | 13 |  |  |  |  |  |  |  |  |  |  | 6 / 1 |
| 111 | 160 (27) |  |  |  |  |  |  |  |  |  | 27 |  |  |  |  |  |  |  |  |  |  | 12 / 3 |
| 112 | 143 (131) |  |  |  |  |  |  |  |  |  | 131 |  |  |  |  |  |  |  |  |  |  | 25 / 14 |
| 113 | 62 (41) |  |  |  |  |  |  |  |  |  | 41 |  |  |  |  |  |  |  |  |  |  | 20 / 5 |
| 114 | 93 (62) |  |  |  |  |  |  |  |  |  | 62 |  |  |  |  |  |  |  |  |  |  | 34 / 9 |
| 115 | 47 (31) |  |  |  |  |  |  |  |  |  | 31 |  |  |  |  |  |  |  |  |  |  | 14 / 3 |
| 116 | 66 (36) |  |  |  |  |  |  |  |  |  | 36 |  |  |  |  |  |  |  |  |  |  | 20 / 6 |
| 117 | 29 (20) |  |  |  |  |  |  |  |  |  | 20 |  |  |  |  |  |  |  |  |  |  | 13 / 1 |
| 118 | 160 (27) |  |  |  |  |  |  |  |  |  | 27 |  |  |  |  |  |  |  |  |  |  | 22 / 4 |
| 119 | 34 (21) |  |  |  |  |  |  |  |  |  | 21 |  |  |  |  |  |  |  |  |  |  | 9 / 2 |
| 120 | 125 (112) |  |  |  |  |  |  |  |  |  | 112 |  |  |  |  |  |  |  |  |  |  | 21 / 2 |
| 121 | 160 (144) |  |  |  |  |  |  |  |  |  | 144 |  |  |  |  |  |  |  |  |  |  | 43 / 11 |
| 122 | 14 (14) |  |  |  |  |  |  |  |  |  | 14 |  |  |  |  |  |  |  |  |  |  | 7 / 1 |
| 123 | 129 (109) |  |  |  |  |  |  |  |  |  | 109 |  |  |  |  |  |  |  |  |  |  | 29 / 8 |
| 124 | 17 (17) |  |  |  |  |  |  |  |  |  | 17 |  |  |  |  |  |  |  |  |  |  | 13 / 3 |
| 125 | 150 (43) |  |  |  |  |  |  |  |  |  | 43 |  |  |  |  |  |  |  |  |  |  |  |
| 126 | 36 (24) |  |  |  |  |  |  |  |  |  | 24 |  |  |  |  |  |  |  |  |  |  | 16 / 3 |
| 127 | 140 (122) |  |  |  |  |  |  |  |  |  | 122 |  |  |  |  |  |  |  |  |  |  | 43 / 14 |
| 128 | 50 (26) |  |  |  |  |  |  |  |  |  | 26 |  |  |  |  |  |  |  |  |  |  | 2 / 12 |
| 129 | 161 (139) |  |  |  |  |  |  |  |  |  | 139 |  |  |  |  |  |  |  |  |  |  | 50 / 17 |
| 130 | 152 (93) |  |  |  |  |  |  |  |  |  | 93 |  |  |  |  |  |  |  |  |  |  | 35 / 6 |
| 131 | 16 (16) |  |  |  |  |  |  |  |  |  | 16 |  |  |  |  |  |  |  |  |  |  | 6 / 2 |
| 132 | 111 (81) |  |  |  |  |  |  |  |  |  | 81 |  |  |  |  |  |  |  |  |  |  | 36 / 10 |
| 133 | 132 (19) |  |  |  |  |  |  |  |  |  | 19 |  |  |  |  |  |  |  |  |  |  |  |
| 134 | 79 (65) |  |  |  |  |  |  |  |  |  | 65 |  |  |  |  |  |  |  |  |  |  | 34 / 3 |
| 135 | 18 (13) |  |  |  |  |  |  |  |  |  | 13 |  |  |  |  |  |  |  |  |  |  | 12 / 1 |
| 136 | 136 (71) |  |  |  |  |  |  |  |  |  | 71 |  |  |  |  |  |  |  |  |  |  | 47 / 17 |
| 137 | 22 (16) |  |  |  |  |  |  |  |  |  | 16 |  |  |  |  |  |  |  |  |  |  | 6 / 1 |
| 138 | 82 (62) |  |  |  |  |  |  |  |  |  | 62 |  |  |  |  |  |  |  |  |  |  | 30 / 8 |
| 139 | 141 (124) |  |  |  |  |  |  |  |  |  | 124 |  |  |  |  |  |  |  |  |  |  | 46 / 13 |
| 140 | 21 (16) |  |  |  |  |  |  |  |  |  | 16 |  |  |  |  |  |  |  |  |  |  | 13 / 2 |
| 141 | 109 (54) |  |  |  |  |  |  |  |  |  | 54 |  |  |  |  |  |  |  |  |  |  | 20 / 6 |
| 142 | 142 (110) |  |  |  |  |  |  |  |  |  | 110 |  |  |  |  |  |  |  |  |  |  | 48 / 9 |
| 143 | 38 (29) |  |  |  |  |  |  |  |  |  | 29 |  |  |  |  |  |  |  |  |  |  | 10 / 3 |
| 144 | 132 (22) |  |  |  |  |  |  |  |  |  | 22 |  |  |  |  |  |  |  |  |  |  |  |
| 145 | 88 (79) |  |  |  |  |  |  |  |  |  | 79 |  |  |  |  |  |  |  |  |  |  | 48 / 5 |
| 146 | 105 (80) |  |  |  |  |  |  |  |  |  | 80 |  |  |  |  |  |  |  |  |  |  | 34 / 13 |
| 147 | 16 (13) |  |  |  |  |  |  |  |  |  | 13 |  |  |  |  |  |  |  |  |  |  | 6 / 1 |
| 148 | 127 (42) |  |  |  |  |  |  |  |  |  | 42 |  |  |  |  |  |  |  |  |  |  | 28 / 2 |
| 149 | 86 (23) |  |  |  |  |  |  |  |  |  | 23 |  |  |  |  |  |  |  |  |  |  | 12 / 1 |
| 150 | 154 (131) |  |  |  |  |  |  |  |  |  | 131 |  |  |  |  |  |  |  |  |  |  | 42 / 9 |
| 151 | 21 (17) |  |  |  |  |  |  |  |  |  | 17 |  |  |  |  |  |  |  |  |  |  | 14 / 2 |
| 152 | 120 (80) |  |  |  |  |  |  |  |  |  | 80 |  |  |  |  |  |  |  |  |  |  | 36 / 14 |
| 153 | 116 (78) |  |  |  |  |  |  |  |  |  | 78 |  |  |  |  |  |  |  |  |  |  | 35 / 5 |
| 154 | 96 (82) |  |  |  |  |  |  |  |  |  | 82 |  |  |  |  |  |  |  |  |  |  | 25 / 7 |
| 155 | 151 (129) | 19 | 12 | 10 |  |  |  |  |  |  | 88 |  |  |  |  |  |  |  |  |  |  | 36 / 10 |
| 156 | 14 (14) |  |  |  |  |  |  |  |  |  | 14 |  |  |  |  |  |  |  |  |  |  | 8 / 2 |
| 157 | 157 (77) |  |  |  |  |  |  |  |  |  | 77 |  |  |  |  |  |  |  |  |  |  | 27 / 13 |
| 158 | 123 (95) |  |  |  |  |  |  |  |  |  | 95 |  |  |  |  |  |  |  |  |  |  | 26 / 6 |
| 159 | 146 (105) |  |  |  |  |  |  |  |  |  | 105 |  |  |  |  |  |  |  |  |  |  | 35 / 11 |
| 160 | 122 (59) |  |  |  |  |  |  |  |  |  | 59 |  |  |  |  |  |  |  |  |  |  | 33 / 4 |
| 161 | 148 (61) | 14 |  | 11 |  |  |  |  |  |  | 36 |  | 14 / 8 |  | 8 / 2 |  |  |  |  |  |  | 25 / 7 |
| 162 | 136 (24) |  |  |  |  |  |  |  |  |  | 24 |  |  |  |  |  |  |  |  |  |  | 7 / 2 |
| 163 | 22 (18) |  |  |  |  |  |  |  |  |  | 18 |  |  |  |  |  |  |  |  |  |  | 9 / 1 |
| 164 | 91 (83) |  |  |  |  |  |  |  |  |  | 83 |  |  |  |  |  |  |  |  |  |  | 29 / 3 |
| 165 | 139 (94) |  | 20 | 13 |  |  |  |  |  |  | 61 |  |  | 20 / 17 | 11 / 7 |  |  |  |  |  |  | 17 / 2 |
| 166 | 147 (52) |  |  |  |  |  |  |  |  |  | 52 |  |  |  |  |  |  |  |  |  |  |  |
| 167 | 141 (59) |  |  |  |  |  |  |  |  |  | 59 |  |  |  |  |  |  |  |  |  |  | 35 / 9 |
| 168 | 109 (28) |  |  |  |  |  |  |  |  |  | 28 |  |  |  |  |  |  |  |  |  |  | 13 / 2 |
| 169 | 161 (114) |  | 56 |  |  |  |  |  |  |  | 58 |  |  | 23 / 9 |  |  |  |  |  |  |  | 23 / 1 |
| 170 | 115 (79) |  |  |  |  |  |  |  |  |  | 79 |  |  |  |  |  |  |  |  |  |  | 40 / 9 |
| 171 | 36 (28) | 12 | 8 |  |  |  |  |  |  |  | 8 |  | 12 / 8 | 8 / 8 |  |  |  |  |  |  |  | 4 / 2 |
| 172 | 41 (26) |  |  | 18 |  |  |  |  |  |  | 8 |  |  |  | 18 / 10 |  |  |  |  |  |  | 2 / 1 |
| 173 | 151 (120) | 11 | 14 | 13 |  |  |  |  |  |  | 82 |  | 11 / 7 | 12 / 5 | 7 / 2 |  |  |  |  |  |  |  |
| 174 | 133 (96) |  |  |  |  |  |  |  |  |  | 96 |  |  |  |  |  |  |  |  |  |  | 54 / 14 |
| 175 | 95 (56) |  | 32 |  |  |  |  |  |  |  | 24 |  |  | 18 / 7 |  |  |  |  |  |  |  | 7 / 1 |
| 176 | 149 (75) | 23 |  | 31 |  |  |  |  |  |  | 21 |  | 13 / 9 |  | 9 / 6 |  |  |  |  |  |  |  |
| 177 | 95 (58) |  |  |  | 42 |  |  |  |  |  | 16 |  |  |  |  | 39 / 16 |  |  |  |  |  | 6 / 3 |
| 178 | 36 (25) |  |  | 18 |  |  |  |  |  |  | 7 |  |  |  | 14 / 5 |  |  |  |  |  |  | 2 / 1 |
| 179 | 134 (47) |  |  |  | 16 | 22 |  |  |  |  | 9 |  |  |  |  | 13 / 7 | 9 / 2 |  |  |  |  | 3 / 2 |
| 180 | 152 (83) |  |  | 20 |  | 10 | 22 |  |  |  | 31 |  |  |  | 18 / 8 |  | 7 / 4 | 6 / 1 |  |  |  | 23 / 1 |
| 181 | 132 (85) |  |  |  | 28 |  |  | 33 |  |  | 24 |  |  |  |  | 19 / 12 |  |  | 12 / 3 |  |  | 11 / 3 |
| 182 | 60 (43) |  |  |  |  |  | 34 |  |  |  | 9 |  |  |  |  |  |  | 26 / 9 |  |  |  |  |
| 183 | 112 (66) |  |  |  |  |  | 29 | 16 |  |  | 21 |  |  |  |  |  |  | 20 / 11 |  |  |  |  |
| **Total** | | **265** | **347** | **272** | **246** | **165** | **235** | **232** | **387** | **348** | **8363** |  | **149 / 96** | **166 / 109** | **162 / 93** | **128 / 73** | **62 / 37** | **103 / 47** | **106 / 58** | **92 / 45** | **86 / 50** | **2845 / 634** |
| **All together** | | **10860** (2497 young; 8363adults) | | | | | | | | | |  | **3899 / 1242** | | | | | | | | | |

***TABLE S4* The number of healthy and affected (with clinical signs and died) turkeys in interviewed households by age categories**

| **#** | **Total number of**  **turkeys** | **Healthy turkey age category** (days) | | | | | | | | | |  | **Affected turkey age category** (with clinical signs / died) | | | | | | | | | |
| --- | --- | --- | --- | --- | --- | --- | --- | --- | --- | --- | --- | --- | --- | --- | --- | --- | --- | --- | --- | --- | --- | --- |
|  |  | **7** | **14** | **21** | **30** | **45** | **60** | **90** | **120** | **150** | **≤180** |  | **7** | **14** | **21** | **30** | **45** | **60** | **90** | **120** | **150** | **≤180** |
| 4 | 84 (28) |  |  |  |  |  |  |  |  |  | 28 |  |  |  |  |  |  |  |  |  |  | 9 / 2 |
| 5 | 152 (43) |  |  |  |  |  |  |  |  |  | 43 |  |  |  |  |  |  |  |  |  |  | 12 / 1 |
| 6 | 47 (12) |  |  |  |  |  |  |  |  |  | 12 |  |  |  |  |  |  |  |  |  |  |  |
| 7 | 68 (18) |  |  |  |  |  |  |  |  |  | 18 |  |  |  |  |  |  |  |  |  |  | 10 / 2 |
| 8 | 93 (34) |  |  |  |  |  |  |  |  |  | 34 |  |  |  |  |  |  |  |  |  |  | 8 / 3 |
| 9 | 58 (23) |  |  |  |  |  |  |  |  |  | 23 |  |  |  |  |  |  |  |  |  |  | 10 / 1 |
| 10 | 142 (45) |  |  |  |  |  |  |  |  |  | 45 |  |  |  |  |  |  |  |  |  |  |  |
| 11 | 29 (7) |  |  |  |  |  |  |  |  |  | 7 |  |  |  |  |  |  |  |  |  |  |  |
| 12 | 162 (107) |  |  |  |  |  |  |  |  |  | 107 |  |  |  |  |  |  |  |  |  |  | 19 / 2 |
| 13 | 89 (14) |  |  |  |  |  |  |  |  |  | 14 |  |  |  |  |  |  |  |  |  |  | 4 / 1 |
| 14 | 114 (31) |  |  |  |  |  |  |  |  |  | 31 |  |  |  |  |  |  |  |  |  |  | 18 / 2 |
| 15 | 32 (8) |  |  |  |  |  |  |  |  |  | 8 |  |  |  |  |  |  |  |  |  |  |  |
| 16 | 94 (71) |  |  |  |  |  |  |  |  |  | 71 |  |  |  |  |  |  |  |  |  |  | 25 / 7 |
| 17 | 104 (16) |  |  |  |  |  |  |  |  |  | 16 |  |  |  |  |  |  |  |  |  |  |  |
| 19 | 147 (11) |  |  |  |  |  |  |  |  |  | 11 |  |  |  |  |  |  |  |  |  |  |  |
| 21 | 153 (62) |  |  |  |  |  |  |  |  |  | 62 |  |  |  |  |  |  |  |  |  |  | 14 / 1 |
| 22 | 86 (6) |  |  |  |  |  |  |  |  |  | 6 |  |  |  |  |  |  |  |  |  |  |  |
| 23 | 54 (18) | 16 |  |  |  |  |  |  |  |  | 2 |  | 12 / 6 |  |  |  |  |  |  |  |  |  |
| 24 | 139 (87) |  |  |  |  |  |  |  |  |  | 87 |  |  |  |  |  |  |  |  |  |  |  |
| 26 | 53 (4) |  |  |  |  |  |  |  |  |  | 4 |  |  |  |  |  |  |  |  |  |  |  |
| 27 | 71 (6) |  |  |  |  |  |  |  |  |  | 6 |  |  |  |  |  |  |  |  |  |  |  |
| 29 | 33 (15) |  |  |  |  |  |  |  |  |  | 15 |  |  |  |  |  |  |  |  |  |  | 8 / 1 |
| 30 | 134 (58) |  |  |  |  |  |  |  |  |  | 58 |  |  |  |  |  |  |  |  |  |  | 15 / 1 |
| 32 | 61 (8) |  |  |  |  |  |  |  |  |  | 8 |  |  |  |  |  |  |  |  |  |  |  |
| 33 | 94 (36) |  |  |  |  |  |  |  |  |  | 36 |  |  |  |  |  |  |  |  |  |  | 14 / 11 |
| 34 | 155 (31) |  |  |  |  |  |  |  |  |  | 31 |  |  |  |  |  |  |  |  |  |  |  |
| 35 | 153 (41) |  |  |  |  |  |  |  |  |  | 41 |  |  |  |  |  |  |  |  |  |  | 10 / 2 |
| 36 | 69 (7) |  |  |  |  |  |  |  |  |  | 7 |  |  |  |  |  |  |  |  |  |  |  |
| 39 | 133 (106) |  |  |  |  |  |  |  |  |  | 106 |  |  |  |  |  |  |  |  |  |  | 30 / 5 |
| 40 | 148 (63) |  |  |  |  |  |  |  |  |  | 63 |  |  |  |  |  |  |  |  |  |  | 14 / 3 |
| 41 | 29 (6) |  |  |  |  |  |  |  |  |  | 6 |  |  |  |  |  |  |  |  |  |  |  |
| 42 | 128 (106) |  |  |  |  |  |  |  |  |  | 106 |  |  |  |  |  |  |  |  |  |  | 18 / 2 |
| 43 | 149 (6) |  |  |  |  |  |  |  |  |  | 6 |  |  |  |  |  |  |  |  |  |  |  |
| 45 | 45 (6) |  |  |  |  |  |  |  |  |  | 6 |  |  |  |  |  |  |  |  |  |  |  |
| 46 | 93 (66) |  |  |  |  |  |  |  |  |  | 66 |  |  |  |  |  |  |  |  |  |  | 25 / 3 |
| 48 | 115 (11) |  |  |  |  |  |  |  |  |  | 11 |  |  |  |  |  |  |  |  |  |  |  |
| 49 | 148 (83) |  |  |  |  |  |  |  |  |  | 83 |  |  |  |  |  |  |  |  |  |  | 20 / 6 |
| 52 | 133 (6) |  |  |  |  |  |  |  |  |  | 6 |  |  |  |  |  |  |  |  |  |  |  |
| 53 | 52 (15) |  |  |  |  | 13 |  |  |  |  | 2 |  |  |  |  |  | 9 / 6 |  |  |  |  |  |
| 54 | 127 (91) |  |  |  | 18 | 19 |  |  |  |  | 54 |  |  |  |  | 9 / 6 | 8 / 3 |  |  |  |  | 13 / 1 |
| 55 | 160 (37) |  |  |  |  | 10 | 9 |  |  |  | 18 |  |  |  |  |  | 9 / 7 | 4 / 2 |  |  |  |  |
| 57 | 158 (59) |  | 6 | 4 | 17 |  | 9 |  |  |  | 23 |  |  | 4 / 3 | 4 / 2 | 9 / 5 |  | 3 / 1 |  |  |  | 4 / 1 |
| 58 | 126 (89) |  |  |  |  | 24 | 18 | 14 |  |  | 33 |  |  |  |  |  | 15 / 10 | 4 / 3 |  |  |  |  |
| 62 | 161 (82) |  |  |  |  |  | 31 | 43 |  |  | 8 |  |  |  |  |  |  | 30 / 17 | 12 / 2 |  |  |  |
| 63 | 151 (60) |  |  |  |  |  | 9 |  | 22 |  | 29 |  |  |  |  |  |  | 9 / 9 |  | 22 / 15 |  |  |
| 66 | 136 (92) |  |  |  |  |  |  | 24 | 47 |  | 21 |  |  |  |  |  |  |  |  | 28 / 15 |  |  |
| 67 | 153 (28) |  |  |  |  |  |  |  | 25 |  | 3 |  |  |  |  |  |  |  |  |  |  |  |
| 68 | 160 (19) |  |  |  |  |  |  | 17 |  |  | 2 |  |  |  |  |  |  |  | 13 / 11 |  |  |  |
| 71 | 143 (41) |  |  |  |  |  |  | 21 | 14 |  | 6 |  |  |  |  |  |  |  | 12 / 10 | 9 / 6 |  |  |
| 72 | 88 (14) |  |  |  |  |  |  |  | 13 |  | 1 |  |  |  |  |  |  |  |  | 8 / 6 |  |  |
| 73 | 162 (133) |  |  |  |  |  |  | 27 | 46 | 32 | 28 |  |  |  |  |  |  |  | 19 / 8 | 9 / 5 | 8 / 5 | 22 / 3 |
| 74 | 140 (13) |  |  |  |  |  |  |  | 12 |  | 1 |  |  |  |  |  |  |  |  |  |  |  |
| 76 | 150 (8) |  |  |  |  |  |  |  |  | 7 | 1 |  |  |  |  |  |  |  |  |  |  |  |
| 77 | 129 (16) |  |  |  |  |  |  |  | 14 |  | 2 |  |  |  |  |  |  |  |  |  |  |  |
| 80 | 142 (83) |  |  |  |  |  |  |  |  | 17 | 66 |  |  |  |  |  |  |  |  |  |  | 23 / 2 |
| 81 | 156 (12) |  |  |  |  |  |  |  |  | 4 | 8 |  |  |  |  |  |  |  |  |  |  |  |
| 83 | 149 (40) |  |  |  |  |  |  |  |  |  | 40 |  |  |  |  |  |  |  |  |  |  | 10 / 6 |
| 85 | 144 (31) |  |  |  |  |  |  |  |  | 17 | 14 |  |  |  |  |  |  |  |  |  |  |  |
| 86 | 24 (13) |  |  |  |  |  |  |  |  |  | 13 |  |  |  |  |  |  |  |  |  |  | 7 / 1 |
| 89 | 161 (128) |  |  |  |  |  |  |  |  |  | 128 |  |  |  |  |  |  |  |  |  |  | 33 / 2 |
| 91 | 156 (26) |  |  |  |  |  |  |  | 21 |  | 5 |  |  |  |  |  |  |  |  | 17 / 1 |  |  |
| 92 | 27 (8) |  |  |  |  |  |  |  |  |  | 8 |  |  |  |  |  |  |  |  |  |  |  |
| 95 | 157 (109) |  |  |  |  |  |  |  |  | 66 | 43 |  |  |  |  |  |  |  |  |  | 17 / 13 | 14 / 3 |
| 97 | 141 (17) |  |  |  |  |  |  |  |  | 6 | 11 |  |  |  |  |  |  |  |  |  | 6 / 5 |  |
| 98 | 152 (54) |  |  |  |  |  |  |  |  |  | 54 |  |  |  |  |  |  |  |  |  |  | 18 / 2 |
| 101 | 157 (21) |  |  |  |  |  |  |  |  |  | 21 |  |  |  |  |  |  |  |  |  |  |  |
| 102 | 52 (12) |  |  |  |  |  |  |  |  |  | 12 |  |  |  |  |  |  |  |  |  |  | 7 / 1 |
| 103 | 139 (92) |  |  |  |  |  |  |  |  |  | 92 |  |  |  |  |  |  |  |  |  |  | 32 / 7 |
| 105 | 66 (19) |  |  |  |  |  |  |  |  |  | 19 |  |  |  |  |  |  |  |  |  |  |  |
| 106 | 140 (18) |  |  |  |  |  |  |  |  |  | 18 |  |  |  |  |  |  |  |  |  |  | 7 / 1 |
| 108 | 159 (13) |  |  |  |  |  |  |  |  |  | 13 |  |  |  |  |  |  |  |  |  |  |  |
| 109 | 138 (20) |  |  |  |  |  |  |  |  |  | 20 |  |  |  |  |  |  |  |  |  |  | 5 / 2 |
| 111 | 160 (117) |  |  |  |  |  |  |  |  |  | 117 |  |  |  |  |  |  |  |  |  |  | 51 / 15 |
| 112 | 143 (12) |  |  |  |  |  |  |  |  |  | 12 |  |  |  |  |  |  |  |  |  |  |  |
| 113 | 62 (21) |  |  |  |  |  |  |  |  |  | 21 |  |  |  |  |  |  |  |  |  |  |  |
| 114 | 93 (24) |  |  |  |  |  |  |  |  |  | 24 |  |  |  |  |  |  |  |  |  |  |  |
| 115 | 47 (12) |  |  |  |  |  |  |  |  |  | 12 |  |  |  |  |  |  |  |  |  |  | 9 / 2 |
| 116 | 66 (30) |  |  |  |  |  |  |  |  |  | 30 |  |  |  |  |  |  |  |  |  |  | 21 / 3 |
| 117 | 29 (9) |  |  |  |  |  |  |  |  |  | 9 |  |  |  |  |  |  |  |  |  |  |  |
| 118 | 160 (117) |  |  |  |  |  |  |  |  |  | 117 |  |  |  |  |  |  |  |  |  |  | 6 / 1 |
| 119 | 34 (11) |  |  |  |  |  |  |  |  |  | 11 |  |  |  |  |  |  |  |  |  |  |  |
| 120 | 125 (11) |  |  |  |  |  |  |  |  |  | 11 |  |  |  |  |  |  |  |  |  |  | 9 / 3 |
| 121 | 160 (16) |  |  |  |  |  |  |  |  |  | 16 |  |  |  |  |  |  |  |  |  |  |  |
| 123 | 129 (17) |  |  |  |  |  |  |  |  |  | 17 |  |  |  |  |  |  |  |  |  |  | 8 / 1 |
| 125 | 150 (98) |  |  |  |  |  |  |  |  |  | 98 |  |  |  |  |  |  |  |  |  |  | 40 / 9 |
| 129 | 161 (22) |  |  |  |  |  |  |  |  |  | 22 |  |  |  |  |  |  |  |  |  |  | 10 / 2 |
| 130 | 152 (48) |  |  |  |  |  |  |  |  |  | 48 |  |  |  |  |  |  |  |  |  |  | 17 / 3 |
| 132 | 111 (30) |  |  |  |  |  |  |  |  |  | 30 |  |  |  |  |  |  |  |  |  |  | 20 / 2 |
| 133 | 132 (113) |  |  |  |  |  |  |  |  |  | 113 |  |  |  |  |  |  |  |  |  |  | 52 / 23 |
| 138 | 82 (20) |  |  |  |  |  |  |  |  |  | 20 |  |  |  |  |  |  |  |  |  |  |  |
| 139 | 141 (14) |  |  |  |  |  |  |  |  |  | 14 |  |  |  |  |  |  |  |  |  |  | 6 / 1 |
| 141 | 109 (33) |  |  |  |  |  |  |  |  |  | 33 |  |  |  |  |  |  |  |  |  |  | 22 / 4 |
| 142 | 142 (26) |  |  |  |  |  |  |  |  |  | 26 |  |  |  |  |  |  |  |  |  |  |  |
| 143 | 38 (9) |  |  |  |  |  |  |  |  |  | 9 |  |  |  |  |  |  |  |  |  |  | 5 / 1 |
| 144 | 132 (110) |  |  |  |  |  |  |  |  |  | 110 |  |  |  |  |  |  |  |  |  |  | 32 / 6 |
| 146 | 105 (25) |  |  |  |  |  |  |  |  |  | 25 |  |  |  |  |  |  |  |  |  |  | 12 / 4 |
| 147 | 16 (3) |  |  |  |  |  |  |  |  |  | 3 |  |  |  |  |  |  |  |  |  |  |  |
| 148 | 127 (54) |  |  |  |  |  |  |  |  |  | 54 |  |  |  |  |  |  |  |  |  |  | 18 / 4 |
| 149 | 86 (63) |  |  |  |  |  |  |  |  |  | 63 |  |  |  |  |  |  |  |  |  |  | 23 / 3 |
| 150 | 154 (23) |  |  |  |  |  |  |  |  |  | 23 |  |  |  |  |  |  |  |  |  |  |  |
| 151 | 21 (4) |  |  |  |  |  |  |  |  |  | 4 |  |  |  |  |  |  |  |  |  |  | 3 / 1 |
| 152 | 120 (27) |  |  |  |  |  |  |  |  |  | 27 |  |  |  |  |  |  |  |  |  |  | 15 / 2 |
| 153 | 116 (22) |  |  |  |  |  |  |  |  |  | 22 |  |  |  |  |  |  |  |  |  |  | 17 / 6 |
| 154 | 96 (14) |  |  |  |  |  |  |  |  |  | 14 |  |  |  |  |  |  |  |  |  |  |  |
| 155 | 151 (22) |  |  |  |  |  |  |  |  |  | 22 |  |  |  |  |  |  |  |  |  |  |  |
| 157 | 157 (80) |  |  |  |  |  |  |  |  |  | 80 |  |  |  |  |  |  |  |  |  |  | 48 / 9 |
| 158 | 123 (28) |  |  |  |  |  |  |  |  |  | 28 |  |  |  |  |  |  |  |  |  |  |  |
| 159 | 146 (41) |  |  |  |  |  |  |  |  |  | 41 |  |  |  |  |  |  |  |  |  |  |  |
| 160 | 122 (63) |  |  |  |  |  |  |  |  |  | 63 |  |  |  |  |  |  |  |  |  |  | 23 / 3 |
| 161 | 148 (87) |  |  |  |  |  |  |  |  |  | 87 |  |  |  |  |  |  |  |  |  |  | 40 / 4 |
| 164 | 91 (8) |  |  |  |  |  |  |  |  |  | 8 |  |  |  |  |  |  |  |  |  |  |  |
| 165 | 139 (32) |  |  |  |  |  |  |  |  |  | 32 |  |  |  |  |  |  |  |  |  |  | 17 / 1 |
| 166 | 147 (95) |  |  |  |  |  |  |  |  |  | 95 |  |  |  |  |  |  |  |  |  |  | 39 / 6 |
| 167 | 141 (44) |  |  |  |  |  |  |  |  |  | 44 |  |  |  |  |  |  |  |  |  |  | 22 / 3 |
| 168 | 109 (81) |  |  |  |  |  |  |  |  |  | 81 |  |  |  |  |  |  |  |  |  |  | 32 / 9 |
| 169 | 161 (28) |  |  |  |  |  |  |  |  |  | 28 |  |  |  |  |  |  |  |  |  |  | 9 / 1 |
| 171 | 36 (5) |  |  |  |  |  |  |  |  |  | 5 |  |  |  |  |  |  |  |  |  |  |  |
| 172 | 41 (15) |  | 12 |  |  |  |  |  |  |  | 3 |  |  | 9 / 5 |  |  |  |  |  |  |  |  |
| 173 | 151 (31) | 15 |  |  |  |  |  |  |  |  | 16 |  | 14 / 11 |  |  |  |  |  |  |  |  |  |
| 174 | 133 (37) |  |  |  |  |  |  |  |  |  | 37 |  |  |  |  |  |  |  |  |  |  | 24 / 6 |
| 175 | 95 (39) |  |  | 22 |  |  |  |  |  |  | 17 |  |  |  | 13 / 8 |  |  |  |  |  |  | 5 / 1 |
| 176 | 149 (48) |  |  | 14 | 18 |  |  |  |  |  | 16 |  |  |  |  | 9 / 7 | 7 / 3 |  |  |  |  |  |
| 179 | 134 (87) |  |  |  | 29 | 25 |  |  |  |  | 33 |  |  |  |  |  | 23 / 17 |  |  |  |  | 10 / 3 |
| 180 | 152 (37) |  |  |  |  | 9 | 16 |  |  |  | 12 |  |  |  |  |  | 9 / 8 | 10 / 6 |  |  |  |  |
| 181 | 132 (30) |  |  |  |  |  | 21 |  |  |  | 9 |  |  |  |  |  |  | 20 / 12 |  |  |  | 3 / 1 |
| 183 | 112 (18) |  |  |  |  |  |  | 15 |  |  | 3 |  |  |  |  |  |  |  | 9 / 6 |  |  |  |
| **Total** | | **31** | **18** | **40** | **82** | **100** | **113** | **161** | **214** | **149** | **4022** |  | **26 / 17** | **13 / 8** | **17 / 10** | **27 / 18** | **80 / 54** | **80 / 50** | **65 / 37** | **93 / 48** | **31 / 23** | **1146 / 231** |
| **All together** | | **4930** (908 young; 4022 adults) | | | | | | | | | |  | **1578 / 496** | | | | | | | | | |

***TABLE S5* The number of healthy and affected (with clinical signs and died) geese in interviewed households by age categories**

| **#** | **Total number of**  **geese** | **Healthy goose age category** (days) | | | | | | | | | |  | **Affected goose age category** (with clinical signs / died) | | | | | | | | | |
| --- | --- | --- | --- | --- | --- | --- | --- | --- | --- | --- | --- | --- | --- | --- | --- | --- | --- | --- | --- | --- | --- | --- |
|  |  | **7** | **14** | **21** | **30** | **45** | **60** | **90** | **120** | **150** | **≤180** |  | **7** | **14** | **21** | **30** | **45** | **60** | **90** | **120** | **150** | **≤180** |
| 5 | 152 (23) |  |  |  |  |  |  |  |  |  | 23 |  |  |  |  |  |  |  |  |  |  |  |
| 7 | 68 (14) |  |  |  |  |  |  |  |  |  | 14 |  |  |  |  |  |  |  |  |  |  |  |
| 8 | 93 (17) |  |  |  |  |  |  |  |  |  | 17 |  |  |  |  |  |  |  |  |  |  | 4 / 1 |
| 10 | 142 (19) |  |  |  |  |  |  |  |  |  | 19 |  |  |  |  |  |  |  |  |  |  |  |
| 11 | 29 (4) |  |  |  |  |  |  |  |  |  | 4 |  |  |  |  |  |  |  |  |  |  |  |
| 13 | 89 (18) |  |  |  |  |  |  |  |  |  | 18 |  |  |  |  |  |  |  |  |  |  |  |
| 14 | 114 (17) |  |  |  |  |  |  |  |  |  | 17 |  |  |  |  |  |  |  |  |  |  |  |
| 18 | 48 (13) |  |  | 11 |  |  |  |  |  |  | 2 |  |  |  | 7 / 5 |  |  |  |  |  |  |  |
| 25 | 44 (3) |  |  |  |  |  |  |  |  |  | 3 |  |  |  |  |  |  |  |  |  |  |  |
| 26 | 53 (2) |  |  |  |  |  |  |  |  |  | 2 |  |  |  |  |  |  |  |  |  |  |  |
| 28 | 25 (3) |  |  |  |  |  |  |  |  |  | 3 |  |  |  |  |  |  |  |  |  |  |  |
| 31 | 88 (3) |  |  |  |  |  |  |  |  |  | 3 |  |  |  |  |  |  |  |  |  |  |  |
| 32 | 61 (2) |  |  |  |  |  |  |  |  |  | 2 |  |  |  |  |  |  |  |  |  |  |  |
| 34 | 155 (11) |  |  |  |  |  |  |  |  |  | 11 |  |  |  |  |  |  |  |  |  |  |  |
| 40 | 148 (33) |  |  |  |  |  |  |  |  |  | 33 |  |  |  |  |  |  |  |  |  |  |  |
| 46 | 93 (9) |  |  |  |  |  |  |  |  |  | 9 |  |  |  |  |  |  |  |  |  |  |  |
| 49 | 148 (22) |  |  |  |  |  |  |  |  |  | 22 |  |  |  |  |  |  |  |  |  |  |  |
| 55 | 160 (30) |  |  |  | 14 |  | 7 |  |  |  | 9 |  |  |  |  | 8 / 4 |  | 5 / 1 |  |  |  |  |
| 56 | 36 (9) |  |  |  |  |  |  | 8 |  |  | 1 |  |  |  |  |  |  |  | 4 / 2 |  |  |  |
| 59 | 93 (8) |  |  |  |  | 7 |  |  |  |  | 1 |  |  |  |  |  |  |  |  |  |  |  |
| 60 | 48 (6) |  |  | 5 |  |  |  |  |  |  | 1 |  |  |  |  |  |  |  |  |  |  |  |
| 61 | 52 (15) | 4 | 9 |  |  |  |  |  |  |  | 2 |  | 3 / 1 | 1 / 1 |  |  |  |  |  |  |  |  |
| 63 | 151 (23) |  |  |  |  |  | 8 |  | 6 |  | 9 |  |  |  |  |  |  | 8 / 7 |  | 4 / 2 |  |  |
| 64 | 118 (16) |  |  |  |  |  |  | 13 |  |  | 3 |  |  |  |  |  |  |  |  |  |  |  |
| 66 | 136 (6) |  |  |  |  |  |  |  | 5 |  | 1 |  |  |  |  |  |  |  |  |  |  |  |
| 71 | 143 (12) |  |  |  |  |  |  |  |  | 11 | 1 |  |  |  |  |  |  |  |  |  |  |  |
| 76 | 150 (5) |  |  |  |  |  |  |  | 4 |  | 1 |  |  |  |  |  |  |  |  |  |  |  |
| 78 | 25 (3) |  |  |  |  |  |  |  | 2 |  | 1 |  |  |  |  |  |  |  |  |  |  |  |
| 79 | 53 (14) |  |  |  |  |  |  |  | 6 | 5 | 3 |  |  |  |  |  |  |  |  | 5 / 1 |  |  |
| 88 | 16 (3) |  |  |  |  |  |  |  |  |  | 3 |  |  |  |  |  |  |  |  |  |  |  |
| 90 | 143 (16) |  |  |  |  |  |  |  |  |  | 16 |  |  |  |  |  |  |  |  |  |  |  |
| 91 | 156 (25) |  |  |  |  |  |  |  |  | 19 | 6 |  |  |  |  |  |  |  |  |  | 7 / 1 |  |
| 93 | 99 (9) |  |  |  |  |  |  |  |  |  | 9 |  |  |  |  |  |  |  |  |  |  |  |
| 96 | 15 (3) |  |  |  |  |  |  |  |  |  | 3 |  |  |  |  |  |  |  |  |  |  |  |
| 97 | 141 (19) |  |  |  |  |  |  |  | 8 |  | 11 |  |  |  |  |  |  |  |  |  |  |  |
| 98 | 152 (27) |  |  |  |  |  |  |  |  |  | 27 |  |  |  |  |  |  |  |  |  |  | 10 / 1 |
| 99 | 36 (10) |  |  |  |  |  |  |  |  | 7 | 3 |  |  |  |  |  |  |  |  |  | 7 / 5 |  |
| 101 | 157 (5) |  |  |  |  |  |  |  |  |  | 5 |  |  |  |  |  |  |  |  |  |  |  |
| 104 | 78 (23) |  |  |  |  |  |  |  |  |  | 23 |  |  |  |  |  |  |  |  |  |  | 6 / 1 |
| 111 | 160 (9) |  |  |  |  |  |  |  |  |  | 9 |  |  |  |  |  |  |  |  |  |  | 3 / 1 |
| 114 | 93 (7) |  |  |  |  |  |  |  |  |  | 7 |  |  |  |  |  |  |  |  |  |  | 5 / 1 |
| 115 | 47 (4) |  |  |  |  |  |  |  |  |  | 4 |  |  |  |  |  |  |  |  |  |  |  |
| 118 | 160 (9) |  |  |  |  |  |  |  |  |  | 9 |  |  |  |  |  |  |  |  |  |  | 2 / 1 |
| 119 | 34 (2) |  |  |  |  |  |  |  |  |  | 2 |  |  |  |  |  |  |  |  |  |  |  |
| 120 | 125 (2) |  |  |  |  |  |  |  |  |  | 2 |  |  |  |  |  |  |  |  |  |  |  |
| 123 | 129 (3) |  |  |  |  |  |  |  |  |  | 3 |  |  |  |  |  |  |  |  |  |  |  |
| 125 | 150 (9) |  |  |  |  |  |  |  |  |  | 9 |  |  |  |  |  |  |  |  |  |  |  |
| 126 | 36 (12) |  |  |  |  |  |  |  |  |  | 12 |  |  |  |  |  |  |  |  |  |  | 5 / 1 |
| 127 | 140 (18) |  |  |  |  |  |  |  |  |  | 18 |  |  |  |  |  |  |  |  |  |  |  |
| 128 | 50 (13) |  |  |  |  |  |  |  |  |  | 13 |  |  |  |  |  |  |  |  |  |  |  |
| 130 | 152 (11) |  |  |  |  |  |  |  |  |  | 11 |  |  |  |  |  |  |  |  |  |  |  |
| 135 | 18 (5) |  |  |  |  |  |  |  |  |  | 5 |  |  |  |  |  |  |  |  |  |  | 4 / 1 |
| 136 | 136 (34) |  |  |  |  |  |  |  |  |  | 34 |  |  |  |  |  |  |  |  |  |  | 10 / 4 |
| 139 | 141 (3) |  |  |  |  |  |  |  |  |  | 3 |  |  |  |  |  |  |  |  |  |  |  |
| 140 | 21 (5) |  |  |  |  |  |  |  |  |  | 5 |  |  |  |  |  |  |  |  |  |  |  |
| 141 | 109 (22) |  |  |  |  |  |  |  |  |  | 22 |  |  |  |  |  |  |  |  |  |  |  |
| 142 | 142 (6) |  |  |  |  |  |  |  |  |  | 6 |  |  |  |  |  |  |  |  |  |  |  |
| 145 | 88 (9) |  |  |  |  |  |  |  |  |  | 9 |  |  |  |  |  |  |  |  |  |  |  |
| 148 | 127 (31) |  |  |  |  |  |  |  |  |  | 31 |  |  |  |  |  |  |  |  |  |  | 21 / 3 |
| 152 | 120 (9) |  |  |  |  |  |  |  |  |  | 9 |  |  |  |  |  |  |  |  |  |  |  |
| 153 | 116 (10) |  |  |  |  |  |  |  |  |  | 10 |  |  |  |  |  |  |  |  |  |  |  |
| 163 | 22 (4) |  |  |  |  |  |  |  |  |  | 4 |  |  |  |  |  |  |  |  |  |  |  |
| 165 | 139 (13) |  |  |  |  |  |  |  |  |  | 13 |  |  |  |  |  |  |  |  |  |  |  |
| 169 | 161 (19) |  |  |  |  |  |  |  |  |  | 19 |  |  |  |  |  |  |  |  |  |  | 4 / 1 |
| 170 | 115 (36) |  |  |  |  |  |  |  |  |  | 36 |  |  |  |  |  |  |  |  |  |  | 19 / 3 |
| 171 | 36 (3) |  |  |  |  |  |  |  |  |  | 3 |  |  |  |  |  |  |  |  |  |  |  |
| 176 | 149 (26) | 7 | 11 | 3 |  |  |  |  |  |  | 5 |  | 6 / 5 | 2 / 1 |  |  |  |  |  |  |  |  |
| 178 | 36 (11) |  | 8 |  |  |  |  |  |  |  | 3 |  |  | 7 / 2 |  |  |  |  |  |  |  |  |
| 180 | 152 (32) |  |  | 7 | 9 |  | 7 |  |  |  | 9 |  |  |  | 7 / 5 | 5 / 3 |  |  |  |  |  |  |
| 182 | 60 (17) |  |  |  |  | 10 |  | 4 |  |  | 3 |  |  |  |  |  | 9 / 5 |  |  |  |  |  |
| 183 | 112 (17) |  |  |  |  |  |  | 8 | 4 |  | 5 |  |  |  |  |  |  |  | 2 / 1 | 3 / 1 |  |  |
| **Total** | | **11** | **28** | **26** | **23** | **17** | **22** | **33** | **35** | **42** | **674** |  | **9 / 6** | **10 / 4** | **14 / 10** | **13 / 7** | **9 / 5** | **13 / 8** | **6 / 3** | **12 / 4** | **14 / 6** | **93 / 19** |
| **All together** | | **911** (237 young; 674 adults) | | | | | | | | | | **193 / 72** | | | | | | | | | | |

***TABLE S6* The number of healthy ducks in interviewed households by age categories**

| **#** | **Total number of ducks** | **Healthy duck age category** (days) | | | | | | | | | |
| --- | --- | --- | --- | --- | --- | --- | --- | --- | --- | --- | --- |
|  |  | **7** | **14** | **21** | **30** | **45** | **60** | **90** | **120** | **150** | **≤180** |
| 5 | 152 (27) |  |  |  |  |  |  |  |  |  | 27 |
| 9 | 58 (14) |  |  |  |  |  |  |  |  |  | 14 |
| 10 | 142 (12) |  |  |  |  |  |  |  |  |  | 12 |
| 20 | 129 (16) |  | 12 |  |  |  |  |  |  |  | 4 |
| 30 | 134 (10) |  |  |  |  |  |  |  |  |  | 10 |
| 32 | 61 (7) | 6 |  |  |  |  |  |  |  |  | 1 |
| 34 | 155 (12) |  |  | 8 |  |  |  |  |  |  | 4 |
| 38 | 46 (11) |  |  |  |  |  |  |  |  |  | 11 |
| 47 | 65 (21) |  | 7 |  | 9 |  |  |  |  |  | 5 |
| 50 | 137 (13) |  |  |  |  | 11 |  |  |  |  | 2 |
| 51 | 69 (21) | 4 |  | 4 |  | 8 |  |  |  |  | 5 |
| 52 | 133 (3) |  |  |  |  |  |  |  |  |  | 3 |
| 55 | 160 (22) |  | 4 | 3 |  |  | 4 | 6 |  |  | 5 |
| 59 | 93 (11) |  |  |  |  | 2 |  |  | 7 |  | 2 |
| 68 | 160 (15) |  |  |  |  |  |  | 2 | 11 |  | 2 |
| 69 | 46 (15) |  |  |  | 4 |  |  |  | 9 |  | 2 |
| 70 | 24 (6) |  |  |  |  |  |  |  |  | 5 | 1 |
| 78 | 25 (5) |  |  |  |  |  |  |  | 4 |  | 1 |
| 81 | 156 (22) |  |  |  |  |  | 5 |  |  | 13 | 4 |
| 87 | 22 (8) |  |  |  |  |  |  |  |  | 6 | 2 |
| 91 | 156 (22) |  |  |  |  |  |  |  | **7** | 11 | 4 |
| 94 | 18 (4) |  |  |  |  |  |  |  |  |  | 4 |
| 97 | 141 (12) |  |  |  |  |  |  |  |  | 4 | 8 |
| 99 | 36 (8) |  |  |  |  |  |  |  |  | 5 | 3 |
| 100 | 133 (27) |  |  |  |  |  |  |  |  |  | 27 |
| 101 | 157 (5) |  |  |  |  |  |  |  |  |  | 5 |
| 102 | 52 (9) |  |  |  |  |  |  |  |  |  | 9 |
| 105 | 66 (4) |  |  |  |  |  |  |  |  |  | 4 |
| 109 | 138 (12) |  |  |  |  |  |  |  |  |  | 12 |
| 111 | 160 (7) |  |  |  |  |  |  |  |  |  | 7 |
| 118 | 160 (7) |  |  |  |  |  |  |  |  |  | 7 |
| 128 | 50 (11) |  |  |  |  |  |  |  |  |  | 11 |
| 134 | 79 (14) |  |  |  |  |  |  |  |  |  | 14 |
| 136 | 136 (31) |  |  |  |  |  |  |  |  |  | 31 |
| 137 | 22 (6) |  |  |  |  |  |  |  |  |  | 6 |
| 152 | 120 (4) |  |  |  |  |  |  |  |  |  | 4 |
| 153 | 116 (6) |  |  |  |  |  |  |  |  |  | 6 |
| 162 | 136 (112) |  |  |  |  |  |  |  |  |  | 112 |
| 167 | 141 (38) |  |  |  |  |  |  |  |  |  | 38 |
| 177 | 95 (37) |  |  | 16 |  | 12 |  |  |  |  | 9 |
| 181 | 132 (17) |  | 3 |  | 5 |  | 6 |  |  |  | 3 |
| 183 | 112 (11) |  |  |  |  |  | 7 |  |  |  | 4 |
| **Total** | | **10** | **26** | **31** | **18** | **33** | **22** | **8** | **38** | **44** | **445** |
| **All together** | | **675** (230 young; 445 adults) | | | | | | | | | |

FIGURE S1 Number of avian aspergillosis that started in each month during 2018-2019 (18 months) among interviewed households in the Almaty region, Republic of Kazakhstan

***TABLE S7* Household income loss due to reduced egg production**

| **Household’s code name** | **Chicken /**  **Daily egg yielding** | | **Daily egg loss**  (n) | **Duration of infection** (days) | **Egg price**  (₸ / $) | **Income loss due to reduced egg**  **production**  (₸ / $) | **Turkey /**  **Daily egg yielding** | | **Daily egg loss**  (n) | **Duration of infection** (days) | **Egg price**  (₸ / $) | **Income loss due to reduced egg**  **production**  (₸ / $) |
| --- | --- | --- | --- | --- | --- | --- | --- | --- | --- | --- | --- | --- |
|  | **Before infection** | **After infection** |  |  |  |  | **Before infection** | **After infection** |  |  |  |  |
| Ag_Oyt_017 | 82 | 55 | 27 | 11 | 60 / 0.12 | **17820**  **35.64** |  |  |  |  |  |  |
| Pa_Bas_022 | 73 | 52 | 21 | 12 | 60 / 0.12 | **15120**  **30.24** |  |  |  |  |  |  |
| Ra_Kai_025 | 39 | 24 | 15 | 8 | 60 / 0.12 | **7200**  **14.4** |  |  |  |  |  |  |
| Pa_Kon_031 | 81 | 61 | 20 | 8 | 60 / 0.12 | **9600**  **19.2** |  |  |  |  |  |  |
| Kn_Sat_038 | 33 | 18 | 15 | 5 | 60 / 0.12 | **4500**  **9** |  |  |  |  |  |  |
| En_Bay_045 | 36 | 15 | 21 | 12 | 60 / 0.12 | **15120**  **30.24** |  |  |  |  |  |  |
| Pa_Ulk _048 | 101 | 76 | 35 | 7 | 60 / 0.12 | **14700**  **29,4** |  |  |  |  |  |  |
| Ra_Bol_050 | 59 | 42 | 18 | 9 | 60 / 0.12 | **9720**  **19.44** |  |  |  |  |  |  |
| Kk_Kyz_052 | 62 | 46 | 16 | 11 | 60 / 0.12 | **10560**  **21.12** |  |  |  |  |  |  |
| Ag_Egi_059 | 44 | 25 | 19 | 7 | 60 / 0.12 | **7980**  **15.96** |  |  |  |  |  |  |
| Ba_Kuy_064 | 57 | 38 | 19 | 10 | 60 / 0.12 | **11400**  **22.8** |  |  |  |  |  |  |
| Ks_Sha_067 | 48 | 26 | 22 | 7 | 60 / 0.12 | **9240**  **18.48** |  |  |  |  |  |  |
| Kk_Sar_074 | 61 | 31 | 30 | 9 | 60 / 0.12 | **16200**  **32.4** |  |  |  |  |  |  |
| Ku_Kab_076 | 70 | 30 | 40 | 11 | 60 / 0.12 | **26400**  **52.8** |  |  |  |  |  |  |
| Ui_Kyr_077 | 85 | 45 | 40 | 10 | 60 / 0.12 | **24000**  **48** |  |  |  |  |  |  |
| Ba_Ber_081 | 92 | 52 | 40 | 7 | 60 / 0.12 | **16800**  **33.6** |  |  |  |  |  |  |
| En_Kar_085 | 75 | 44 | 31 | 9 | 60 / 0.12 | **16740**  **33.48** |  |  |  |  |  |  |
| Ba_Top_093 | 53 | 31 | 11 | 7 | 60 / 0.12 | **4620**  **9.24** |  |  |  |  |  |  |
| Ks_Sha_097 | 71 | 40 | 10 | 26 | 60 / 0.12 | **15600**  **31.2** |  |  |  |  |  |  |
| Ra_Sat_0101 | 122 | 76 | 46 | 11 | 60 / 0.12 | **30360**  **60.72** |  |  |  |  |  |  |
| Pa_Ulk_0106 | 114 | 77 | 37 | 26 | 60 / 0.12 | **57720**  **115.44** | 16 | 9 | 7 | 26 | 500 / 1 | **91000**  **182** |
| Ku_Ain_0109 | 101 | 44 | 57 | 26 | 60 / 0.12 | **88920**  **177.84** | 18 | 13 | 5 | 26 | 500 / 1 | **65000**  **130** |
| Ku_Zha_0112 | 127 | 102 | 25 | 9 | 60 / 0.12 | **13500**  **27** |  |  |  |  |  |  |
| Pa_Kon_0118 | 24 | 2 | 22 | 22 | 60 / 0.12 | **29040**  **58.08** | 106 | 100 | 6 | 22 | 500 / 1 | **66000**  **132** |
| Kk_Sha_0123 | 104 | 75 | 29 | 25 | 60 / 0.12 | **43500**  **87** | 15 | 7 | 8 | 25 | 500 / 1 | **100000**  **200** |
| Kk_Alt_0127 | 116 | 73 | 43 | 12 | 60 / 0.12 | **30960**  **61.92** |  |  |  |  |  |  |
| En_Koy_0132 | 77 | 41 | 36 | 24 | 60 / 0.12 | **51840**  **103.68** | 26 | 7 | 20 | 24 | 500 / 1 | **240000**  **480** |
| Ks_Irg_0134 | 62 | 28 | 34 | 9 | 60 / 0.12 | **18360**  **36.72** |  |  |  |  |  |  |
| Pa_Bir_0138 | 59 | 29 | 30 | 11 | 60 / 0.12 | **19800**  **39.6** |  |  |  |  |  |  |
| Pa_Bas_0139 | 117 | 71 | 46 | 22 | 60 / 0.12 | **60720**  **121.44** | 12 | 8 | 6 | 22 | 500 / 1 | **66000**  **132** |
| Kt_Tas_0142 | 108 | 60 | 48 | 9 | 60 / 0.12 | **25920**  **51.84** |  |  |  |  |  |  |
| Kt_Esk_0145 | 72 | 24 | 48 | 12 | 60 / 0.12 | **34560**  **69.12** |  |  |  |  |  |  |
| Kn_Bol_0146 | 74 | 40 | 34 | 26 | 60 / 0.12 | **53040**  **106.08** | 21 | 9 | 12 | 26 | 500 / 1 | **156000**  **312** |
| Ks_Ber_0154 | 79 | 54 | 25 | 11 | 60 / 0.12 | **16500**  **33** |  |  |  |  |  |  |
| Ag_Egi_0155 | 83 | 47 | 36 | 7 | 60 / 0.12 | **15120**  **30.24** |  |  |  |  |  |  |
| Tr_Dos_0158 | 92 | 66 | 26 | 9 | 60 / 0.12 | **14040**  **28.08** |  |  |  |  |  |  |
| Ra_Kai_0160 | 55 | 22 | 33 | 24 | 60 / 0.12 | **47520**  **95.04** | 58 | 35 | 23 | 24 | 500 / 1 | **276000**  **552** |
| Ui_Cha_0164 | 80 | 51 | 29 | 12 | 60 / 0.12 | **20880**  **41.76** |  |  |  |  |  |  |
| Ra_Aul_0166 |  |  |  |  |  |  | 89 | 50 | 39 | 12 | 500 / 1 | **234000**  **468** |
| Tr_Bes_0167 | 53 | 18 | 35 | 20 | 60 / 0.12 | **42000**  **84** | 37 | 15 | 22 | 20 | 500 / 1 | **220000**  **440** |
| En_Bay_0170 | 74 | 34 | 40 | 22 | 60 / 0.12 | **52800**  **105.6** |  |  |  |  |  |  |
| Ks_Ayt_0177 | 11 | 5 | 6 | 10 | 60 / 0.12 | **3600**  **7.2** |  |  |  |  |  |  |
| **Total** | **3026** | **1790** | **1215** | **545** | **60 / 0.12** | **39730500**  **79461** | **398** | **253** | **148** | **227** | **500 / 1** | **16798000**  **33596** |

***TABLE S8* Financial losses due to avian aspergillosis in households in the Almaty region, Republic of Kazakhstan**

| **#** | **Value of the poultry before the outbreak**  **(₸ / $)** | | **Value of the poultry after the outbreak**  **(₸ / $)** | | **Loss due to mortality**  **(₸ / $)** | | **Value of the affected poultry kept**  **(₸ / $)** | | **Value of the unaffected poultry**  **(₸ / $)** | | **Treatment cost**  **(₸ / $)** | | **Financial losses due to reduced egg production (₸ / $)** | | **Total loss**  **(₸ / $)** | | **% of the value of the poultry loss** | **Affected poultry species** |
| --- | --- | --- | --- | --- | --- | --- | --- | --- | --- | --- | --- | --- | --- | --- | --- | --- | --- | --- |
| 1 | 48500 | 97 | 40500 | 81 | 3500 | 7 | 4500 | 9 | 36000 | 72 | 3100 | 6,2 | - | - | 11100 | 22,2 | 16,5 | Chicken |
| 2 | 51000 | 102 | 41250 | 82,5 | 6000 | 12 | 3750 | 7,5 | 37500 | 75 | 256 | 0,5 | - | - | 10006 | 20 | 19,1 |  |
| 3 | 43000 | 86 | 32750 | 65,5 | 4000 | 8 | 6250 | 12,5 | 26500 | 53 | 3100 | 6,2 | - | - | 13350 | 26,7 | 23,8 |  |
| 4 | 442500 | 885 | 382000 | 764 | 23500 | 47 | 37000 | 74 | 345000 | 690 | 3718 | 7,4 | - | - | 64218 | 128,4 | 13,7 | Chicken and turkey |
| 5 | 869000 | 1738 | 741000 | 1482 | 14000 | 28 | 53000 | 106 | 688000 | 1376 | 30400 | 60,8 | - | - | 158400 | 316,8 | 14,7 |  |
| 6 | 262000 | 524 | 246750 | 493,5 | 3000 | 6 | 15250 | 30,5 | 231500 | 463 | 69600 | 139,2 | - | - | 84850 | 169,7 | 5,8 | Chicken |
| 7 | 403000 | 806 | 356000 | 712 | 15000 | 30 | 47000 | 94 | 309000 | 618 | 7680 | 15,4 | - | - | 54680 | 109,4 | 11,7 | Chicken and turkey |
| 8 | 639500 | 1279 | 439000 | 878 | 39000 | 78 | 48750 | 97,5 | 390250 | 780,5 | 38400 | 76,8 | - | - | 238900 | 477,8 | 31,4 | Chicken, turkey and goose |
| 9 | 437000 | 874 | 373500 | 747 | 13500 | 27 | 63500 | 127 | 310000 | 620 | 77280 | 154,6 | - | - | 140780 | 281,6 | 14,5 | Chicken and turkey |
| 10 | 885000 | 1770 | 850250 | 1700,5 | 10500 | 21 | 24250 | 48,5 | 826000 | 1652 | 25600 | 51,2 | - | - | 60350 | 120,7 | 3,9 | Chicken |
| 11 | 185500 | 371 | 173000 | 346 | 3500 | 7 | 10750 | 21,5 | 162250 | 324,5 | 30400 | 60,8 | - | - | 42900 | 85,8 | 6,7 |  |
| 12 | 1317500 | 2635 | 1173500 | 2347 | 37000 | 74 | 107000 | 214 | 1066500 | 2133 | 32940 | 65,9 | - | - | 176940 | 353,9 | 10,9 | Chicken and turkey |
| 13 | 552000 | 1104 | 483000 | 966 | 22500 | 45 | 46500 | 93 | 436500 | 873 | 38400 | 76,8 | - | - | 107400 | 214,8 | 12,5 |  |
| 14 | 744500 | 1509 | 707500 | 1415 | 37000 | 74 | 126250 | 252,5 | 581250 | 1162,2 | 92000 | 184 | - | - | 129000 | 258 | 5 |  |
| 15 | 170000 | 340 | 159500 | 319 | 3500 | 7 | 7000 | 14 | 152500 | 305 | 1352 | 2,7 | - | - | 11852 | 23,7 | 6,2 | Chicken |
| 16 | 884000 | 1768 | 725000 | 1450 | 70000 | 140 | 89000 | 178 | 636000 | 1272 | 21960 | 43,9 | - | - | 180960 | 361,9 | 18 | Turkey |
| 17 | 538920 | 1077,8 | 473620 | 947,2 | 16000 | 32 | 46000 | 92 | 427620 | 855,2 | 0 | 0 | 17820 | 35,6 | 83120 | 166,2 | 12,1 | Chicken |
| 18 | 88400 | 176,8 | 65200 | 130,4 | 17400 | 34,8 | 5900 | 11,8 | 59300 | 118,6 | 60800 | 121,6 | - | - | 84000 | 168 | 26,3 | Chicken and goose |
| 19 | 366600 | 733,2 | 334200 | 668,4 | 18800 | 37,6 | 13600 | 27,2 | 320600 | 641,2 | 10560 | 21,1 | - | - | 42960 | 85,9 | 8,8 | Chicken |
| 20 | 104900 | 209,8 | 82950 | 165,5 | 18900 | 37,8 | 3050 | 6,1 | 79900 | 159,8 | 58080 | 116,2 | - | - | 80030 | 160 | 20,1 |  |
| 21 | 996500 | 1993 | 921500 | 1843 | 10000 | 20 | 65000 | 130 | 856500 | 1713 | 17600 | 35,2 | - | - | 92600 | 185,2 | 7,5 | Turkey |
| 22 | 338000 | 676 | 300500 | 601 | 12000 | 24 | 25500 | 51 | 275000 | 550 | 2486 | 5 | 15120 | 30,2 | 55106 | 110,2 | 11,1 | Chicken |
| 23 | 87400 | 174,8 | 74500 | 149 | 8600 | 17,2 | 3500 | 7 | 71000 | 142 | 10560 | 21,1 | - | - | 23460 | 46,9 | 14,8 | Chicken and turkey |
| 24 | 1077900 | 2155,8 | 1059050 | 2118,1 | 8300 | 16,6 | 10550 | 21,1 | 1048500 | 2097 | 6400 | 12,8 | - | - | 25250 | 50,5 | 1,8 | Chicken |
| 25 | 194000 | 388 | 158000 | 316 | 12000 | 24 | 24000 | 48 | 134000 | 268 | 21630 | 43,3 | 7200 | 14,4 | 64830 | 129,7 | 18,6 |  |
| 26 | 111700 | 223,4 | 106200 | 212,4 | 5500 | 11 | 0 | 0 | 106200 | 212,4 | 0 | 0 | - | - | 5500 | 11 | 4,9 |  |
| 27 | 167200 | 334,4 | 113350 | 226,7 | 23300 | 46,6 | 2650 | 5,3 | 110700 | 221,4 | 17600 | 35,2 | - | - | 71450 | 142,9 | 32,2 |  |
| 28 | 118000 | 236 | 110000 | 220 | 12000 | 24 | 16000 | 32 | 94000 | 188 | 5940 | 11,9 | - | - | 13940 | 27,9 | 6,8 |  |
| 29 | 252000 | 504 | 198000 | 396 | 12000 | 24 | 42000 | 84 | 156000 | 312 | 0 | 0 | - | - | 54000 | 108 | 21,4 | Turkey |
| 30 | 990000 | 1980 | 830000 | 1660 | 36000 | 72 | 124000 | 248 | 706000 | 1412 | 121440 | 242,9 | - | - | 281440 | 562,9 | 16,2 | Chicken and turkey |
| 31 | 370000 | 740 | 328000 | 656 | 4000 | 8 | 38000 | 76 | 290000 | 580 | 13440 | 26,9 | 9600 | 19,2 | 65040 | 130,1 | 11,4 | Chicken |
| 32 | 187700 | 375,4 | 179400 | 358,8 | 7600 | 15,2 | 1400 | 2,8 | 178000 | 356 | 7680 | 15,4 | - | - | 15980 | 32 | 4,4 |  |
| 33 | 664000 | 1328 | 514000 | 1028 | 132000 | 264 | 18000 | 36 | 496000 | 992 | 5580 | 11,2 | - | - | 155580 | 311,2 | 22,6 | Turkey |
| 34 | 704800 | 1409,6 | 693600 | 1387,2 | 9400 | 18,8 | 1800 | 3,6 | 691800 | 1383,6 | 7680 | 15,4 | - | - | 18880 | 37,8 | 1,6 | Chicken |
| 35 | 940000 | 1880 | 826000 | 1652 | 40000 | 80 | 74000 | 148 | 752000 | 1504 | 11520 | 23 | - | - | 125520 | 251 | 12,1 | Chicken and turkey |
| 36 | 332000 | 664 | 276000 | 552 | 20000 | 40 | 48000 | 96 | 228000 | 456 | 2800 | 5,6 | - | - | 58800 | 117,6 | 16,9 | Chicken |
| 37 | 48000 | 96 | 38000 | 76 | 8000 | 16 | 2000 | 4 | 36000 | 72 | 3520 | 7 | - | - | 13520 | 27 | 20,8 |  |
| 38 | 173000 | 346 | 141000 | 282 | 4000 | 8 | 28000 | 56 | 113000 | 226 | 3100 | 6,2 | 4500 | 9 | 39600 | 79,2 | 18,5 |  |
| 39 | 1380000 | 2760 | 1170000 | 2340 | 60000 | 120 | 150000 | 300 | 1020000 | 2040 | 21630 | 43,3 | - | - | 231630 | 463,3 | 15,2 | Turkey |
| 40 | 1294000 | 2588 | 1142000 | 2284 | 52000 | 104 | 100000 | 200 | 1042000 | 2084 | 100800 | 201,6 | - | - | 252800 | 505,6 | 11,8 | Chicken and turkey |
| 41 | 164000 | 328 | 136000 | 272 | 8000 | 16 | 20000 | 40 | 116000 | 232 | 8800 | 17,6 | - | - | 36800 | 73,6 | 17,1 | Chicken |
| 42 | 1360000 | 2720 | 1240000 | 2480 | 24000 | 48 | 96000 | 192 | 1144000 | 2288 | 0 | 0 | - | - | 120000 | 240 | 8,8 | Turkey |
| 43 | 471000 | 942 | 400050 | 800,1 | 25600 | 51,2 | 45350 | 90,7 | 354700 | 709,4 | 13440 | 26,9 | - | - | 84390 | 168,8 | 15,1 | Chicken |
| 44 | 28000 | 56 | 18000 | 36 | 4000 | 8 | 6000 | 12 | 12000 | 24 | 3168 | 6,3 | - | - | 13168 | 26,3 | 35,7 |  |
| 45 | 228000 | 456 | 182000 | 364 | 8000 | 16 | 38000 | 76 | 144000 | 288 | 0 | 0 | 15120 | 30,2 | 61120 | 122,2 | 20,2 |  |
| 46 | 954000 | 1908 | 772000 | 1544 | 40000 | 80 | 142000 | 284 | 630000 | 1260 | 95040 | 190,1 | - | - | 277040 | 554,1 | 19,1 | Chicken and turkey |
| 47 | 85600 | 171,2 | 59400 | 118,8 | 15000 | 30 | 11200 | 22,4 | 48200 | 96,4 | 8800 | 17,6 | - | - | 35000 | 70 | 30,6 | Chicken |
| 48 | 548000 | 1096 | 462000 | 924 | 32000 | 64 | 54000 | 108 | 408000 | 816 | 31680 | 63,4 | 14700 | 29,4 | 132380 | 264,8 | 15,7 |  |
| 49 | 1388000 | 2776 | 1202000 | 2404 | 84000 | 168 | 102000 | 204 | 1100000 | 2200 | 11520 | 23 | - | - | 197520 | 395 | 13,4 | Chicken and turkey |
| 50 | 311500 | 623 | 298850 | 597,7 | 12800 | 25,6 | 1550 | 3,1 | 297300 | 594,6 | 15360 | 30,7 | 9720 | 19,4 | 37730 | 75,5 | 4,1 | Chicken |
| 51 | 146500 | 293 | 135150 | 270,3 | 4900 | 9,8 | 5450 | 10,9 | 129700 | 259,4 | 4400 | 8,8 | - | - | 15750 | 31,5 | 7,8 |  |
| 52 | 391400 | 782,8 | 343000 | 686 | 16800 | 33,6 | 31600 | 63,2 | 311400 | 622,8 | 17600 | 35,2 | 10560 | 21,1 | 76560 | 153,12 | 12,4 |  |
| 53 | 187000 | 374 | 146500 | 293 | 23900 | 47,8 | 4100 | 8,2 | 142400 | 284,8 | 18400 | 36,8 | - | - | 58900 | 117,8 | 21,7 | Chicken and turkey |
| 54 | 790400 | 1508,8 | 674400 | 1348,8 | 34800 | 69,6 | 81200 | 162,4 | 593200 | 1186,4 | 42240 | 84,5 | - | - | 158240 | 316,5 | 14,7 |  |
| 55 | 617700 | 1235,4 | 552150 | 1104,3 | 43400 | 86,8 | 22150 | 44,3 | 530000 | 1060 | 33270 | 66,5 | - | - | 98820 | 197,6 | 10,6 | Chicken, turkey and goose |
| 56 | 105000 | 210 | 74250 | 148,5 | 18500 | 37 | 12250 | 24,5 | 62000 | 124 | 6336 | 12,7 | - | - | 37086 | 74,2 | 29,3 | Chicken and goose |
| 57 | 524817 | 1049,6 | 420367 | 840,7 | 45100 | 90,2 | 55850 | 111,7 | 364517 | 729 | 33270 | 66,5 | - | - | 137720 | 275,4 | 19,9 | Chicken and turkey |
| 58 | 585700 | 1171,4 | 542800 | 1085,6 | 34100 | 68,2 | 8800 | 17,6 | 534000 | 1068 | 29920 | 59,8 | - | - | 72820 | 145,6 | 7,3 |  |
| 59 | 244800 | 489,6 | 202250 | 404,5 | 7500 | 15 | 35050 | 70,1 | 167200 | 334,4 | 10560 | 21.1 | 7980 | 16 | 61090 | 122,2 | 17,4 | Chicken |
| 60 | 112000 | 224 | 101000 | 202 | 8000 | 16 | 3000 | 6 | 98000 | 196 | 1584 | 3,2 | - | - | 12584 | 25,2 | 9,8 |  |
| 61 | 205300 | 410,6 | 191950 | 383,9 | 11700 | 23,4 | 3850 | 7,7 | 188100 | 376,2 | 3549 | 7,1 | - | - | 16899 | 33,8 | 6,5 | Chicken and goose |
| 62 | 448700 | 897,4 | 335200 | 670,4 | 66800 | 133,6 | 46700 | 93,4 | 288500 | 577 | 42240 | 84,5 | - | - | 155740 | 311,5 | 25,3 | Chicken and turkey |
| 63 | 770500 | 1541 | 625000 | 1250 | 121000 | 242 | 24000 | 48 | 601000 | 1202 | 48000 | 96 | - | - | 193500 | 387 | 18,9 | Chicken, turkey and goose |
| 64 | 385200 | 770,4 | 322000 | 644 | 30800 | 61,6 | 32400 | 64,8 | 289600 | 579,2 | 0 | 0 | 11400 | 22,8 | 74600 | 149,2 | 16,4 | Chicken |
| 65 | 27000 | 54 | 18750 | 37,5 | 6000 | 12 | 2250 | 4,5 | 16500 | 33 | 1408 | 2,8 | - | - | 9658 | 19,3 | 30,6 |  |
| 66 | 600500 | 1201 | 525250 | 1050,5 | 52500 | 105 | 22750 | 45,5 | 502500 | 1005 | 3200 | 6,4 | - | - | 78450 | 156,9 | 12,5 | Turkey |
| 67 | 496500 | 993 | 416250 | 832,5 | 28500 | 57 | 51750 | 103,5 | 364500 | 729 | 11520 | 23 | 9240 | 18,5 | 101010 | 202 |  | Chicken |
| 68 | 384200 | 768,4 | 307700 | 615,4 | 69000 | 138 | 7500 | 15 | 300200 | 600,4 | 31680 | 63,4 | - | - | 108180 | 216,4 | 19,9 | Chicken and turkey |
| 69 | 91300 | 182,6 | 65300 | 130,6 | 20000 | 40 | 6000 | 12 | 59300 | 118,6 | 2464 | 4,9 | - | - | 28464 | 56,9 | 28,5 | Chicken |
| 70 | 37000 | 74 | 28000 | 56 | 8000 | 16 | 1000 | 2 | 27000 | 54 | 1584 | 3,2 | - | - | 10584 | 21,2 | 24,3 |  |
| 71 | 438500 | 877 | 411650 | 823,3 | 51000 | 102 | 8250 | 16,5 | 403400 | 806,8 | 0 | 0 | - | - | 26850 | 53,7 | 6,1 | Turkey |
| 72 | 303500 | 607 | 211000 | 422 | 55000 | 110 | 37500 | 75 | 173500 | 347 | 42240 | 84,5 | - | - | 134740 | 269,5 | 30,5 | Chicken and turkey |
| 73 | 776000 | 1552 | 535000 | 1070 | 97500 | 195 | 143500 | 287 | 391500 | 783 | 7680 | 15,4 | - | - | 248680 | 497,4 | 31,1 | Turkey |
| 74 | 434500 | 869 | 353500 | 707 | 27000 | 54 | 57000 | 114 | 296500 | 593 | 28160 | 56,3 | 16200 | 32,4 | 125360 | 250,7 | 18,7 | Chicken |
| 75 | 36000 | 72 | 28000 | 56 | 4000 | 8 | 4000 | 8 | 24000 | 48 | 1408 | 2,8 | - | - | 9408 | 18,8 | 22,2 |  |
| 76 | 468500 | 937 | 355500 | 711 | 45000 | 90 | 68000 | 136 | 287500 | 575 | 35200 | 70,4 | 26400 | 52,8 | 174600 | 349,2 | 24,1 |  |
| 77 | 475000 | 950 | 387000 | 774 | 16000 | 32 | 72000 | 144 | 315000 | 630 | 43200 | 86,4 | 24000 | 48 | 155200 | 310,4 | 18,5 |  |
| 78 | 80200 | 160,4 | 67700 | 135,4 | 7500 | 15 | 5000 | 10 | 62700 | 125,4 | 1408 | 2,8 | - | - | 13908 | 27,8 | 15,6 |  |
| 79 | 177000 | 354 | 135000 | 270 | 19000 | 38 | 23000 | 46 | 112000 | 224 | 5760 | 11,5 | - | - | 47760 | 95,5 | 23,7 | Chicken and goose |
| 80 | 1058500 | 2117 | 862500 | 1725 | 36000 | 72 | 164000 | 328 | 698500 | 1397 | 5600 | 11,2 | - | - | 201600 | 403,2 | 18,5 | Chicken and turkey |
| 81 | 593200 | 1186,4 | 499200 | 998,4 | 28000 | 56 | 66000 | 132 | 433200 | 866,4 | 21120 | 42,2 | 16800 | 33,6 | 311920 | 263,8 | 15,9 | Chicken |
| 82 | 48000 | 96 | 28000 | 56 | 8000 | 16 | 12000 | 24 | 16000 | 32 | 3168 | 6,3 | - | - | 23168 | 46,3 | 41,7 |  |
| 83 | 636500 | 1273 | 453000 | 906 | 103000 | 206 | 75500 | 151 | 377500 | 755 | 33270 | 66,5 | - | - | 216770 | 433,5 | 28,8 | Chicken and turkey |
| 84 | 30000 | 60 | 15000 | 30 | 12000 | 24 | 3000 | 6 | 12000 | 24 | 1936 | 3,9 | - | - | 16936 | 33,9 | 50 | Chicken |
| 85 | 628000 | 1256 | 562000 | 1124 | 8000 | 16 | 58000 | 116 | 504000 | 1008 | 15360 | 30,7 | 16740 | 33,5 | 98100 | 196,2 | 10,5 |  |
| 86 | 200000 | 400 | 152000 | 304 | 12000 | 24 | 36000 | 72 | 116000 | 232 | 0 | 0 | - | - | 48000 | 96 | 24 | Turkey |
| 87 | 70400 | 140,8 | 48400 | 96,8 | 4000 | 8 | 18000 | 36 | 30400 | 60,8 | 1760 | 3,5 | - | - | 23760 | 47,5 | 31,2 | Chicken |
| 88 | 62000 | 124 | 46000 | 92 | 12000 | 24 | 4000 | 8 | 42000 | 84 | 35200 | 70,4 | - | - | 51200 | 102,4 | 25,8 |  |
| 89 | 1668000 | 3336 | 1458000 | 2916 | 24000 | 48 | 186000 | 372 | 1272000 | 2544 | 28160 | 56,3 | - | - | 238160 | 476,3 | 12,6 | Turkey |
| 90 | 668000 | 1336 | 622000 | 1244 | 20000 | 40 | 26000 | 52 | 596000 | 1192 | 15840 | 31,7 | - | - | 61840 | 123,7 | 6,9 | Chicken |
| 91 | 619500 | 1239 | 418500 | 837 | 32500 | 65 | 20000 | 40 | 398500 | 797 | 32940 | 65,9 | - | - | 233940 | 467,9 | 32,5 | Chicken, turkey and goose |
| 92 | 172000 | 344 | 148000 | 296 | 8000 | 16 | 16000 | 32 | 132000 | 264 | 1130 | 2,3 | - | - | 25130 | 50б3 | 14 | Chicken |
| 93 | 402000 | 804 | 353000 | 706 | 19000 | 38 | 30000 | 60 | 323000 | 646 | 10560 | 21,1 | 4620 | 8,6 | 64180 | 128,4 | 12,2 |  |
| 94 | 68000 | 136 | 48000 | 96 | 8000 | 16 | 12000 | 24 | 36000 | 72 | 0 | 0 | - | - | 20000 | 40 | 29,4 |  |
| 95 | 946500 | 1893 | 752250 | 1504,5 | 40500 | 81 | 129750 | 259,5 | 622500 | 1245 | 11520 | 23 | - | - | 205770 | 411,5 | 20,5 | Chicken and turkey |
| 96 | 78000 | 156 | 64000 | 128 | 4000 | 8 | 10000 | 20 | 54000 | 108 | 2816 | 5,6 | - | - | 16816 | 33,6 | 18 | Chicken |
| 97 | 675100 | 1350 | 545600 | 1091 | 107000 | 214 | 22500 | 45 | 523100 | 1046 | 42240 | 84,5 | 15600 | 31,2 | 187340 | 374,7 | 19,2 | Chicken and turkey |
| 98 | 1202000 | 2404 | 973000 | 1946 | 58000 | 116 | 171000 | 342 | 802000 | 1604 | 0 | 0 | - | - | 229000 | 458 | 19,1 | Chicken, turkey and goose |
| 99 | 153000 | 306 | 103000 | 206 | 29000 | 58 | 21000 | 42 | 82000 | 164 | 20240 | 40,5 | - | - | 70240 | 140,5 | 32,7 | Chicken and goose |
| 100 | 505000 | 1010 | 445000 | 890 | 20000 | 40 | 40000 | 80 | 405000 | 810 | 25600 | 51,2 | - | - | 85600 | 171,2 | 11,9 | Chicken |
| 101 | 821000 | 1642 | 707000 | 1414 | 44000 | 88 | 70000 | 140 | 637000 | 1274 | 2800 | 5,6 | 30360 | 60,7 | 147160 | 294,3 | 13,9 |  |
| 102 | 295000 | 590 | 207000 | 414 | 20000 | 40 | 68000 | 136 | 139000 | 278 | 44160 | 88,3 | - | - | 132160 | 264,3 | 29,8 | Chicken and turkey |
| 103 | 1292000 | 2584 | 1026000 | 2052 | 96000 | 192 | 170000 | 340 | 856000 | 1712 | 18400 | 36,8 | - | - | 284400 | 568,8 | 20,6 |  |
| 104 | 436500 | 873 | 367500 | 735 | 14000 | 28 | 51000 | 102 | 316500 | 633 | 57600 | 115,2 | - | - | 126600 | 253,2 | 15,8 | Chicken and goose |
| 105 | 412000 | 824 | 370000 | 740 | 20000 | 40 | 22000 | 44 | 348000 | 696 | 12320 | 24,6 | - | - | 54320 | 108,6 | 10,2 | Chicken |
| 106 | 704000 | 1408 | 564000 | 1128 | 48000 | 96 | 92000 | 184 | 472000 | 944 | 0 | 0 | 148720 | 297,4 | 288720 | 577,4 | 19,9 | Chicken and turkey |
| 107 | 60000 | 120 | 36000 | 72 | 4000 | 8 | 20000 | 40 | 16000 | 32 | 7040 | 14,1 | - | - | 31040 | 62,1 | 40 | Chicken |
| 108 | 704000 | 1408 | 574000 | 1148 | 36000 | 72 | 94000 | 188 | 480000 | 960 | 21960 | 43,9 | - | - | 151960 | 303,9 | 18,5 |  |
| 109 | 700000 | 1400 | 522000 | 1044 | 68000 | 136 | 110000 | 220 | 412000 | 824 | 132000 | 264 | 153920 | 307,8 | 463920 | 927,8 | 25,4 | Chicken and turkey |
| 110 | 52000 | 104 | 38000 | 76 | 4000 | 8 | 10000 | 20 | 28000 | 56 | 1183 | 2,4 | - | - | 15183 | 30,4 | 26,9 | Chicken |
| 111 | 1623000 | 3246 | 1191000 | 2382 | 195000 | 390 | 237000 | 474 | 954000 | 1908 | 130560 | 261,1 | - | - | 562560 | 1125,1 | 26,6 | Chicken, turkey and goose |
| 112 | 668000 | 1336 | 590000 | 1180 | 56000 | 112 | 22000 | 44 | 568000 | 1136 | 25600 | 51,2 | 13500 | 27 | 117100 | 234,2 | 11,7 | Chicken |
| 113 | 308000 | 616 | 258000 | 516 | 20000 | 40 | 30000 | 60 | 228000 | 456 | 17600 | 35,2 | - | - | 67600 | 135,2 | 16,2 |  |
| 114 | 606000 | 1212 | 490000 | 980 | 46000 | 92 | 70000 | 140 | 420000 | 840 | 0 | 0 | - | - | 116000 | 232 | 19,2 | Chicken and goose |
| 115 | 308000 | 616 | 228000 | 456 | 36000 | 72 | 64000 | 128 | 164000 | 328 | 35200 | 70,4 | - | - | 115200 | 230,4 | 26 | Chicken and turkey |
| 116 | 504000 | 1008 | 308000 | 616 | 60000 | 120 | 136000 | 272 | 172000 | 344 | 10240 | 20,5 | - | - | 206240 | 412,5 | 38,9 |  |
| 117 | 308000 | 616 | 280000 | 560 | 4000 | 8 | 24000 | 48 | 256000 | 512 | 2560 | 5,1 | - | - | 30560 | 61,1 | 9,1 | Chicken |
| 118 | 623000 | 1246 | 514000 | 1028 | 38000 | 76 | 71000 | 142 | 443000 | 886 | 36960 | 73,9 | 95040 | 190,1 | 241000 | 482 | 17,5 | Chicken, turkey and goose |
| 119 | 236000 | 472 | 214000 | 428 | 8000 | 16 | 14000 | 28 | 200000 | 400 | 6400 | 12,8 | - | - | 28400 | 56,8 | 9,3 | Chicken |
| 120 | 600000 | 1200 | 482000 | 964 | 44000 | 88 | 74000 | 148 | 408000 | 816 | 46080 | 92,2 | - | - | 164080 | 328,2 | 19,7 | Chicken and turkey |
| 121 | 768000 | 1538 | 660000 | 1320 | 44000 | 88 | 64000 | 128 | 596000 | 1192 | 21960 | 43,9 | - | - | 129960 | 259,9 | 14,1 | Chicken |
| 122 | 56000 | 112 | 40000 | 80 | 4000 | 8 | 12000 | 24 | 28000 | 56 | 1280 | 2,6 | - | - | 17280 | 345,6 | 28,6 |  |
| 123 | 670000 | 1340 | 542000 | 1084 | 44000 | 88 | 84000 | 168 | 458000 | 916 | 46080 | 92,2 | 143500 | 287 | 317580 | 635,2 | 19,1 | Chicken and turkey |
| 124 | 68000 | 136 | 36000 | 72 | 12000 | 24 | 20000 | 40 | 16000 | 32 | 1352 | 2,7 | - | - | 33352 | 66,7 | 47,1 | Chicken |
| 125 | 1438000 | 2876 | 1144000 | 2288 | 108000 | 216 | 186000 | 372 | 958000 | 1916 | 0 | 0 | - | - | 294000 | 588 | 20,5 | Turkey |
| 126 | 216000 | 432 | 142000 | 284 | 22000 | 44 | 40000 | 80 | 102000 | 204 | 40480 | 81 | - | - | 114480 | 229 | 34,3 | Chicken and goose |
| 127 | 506000 | 1012 | 392000 | 784 | 56000 | 112 | 58000 | 116 | 334000 | 668 | 38720 | 77,4 | 30960 | 61,9 | 183680 | 367,4 | 22,5 | Chicken |
| 128 | 150000 | 300 | 122000 | 244 | 8000 | 16 | 20000 | 40 | 102000 | 204 | 8800 | 17,6 | - | - | 36800 | 73,6 | 18,7 |  |
| 129 | 820000 | 1640 | 614000 | 1228 | 92000 | 184 | 114000 | 228 | 500000 | 1000 | 200000 | 400 | - | - | 406000 | 812 | 25,1 | Chicken and turkey |
| 130 | 959000 | 1918 | 665000 | 1330 | 60000 | 120 | 50000 | 100 | 615000 | 1230 | 32940 | 65,9 | - | - | 326940 | 653,9 | 30,7 |  |
| 131 | 64000 | 128 | 48000 | 96 | 8000 | 16 | 8000 | 16 | 40000 | 80 | 640 | 1,3 | - | - | 16640 | 33,3 | 25 | Chicken |
| 132 | 684000 | 1368 | 460000 | 920 | 64000 | 128 | 160000 | 320 | 300000 | 600 | 80960 | 161,9 | 291840 | 583,7 | 596800 | 1193,6 | 32,8 | Chicken and turkey |
| 133 | 1432000 | 2864 | 982000 | 1964 | 276000 | 552 | 174000 | 348 | 808000 | 1616 | 84640 | 169,3 | - | - | 534640 | 1069,3 | 31,4 | Turkey |
| 134 | 302000 | 604 | 228000 | 456 | 12000 | 24 | 62000 | 124 | 166000 | 332 | 28160 | 56,3 | 18360 | 36,7 | 120520 | 241 | 24,5 | Chicken |
| 135 | 102000 | 204 | 51000 | 102 | 14000 | 28 | 37000 | 74 | 14000 | 28 | 0 | 0 | - | - | 51000 | 102 | 50 | Chicken and goose |
| 136 | 717000 | 1434 | 514000 | 1028 | 108000 | 216 | 85000 | 170 | 429000 | 858 | 77280 | 154,6 | - | - | 280280 | 560,6 | 28,3 |  |
| 137 | 82000 | 164 | 68000 | 136 | 4000 | 8 | 10000 | 20 | 58000 | 116 | 7040 | 14,1 | - | - | 21040 | 42,1 | 17,1 | Chicken |
| 138 | 488000 | 976 | 412000 | 824 | 32000 | 64 | 44000 | 88 | 368000 | 736 | 4800 | 9,6 | 19800 | 39,6 | 100600 | 201,2 | 15,6 |  |
| 139 | 694000 | 1388 | 534000 | 1068 | 64000 | 128 | 96000 | 192 | 438000 | 876 | 77280 | 154,6 | 126720 | 253,4 | 364000 | 728 | 23,1 | Chicken and turkey |
| 140 | 84000 | 168 | 54000 | 108 | 8000 | 16 | 22000 | 44 | 32000 | 64 | 7920 | 15,8 | - | - | 37920 | 75,8 | 35,7 | Chicken |
| 141 | 832000 | 1664 | 624000 | 1248 | 72000 | 144 | 136000 | 272 | 488000 | 976 | 115200 | 230,4 | - | - | 323200 | 646,4 | 25 | Chicken and turkey |
| 142 | 812000 | 1624 | 698000 | 1396 | 36000 | 72 | 78000 | 156 | 620000 | 1240 | 29440 | 58,9 | 25920 | 51,8 | 169360 | 338,7 | 14 | Chicken |
| 143 | 224000 | 448 | 138000 | 276 | 24000 | 48 | 38000 | 76 | 100000 | 200 | 38720 | 77,4 | - | - | 124720 | 249,4 | 38,4 | Chicken and turkey |
| 144 | 1408000 | 2816 | 1092000 | 2184 | 72000 | 144 | 156000 | 312 | 936000 | 1872 | 3360 | 6,7 | - | - | 319360 | 638,7 | 22,5 | Turkey |
| 145 | 406000 | 812 | 125000 | 250 | 20000 | 40 | 86000 | 172 | 39000 | 780 | 38720 | 77,4 | 34560 | 69,1 | 354280 | 708,6 | 69,2 | Chicken |
| 146 | 620000 | 1240 | 430000 | 860 | 100000 | 200 | 90000 | 180 | 340000 | 680 | 11300 | 22,6 | 209040 | 418,1 | 410340 | 820,7 | 30,7 | Chicken and turkey |
| 147 | 88000 | 176 | 74000 | 148 | 4000 | 8 | 10000 | 20 | 64000 | 128 | 6160 | 12,3 | - | - | 20160 | 40,3 | 15,9 | Chicken |
| 148 | 1126000 | 2252 | 814000 | 1628 | 86000 | 172 | 226000 | 452 | 588000 | 1176 | 116160 | 232,3 | - | - | 428160 | 856,3 | 27,7 | Chicken, turkey and goose |
| 149 | 848000 | 1696 | 666000 | 1332 | 40000 | 80 | 142000 | 284 | 524000 | 1048 | 11040 | 22,1 | - | - | 193040 | 386,1 | 21,5 | Chicken and turkey |
| 150 | 800000 | 1600 | 708000 | 1416 | 36000 | 72 | 76000 | 152 | 632000 | 1264 | 6400 | 12,8 | - | - | 98400 | 196,8 | 11,5 | Chicken |
| 151 | 116000 | 232 | 60000 | 120 | 20000 | 40 | 36000 | 72 | 24000 | 48 | 35200 | 70,4 | - | - | 91200 | 182,4 | 48,3 | Chicken and turkey |
| 152 | 746000 | 1492 | 544000 | 1088 | 80000 | 160 | 122000 | 244 | 422000 | 844 | 32940 | 65,9 | - | - | 234940 | 469,9 | 27,1 |  |
| 153 | 694000 | 1388 | 476000 | 952 | 80000 | 160 | 126000 | 252 | 350000 | 700 | 10880 | 21,8 | - | - | 228880 | 457,8 | 31,4 |  |
| 154 | 496000 | 992 | 432000 | 864 | 28000 | 56 | 36000 | 72 | 396000 | 792 | 19200 | 38,4 | 16500 | 33 | 99700 | 199,4 | 12,9 | Chicken |
| 155 | 635600 | 1271,2 | 583600 | 1167,2 | 40000 | 80 | 52000 | 104 | 531600 | 1063,2 | 13440 | 46,9 | 15120 | 30,2 | 80560 | 161,1 | 8,2 |  |
| 156 | 56000 | 112 | 36000 | 72 | 8000 | 16 | 12000 | 24 | 24000 | 48 | 1440 | 2,9 | - | - | 21440 | 42,9 | 35,7 |  |
| 157 | 1268000 | 2536 | 846000 | 1692 | 160000 | 320 | 262000 | 524 | 584000 | 1168 | 32940 | 65,9 | - | - | 454940 | 909,9 | 33,3 | Chicken and turkey |
| 158 | 716000 | 1432 | 144000 | 288 | 24000 | 48 | 40000 | 80 | 104000 | 208 | 3840 | 7,7 | 14040 | 28 | 589880 | 1179,8 | 79,9 | Chicken |
| 159 | 912000 | 1824 | 820000 | 1640 | 44000 | 88 | 48000 | 96 | 772000 | 1544 | 48000 | 96 | - | - | 140000 | 280 | 10,1 |  |
| 160 | 776000 | 1552 | 546000 | 1092 | 52000 | 104 | 178000 | 356 | 368000 | 736 | 80960 | 161,9 | 323520 | 647 | 634480 | 1269 | 29,6 | Chicken and turkey |
| 161 | 1200200 | 2400,4 | 948800 | 1897,6 | 92400 | 184,8 | 243000 | 486 | 705800 | 1411,6 | 80960 | 161,9 | - | - | 332360 | 664,7 | 21 |  |
| 162 | 432000 | 864 | 414000 | 828 | 8000 | 16 | 10000 | 20 | 404000 | 808 | 1440 | 2,9 | - | - | 19440 | 38,9 | 4,2 | Chicken |
| 163 | 112000 | 224 | 92000 | 184 | 4000 | 8 | 16000 | 32 | 76000 | 152 | 8800 | 17,6 | - | - | 28800 | 57,6 | 17,9 |  |
| 164 | 428000 | 856 | 364000 | 728 | 12000 | 24 | 52000 | 104 | 312000 | 624 | 19200 | 38,4 | 20880 | 41,8 | 104080 | 208,2 | 15 |  |
| 165 | 765800 | 1531,6 | 605150 | 1210,3 | 32700 | 65,4 | 127950 | 255,9 | 477200 | 954,4 | 55200 | 110,4 | - | - | 215850 | 431,7 | 21 | Chicken and turkey |
| 166 | 1348000 | 2696 | 1078000 | 2156 | 72000 | 144 | 198000 | 396 | 880000 | 1760 | 38720 | 77,4 | 234000 | 468 | 542720 | 1085,4 | 20 | Turkey |
| 167 | 878000 | 1756 | 640000 | 1280 | 72000 | 144 | 166000 | 332 | 474000 | 948 | 18240 | 36,5 | 262000 | 524 | 518240 | 1036,5 | 27,1 | Chicken and turkey |
| 168 | 1084000 | 2168 | 808000 | 1616 | 116000 | 232 | 160000 | 320 | 648000 | 1296 | 56320 | 112,6 | - | - | 332320 | 664,6 | 25,5 |  |
| 169 | 786000 | 1572 | 645000 | 1290 | 30500 | 61 | 110500 | 221 | 534500 | 1069 | 115200 | 230,4 | - | - | 256200 | 512,4 | 17,9 | Chicken, turkey and goose |
| 170 | 652000 | 1304 | 444000 | 888 | 66000 | 132 | 142000 | 284 | 302000 | 604 | 110880 | 221,8 | 52800 | 105,6 | 371680 | 743,4 | 31,9 | Chicken and goose |
| 171 | 40800 | 81,6 | 20800 | 41,6 | 15200 | 30,4 | 4800 | 9,6 | 16000 | 32 | 1690 | 3,4 | - | - | 21690 | 43,4 | 49 | Chicken |
| 172 | 84000 | 168 | 62600 | 125,2 | 15000 | 30 | 6400 | 12,8 | 56200 | 112,4 | 29920 | 59,8 | - | - | 51320 | 102,6 | 25,5 | Chicken and turkey |
| 173 | 552700 | 1105,4 | 530900 | 1061,8 | 16400 | 32,8 | 5400 | 10,8 | 525500 | 1051 | 12800 | 25,6 | - | - | 34600 | 69,2 | 4 |  |
| 174 | 828000 | 1656 | 512000 | 1024 | 128000 | 256 | 188000 | 376 | 324000 | 648 | 97920 | 198,8 | - | - | 413920 | 827,8 | 38,2 |  |
| 175 | 349000 | 698 | 275000 | 550 | 31500 | 63 | 42500 | 85 | 232500 | 465 | 2535 | 5,1 | - | - | 76535 | 153,1 | 21,2 |  |
| 176 | 421500 | 843 | 387250 | 774,5 | 26800 | 53,6 | 7450 | 14,9 | 379800 | 759,6 | 77440 | 154,9 | - | - | 111690 | 223,4 | 8,1 | Chicken, turkey and goose |
| 177 | 145200 | 290,4 | 107950 | 215,9 | 23200 | 46,4 | 14050 | 28,1 | 93900 | 187,8 | 15840 | 31,7 | 3600 | 7,2 | 56690 | 113,4 | 25,7 | Chicken |
| 178 | 86400 | 172,8 | 64800 | 129,6 | 11400 | 22,8 | 10200 | 20,4 | 54600 | 109,2 | 26400 | 52,8 | - | - | 48000 | 96 | 25 | Chicken and goose |
| 179 | 563000 | 1126 | 423600 | 847,2 | 84500 | 169 | 54900 | 109,8 | 368700 | 737,4 | 32640 | 65,3 | - | - | 172040 | 344,1 | 24,8 | Chicken and turkey |
| 180 | 502200 | 1004,4 | 385300 | 770,6 | 56900 | 113,8 | 60000 | 120 | 325300 | 650,6 | 84640 | 169,3 | - | - | 201540 | 403,1 | 23,3 | Chicken, turkey and goose |
| 181 | 347800 | 695,6 | 233700 | 467,4 | 174900 | 349,8 | 47200 | 94,4 | 186500 | 373 | 21960 | 43,9 | - | - | 136060 | 272,1 | 32,8 | Chicken and turkey |
| 182 | 149000 | 298 | 108400 | 216,8 | 25500 | 51 | 15100 | 30,2 | 93300 | 186,6 | 5760 | 11,5 | - | - | 46360 | 92,7 | 27,3 | Chicken and goose |
| 183 | 330000 | 660 | 273000 | 546 | 37500 | 75 | 11500 | 23 | 261500 | 523 | 36960 | 73,9 | - | - | 93960 | 187,9 | 17,3 | Chicken, turkey and goose |
